# Longitudinal analysis of the relationship between motor and psychiatric symptoms in idiopathic dystonia

**DOI:** 10.1111/ene.15530

**Published:** 2022-09-11

**Authors:** Grace A. Bailey, Anna Rawlings, Fatemeh Torabi, William Owen Pickrell, Kathryn J. Peall

**Affiliations:** ^1^ Neuroscience and Mental Health Research Institute, Division of Psychological Medicine and Clinical Neurosciences Cardiff University School of Medicine Cardiff UK; ^2^ Swansea University Medical School Swansea UK; ^3^ Health Data Research London UK; ^4^ Department of Neurology, Morriston Hospital Swansea Bay University Health Board Swansea UK

**Keywords:** dystonia, movement disorders, neurological disorders, psychiatric disorders

## Abstract

**Background and purpose:**

Although psychiatric diagnoses are recognized in idiopathic dystonia, no previous studies have examined the temporal relationship between idiopathic dystonia and psychiatric diagnoses at scale. Here, we determine rates of psychiatric diagnoses and psychiatric medication prescription in those diagnosed with idiopathic dystsuponia compared to matched controls.

**Methods:**

A longitudinal population‐based cohort study using anonymized electronic health care data in Wales (UK) was conducted to identify individuals with idiopathic dystonia and comorbid psychiatric diagnoses/prescriptions between 1 January 1994 and 31 December 2017. Psychiatric diagnoses/prescriptions were identified from primary and secondary health care records.

**Results:**

Individuals with idiopathic dystonia (*n* = 52,589) had higher rates of psychiatric diagnosis and psychiatric medication prescription when compared to controls (*n* = 216,754, 43% vs. 31%, *p* < 0.001; 45% vs. 37.9%, *p* < 0.001, respectively), with depression and anxiety being most common (cases: 31% and 28%). Psychiatric diagnoses predominantly predated dystonia diagnosis, particularly in the 12 months prior to diagnosis (incidence rate ratio [IRR] = 1.98, 95% confidence interval [CI] = 1.9–2.1), with an IRR of 12.4 (95% CI = 11.8–13.1) for anxiety disorders. There was, however, an elevated rate of most psychiatric diagnoses throughout the study period, including the 12 months after dystonia diagnosis (IRR = 1.96, 95% CI = 1.85–2.07).

**Conclusions:**

This study suggests a bidirectional relationship between psychiatric disorders and dystonia, particularly with mood disorders. Psychiatric and motor symptoms in dystonia may have common aetiological mechanisms, with psychiatric disorders potentially forming prodromal symptoms of idiopathic dystonia.

## Graphical abstract

Individuals with idiopathic dystonia had higher rates of psychiatric diagnosis and psychiatric medication prescription when compared to controls, with depression and anxiety being most common. Psychiatric diagnoses predominantly predated dystonia diagnosis, particularly in the 12 months prior to diagnosis. There was, however, an elevated rate of most psychiatric diagnoses throughout the study period, including the 12 months after dystonia diagnosis, suggesting a bidirectional relationship between psychiatric disorders and dystonia.

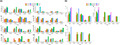

## INTRODUCTION

Idiopathic dystonia is a hyperkinetic movement disorder with an estimated prevalence of 1.2% [1]. It is characterized by sustained muscle contractions and abnormal postures, impacting both physical and social functioning [2]. Historically, dystonia was considered a psychiatric disorder, or labelled psychogenic, owing to the nature of the symptoms, which are often exacerbated by stress. Dystonia, as a diagnosis in itself, was initially proposed at the First International Dystonia Symposium (1975), with reference made to its nonprogressive nature and association with lesions of the nervous system [3]. Subsequently at least 25 Mendelian inherited genes have been identified and implicated in dystonia pathogenesis [4].

Prominent psychiatric symptoms have been recognized across the spectrum of dystonic disorders [5], with multiple studies extending from broad dystonia diagnostic groups [6, 7, 8, 9] to specific genetically inherited forms [10], with some focusing on specific psychiatric disorders [11, 12], and others utilizing a broader approach [13]. These studies have consistently identified elevated rates of depression and anxiety [9, 14, 15], as well as higher rates of phobias, notably social phobia and agoraphobia, and obsessive–compulsive disorder [7, 10].

Debate continues as to whether these psychiatric symptoms represent a primary phenotypic component of dystonia or are a secondary response to a chronic disabling disorder. Studies focused on genetically defined populations have provided some support for these being a component of the dystonia phenotype, with asymptomatic and symptomatic carriers of the DYT1 (*TOR1A* gene, encoding torsin A) mutation demonstrating an increased risk of early onset and recurrent depression compared to controls [11]. In addition, studies have shown stable psychiatric symptoms in the context of varying motor severity scores [15], onset of mood disorders prior to the emergence of motor symptoms [14, 15], and nonmotor symptoms not correlated with motor severity [16, 17].

Studies have yet to examine the rate and temporal relationship of psychiatric disorders at scale in idiopathic dystonia. Use of detailed population‐based data overcomes both recall and referral bias, and a longitudinal linkage approach has the potential to examine the association and subsequent risk of comorbid psychiatric disorders in pre‐ and post‐dystonia diagnostic periods. Using the Secure Anonymised Information Linkage (SAIL) Databank, this study represents the first to undertake case–control comparison, examining the rate and temporal pattern of psychiatric diagnoses and prescribed psychiatric medication in idiopathic dystonia.

## METHOD

### Study design and data sources

Using a cohort and case–control design, we investigated the rate of psychiatric diagnoses and medication in individuals diagnosed with dystonia, and its associated risk of psychiatric diagnoses within the SAIL Databank (Swansea University, UK: www.saildatabank.com). SAIL is a data repository containing anonymized, routinely collected health, education, and social care data covering the population of Wales. Clinical and demographic data are provided from several sources, including primary and secondary care records. Records can be linked between datasets for research purposes; the linkage procedures and approaches of the SAIL Databank have been reported previously [18].

Derivation and diagnostic validation of the dystonia cohort have been described elsewhere [1]. In brief, we developed a case‐ascertainment algorithm to identify individuals with idiopathic dystonia within primary and secondary care datasets. A reference population of 90 patients with a clinically confirmed diagnosis of adult onset idiopathic focal cervical dystonia (AOIFCD) was anonymously linked to records in SAIL. Codes relevant to dystonia were reviewed, and the final code list was created to maintain reasonable sensitivity (79% sensitivity). A person was defined as having a diagnosis of dystonia if their primary or secondary care records contained a Read code or International Classification of Diseases version 10 (ICD‐10) code for dystonia (Table S1). Using this previously validated method, 54,966 individuals with an idiopathic dystonia diagnosis were identified between January 1994 and December 2017. Figure S1 demonstrates derivation of the dystonia cohort. Our focus was on investigation of those with a diagnosis of idiopathic dystonia according to the most recent dystonia classification system [2], and therefore potential causes or diagnoses leading to a secondary dystonia were excluded from this cohort.

We obtained data on psychiatric diagnoses and prescribing information using primary care (Welsh Longitudinal General Practice dataset [WLGP]) and secondary care datasets (Patient Episode Database for Wales and Outpatient Dataset) between January 1994 and December 2017. Read codes can be used to attain information on diagnoses, prescriptions, symptoms, and administrative procedures, with previous work demonstrating the accuracy of primary care coding in the UK [19]. Primary care and secondary care data were available for ~80% and 100% of the Welsh population, respectively. We recorded demographic characteristics including age, sex, General Practice (GP) registration history, and deprivation index using the Welsh Index of Multiple Deprivation (WIMD) and the Welsh Demographic Data Service, which contains data on all persons registered with a primary care practice in Wales [20].

### Study cohort

The dystonia cohort consisted of 54,166 individuals diagnosed with dystonia between January 1994 and December 2017 [1]. This cohort was subdivided into those <20 years and ≥20 years of age at dystonia diagnosis to reflect the current dystonia classification system [2]. Dystonia cases were matched to a control population (*n* = 216,574) on year of birth, sex, year of study entry, year of follow‐up, and deprivation index quintile. Those with a primary and potential secondary cause of dystonia were excluded (Table S2). The majority of the dystonia cohort (*n* = 54,121, 99.9%) were randomly matched on a 1:4 (case:control) basis using those with WLGP records; however, this was not possible for the remaining 45 cases, each of which were match on a ratio of 1:1–3. Individuals prescribed an antipsychotic prior to a diagnosis of dystonia were excluded so as not to include potential cases of drug‐induced dystonia (*n* = 1577). Included individuals were required to be resident in Wales at the time of index date and have an age, sex, and GP registration date recorded. We defined the index date as the date of first dystonia diagnosis for dystonia cases and their respective matched controls. Due to the small sample size, those with "other" and "unspecified" dystonia were combined.

### Ascertainment of psychiatric diagnosis and prescriptions

An individual was defined as having a psychiatric disorder if their GP (primary care) or hospital record (secondary care) contained a previously validated Read version 2 or ICD‐10 diagnosis code (Table S3) [21]. We included codes for depression, anxiety, severe mental illness (SMI; schizophrenia, schizotypal and delusional disorders, bipolar disorder, other mood‐related disorders, and other severe mental illness), substance use disorder (SUD), eating disorders, attention‐deficit/hyperactivity disorder (ADHD), autism spectrum disorder (ASD), and conduct disorder. Use of hypnotics, antidepressants, anxiolytics, and antipsychotics was obtained from primary GP records (Table S4) without inference to their diagnosis [22, 23]. Individuals with dystonia who were prescribed a benzodiazepine (Table S5) and had no recorded psychiatric diagnoses were considered to have no recorded psychiatric medication due to benzodiazepines, at times, being used to manage the motor symptoms of dystonia. The majority of medications are electronically prescribed by GPs, whereby a Read code is entered for each prescription (e.g., monthly). Diagnoses and prescription records were obtained throughout the study period (January 1994–December 2017), although only those recorded 12 years before to 12 years after the index date were analysed. In an attempt not to bias findings, we limited our analysis to this period due to differences in data availability dependent upon when an individual received a dystonia diagnosis (Figure S2). Psychiatric outcomes were examined individually and combined as all psychiatric diagnoses/prescriptions when investigating first‐time psychiatric events.

### Deprivation score

Deprivation scores were derived using WIMD 2014 quintiles according to Lower Super Output Area (LSOA) of residence (2011). WIMD identifies and ranks all LSOAs in Wales from 1 (most deprived) to 1909 (least deprived); each LSOA is then grouped into quintiles (1 = most deprived, 5 = least deprived) [20]. The WIMD quintile was assessed at three dates, entry into the study dataset, index date, and the end of the study (December 2017), deregistration from GP, moving out of Wales, or the time of death.

### Statistical analyses

Data were analysed using R software (v4.1.3). Dystonia and control groups were compared using chi‐squared tests. Poisson regression was used to calculate the incidence rate ratio. Psychiatric diagnoses and prescriptions occurring on the same day as the index date were included in the year before the index date time period. Logistic regression was used to calculate the association of psychiatric disorders or prescribed psychiatric drugs and the risk of idiopathic dystonia, expressed as odds ratios (ORs), both with 95% confidence intervals (CIs) and with Bonferroni correction (*p* = 0.002 and *p* = 0.005, respectively). Regression analyses were adjusted for time in study. Wilcoxon signed‐rank tests were used to determine changes in WIMD quintile.

## RESULTS

A total of 28,666 idiopathic dystonia cases (54.5%) and 96,705 controls (44.7%) were identified as having a psychiatric diagnosis or prescription. Of these, 22,682 cases (43.1%) and 67,458 (31.1%) controls had a new psychiatric diagnosis, and 23,793 cases (45.2%) and 82,096 (37.9%) controls were prescribed a new psychiatric medication, both demonstrating significantly higher levels in the dystonia cohort compared to controls (*p* < 0.001; Table 1).

**TABLE 1:** Demographics for cases and controls with a first‐time psychiatric diagnosis or medication during the study period

Characteristic	Overall, *N* = 52,589, *n* (%)	Cervical dystonia, *N* = 36,341, *n* (%)	Blepharospasm, *N* = 1262, *n* (%)	Tremor, *N* = 14,308, *n* (%)	Other, *N* = 578, *n* (%)	Controls, *N* = 216,574, *n* (%)
Overall first‐time psychiatric diagnoses	**21,354 (40.6)**	**13,943 (38.4)**	**460 (36.5)**	**6667 (46.6)**	**251 (43.4)**	64,084 (29.6)
Predystonia	**12,095 (56.6)**	7194 (51.6)	**280 (60.9)**	**4427 (66.4)**	**177 (70.5)**	33,551 (52.4)
Postdystonia	**9259 (43.4)**	6749 (48.4)	**180 (39.1)**	**2240 (33.6)**	**74 (29.5)**	30,533 (47.6)
Sex						
Male	6693 (31.3)	**4075 (29.2)**	141 (30.7)	**2376 (35.6)**	94 (37.5)	19,823 (30.9)
Female	14,661 (68.7)	**9868 (70.8)**	319 (69.3)	**4291 (64.4)**	157 (62.5)	44,261 (69.1)
Aged ≥20 years	17,542 (82.1)	**11,077 (79.4)**	**435 (94.6)**	**5793 (86.9)**	211 (84)	52,366 (81.7)
Aged <20 years	3812 (17.9)	**2866 (20.6)**	**25 (5.4)**	**874 (13.1)**	40 (15.9)	11,718 (18.3)
Aged ≥20 years						
Predystonia	**11,207 (63.9)**	6628 (59.8)	<>	**4135 (71.4)**	**153 (72.5)**	31,201 (59.6)
Postdystonia	**6335 (36.1)**	4449 (40.2)	<>	**1658 (28.6)**	**58 (27.5)**	21,165 (40.4)
Aged <20 years						
Predystonia	**888 (23.3)**	566 (19.7)	<>	**292 (33.4)**	**24 (60)**	2350 (20.05)
Postdystonia	**2924 (76.7)**	2300 (80.3)	<>	**582 (66.6)**	**16 (40)**	9368 (79.95)
Overall psychiatric diagnoses	**22,682 (43.1)**					67,458 (31.1)
Depression	**16,030 (30.5)**	**10,482 (28.8)**	**344 (27.3)**	**4985 (34.8)**	**194 (33.6)**	46,567 (21.5)
Anxiety	**13,468 (25.6)**	**8425 (23.2)**	**319 (25.3)**	**4554 (31.8)**	**150 (26)**	36,466 (16.8)
Substance use disorder	**3764 (7.2)**	**2247 (6.2)**	78 (6.1)	**1374 (9.6)**	**59 (10.2)**	11,776 (5.4)
Eating disorder	**933 (1.8)**	**556 (1.5)**	<>	**357 (2.5)**	<>	2345 (1.1)
Severe mental illness	**483 (0.9)**	**210 (0.6)**	<>	**234 (1.6)**	**24 (4.2)**	2565 (1.2)
Conduct disorder	**295 (0.6)**	**213 (0.6)**	<>	72 (0.5)	<>	830 (0.4)
ADHD	**279 (0.5)**	**196 (0.5)**	<>	73 (0.5)	<>	824 (0.3)
ASD	**241 (0.5)**	153 (0.4)	<>	79 (0.5)	<>	763 (0.4)
Predystonia						
Depression	**8802 (16.7)**	**5255 (14.5)**	**204 (16.2)**	**3201 (22.4)**	**130 (22.5)**	23,695 (10.9)
Anxiety	**6861 (13)**	**3845 (10.6)**	**187 (14.8)**	**2728 (19.1)**	**92 (15.9)**	17,080 (7.9)
Substance use disorder	**1999 (3.8)**	1070 (2.9)	<>	**839 (5.9)**	**39 (6.7)**	6042 (2.8)
Eating disorder	**487 (0.9)**	**287 (0.8)**	<>	**187 (1.3)**	<>	1185 (0.5)
Conduct disorder	**168 (0.3)**	**120 (0.3)**	<>	<>	<>	518 (0.2)
Severe mental illness	**163 (0.3)**	**52 (0.1)**	<>	96 (0.7)	<>	1277 (0.6)
ADHD	**137 (0.3)**	**99 (0.3)**	<>	<>	**7 (1.2)**	385 (0.2)
ASD	94 (0.2)	63 (0.2)	<>	<>	<>	332 (0.2)
Postdystonia						
Depression	**7228 (13.7)**	**5227 (14.4)**	140 (11.1)	**1784 (12.5)**	64 (11.1)	22,872 (10.6)
Anxiety	**6607 (12.6)**	**4580 (12.6)**	132 (10.5)	**1826 (12.8)**	58 (10)	19,386 (9)
Substance use disorder	**1765 (3.4)**	**1177 (3.2)**	<>	**535 (3.7)**	20 (3.5)	5734 (2.6)
Eating disorder	**446 (0.8)**	**269 (0.7)**	<>	**170 (1.2)**	<>	1160 (0.5)
Severe mental illness	320 (0.6)	**158 (0.4)**	<>	**138 (1)**	<>	1288 (0.6)
ASD	**147 (0.3)**	90 (0.2)	<>	<>	<>	431 (0.2)
ADHD	142 (0.3)	97 (0.3)	<>	<>	<>	439 (0.2)
Conduct disorder	**127 (0.2)**	**93 (0.3)**	<>	<>	<>	312 (0.1)
Anxiety disorders						
Panic	**1367 (2.6)**	**862 (2.4)**	28 (2.2)	**454 (3.2)**	<>	3214 (1.5)
Specific isolated phobia	**454 (0.9)**	**316 (0.9)**	<>	**116 (0.8)**	<>	205 (0.1)
Generalized	**326 (0.6)**	**204 (0.6)**	<>	**112 (0.8)**	<>	876 (0.4)
Other/unspecified anxiety	**285 (0.5)**	**178 (0.5)**	8 (0.6)	**94 (0.7)**	5 (0.9)	717 (0.3)
Mixed anxiety	**222 (0.4)**	135 (0.4)	<>	**78 (0.5)**	<>	710 (0.3)
OCD	170 (0.3)	113 (0.3)	<>	47 (0.3)	<>	597 (0.3)
Agoraphobia	83 (0.2)	49 (0.1)	<>	<>	<>	277 (0.1)
Other/unspecified phobia	71 (0.1)	48 (0.1)	<>	<>	‐	205 (0.1)
Social	17 (0.03)	9 (0.02)	‐	8 (0.06)	<>	50 (0.02)
Childhood anxiety	7 (0.01)	7 (0.02)	‐	‐	‐	14 (0.01)
Overall first‐time psychiatric prescriptions	**21,996 (41.8)**	**14,384 (39.6)**	**519 (41.1)**	**6760 (47.2)**	**295 (51)**	77,757 (35.9)
Predystonia	**13,565 (61.7)**	**8296 (57.7)**	**341 (65.7)**	**4698 (69.5)**	**211 (71.5)**	39,993 (51.4)
Postdystonia	**8431 (38.3)**	**6088 (42.3)**	**178 (34.3)**	**2062 (30.5)**	**84 (28.5)**	37,764 (48.6)
Sex						
Male	6705 (30.5)	**4002 (27.8)**	156 (30.1)	**2424 (35.9)**	**112 (38)**	23,117 (29.7)
Female	**15,291 (69.5)**	**10,382 (72.2)**	363 (69.9)	**4336 (64.1)**	**183 (62)**	54,640 (70.3)
Aged ≥20 years	**19,224 (87.4)**	**12,287 (85.4)**	**500 (96.3)**	**6142 (90.9)**	262 (88.8)	68,918 (88.6)
Aged <20 years	**2772 (12.6)**	**2097 (14.6)**	**19 (3.7)**	**618 (9.1)**	33 (11.2)	8839 (11.4)
Aged ≥20 years						
Predystonia	**13,105 (68.2)**	**7975 (64.9)**	<>	**4575 (74.5)**	<>	39,171 (56.8)
Postdystonia	**6119 (31.8)**	**4312 (35.1)**	<>	**1567 (25.5)**	<>	29,747 (43.2)
Aged <20 years						
Predystonia	**460 (16.6)**	**321 (15.3)**	<>	**123 (19.9)**	<>	822 (9.3)
Postdystonia	**2312 (83.4)**	**1776 (84.7)**	<>	**495 (80.1)**	<>	8017 (90.7)
Psychiatric prescriptions during study period, *n*	1,246,558	613,347	29,675	576,244	25,049	3,845,023
Psychiatric prescriptions per person, mean *n* (SD)	**50.4 (98.7)**	**39.1 (76.5)**	**49.1 (85.7)**	**71.4 (128.4)**	**73.7 (129.1)**	45.06 (108.6)
Different medications prescribed, mean *n* (SD)	**3.2 (2.5)**	**3.1 (2.4)**	**3 (2.4)**	**3.5 (2.7)**	**3.4 (2.4)**	2.62 (2.2)
Overall psychiatric prescriptions	**29,426 (56)**					82,096 (37.9)
Antidepressants	**20,049 (38.1)**	**12,901 (35.5)**	**466 (36.9)**	**6390 (44.7)**	**261 (45.2)**	64,830 (29.9)
Anxiolytics	**11,297 (21.5)**	**7401 (20.4)**	**243 (19.3)**	**3453 (24.1)**	**178 (30.8)**	30,519 (14.1)
Hypnotics	**8233 (15.7)**	**5075 (14)**	**200 (15.8)**	**2830 (19.8)**	**117 (20.2)**	26,354 (12.2)
Antipsychotics	**1164 (2.2)**	**575 (1.6)**	<>	**520 (3.6)**	<>	6086 (2.8)
Predystonia						
Antidepressants	**11,221 (21.3)**	**6509 (17.9)**	**287 (22.7)**	**4227 (29.5)**	**183 (31.7)**	32,135 (14.8)
Anxiolytics	**6944 (13.2)**	**4513 (12.4)**	**135 (10.7)**	**2177 (15.2)**	**107 (18.5)**	13,615 (6.3)
Hypnotics	**4188 (8)**	2259 (6.2)	<>	**1745 (12.2)**	<>	12,875 (5.9)
Antipsychotics	<>	<>	<>	<>	<>	2521 (1.2)
Postdystonia						
Antidepressants	**8828 (16.8)**	**6392 (17.6)**	179 (14.2)	2163 (15.1)	78 (11.8)	32,695 (15.1)
Anxiolytics	**4353 (8.3)**	2888 (7.9)	108 (8.6)	**1276 (8.9)**	**71 (10.8)**	16,904 (7.8)
Hypnotics	**4045 (7.7)**	**2816 (7.7)**	<>	**1085 (7.6)**	<>	13,479 (6.2)
Antipsychotics	<>	<>	<>	<>	<>	3565 (1.6)
Most commonly prescribed anxiolytics during the study period						
1	**Diazepam: 10,341 (91.5)**	**Diazepam: 6887 (93.1)**	Diazepam: 220 (90.5)	Diazepam: 3060 (88.6)	Diazepam: 156 (87.6)	Diazepam: 26,806 (87.8)
2	Hydroxyzine: 860 (7.6)	**Hydroxyzine: 514 (6.9)**	Hydroxyzine: <>	Hydroxyzine: 302 (8.7)	Hydroxyzine: <>	Hydroxyzine: 2454 (8)
3	**Buspirone: 470 (4.2)**	Buspirone: 253 (3.4)	Lorazepam: <>	**Chlordiazepoxide: 231 (6.7)**	Lorazepam: <>	Lorazepam: 1785 (5.8)
4	**Lorazepam: 458 (4.1)**	**Lorazepam: 199 (2.7)**	Buspirone: 11 (4.5)	Lorazepam: 230 (6.7)	Buspirone: <>	Buspirone: 999 (3.3)
5	**Chlordiazepoxide: 422 (3.7)**	Chlordiazepoxide: 180 (2.4)	Oxazepam: 5 (2.1)	**Buspirone: 195 (5.6)**	Chlordiazepoxide: 5 (2.8)	Chlordiazepoxide: 882 (2.9)
Most commonly prescribed antidepressants during the study period						
1	**Citalopram: 10,638 (53.1)**	**Citalopram: 6821 (52.9)**	Citalopram: 213 (45.7)	**Citalopram: 3467 (54.3)**	Amitriptyline: 118 (45.2)	Citalopram: 31,290 (48.3)
2	**Amitriptyline: 7818 (39)**	**Amitriptyline: 4869 (37.7)**	**Amitriptyline: 209 (44.8)**	**Amitriptyline: 2612 (40.9)**	**Citalopram 116 (44.4)**	Amitriptyline: 21,646 (33.4)
3	**Fluoxetine: 6767 (33.8)**	**Fluoxetine: 4469 (34.6)**	Fluoxetine: 148 (31.8)	**Fluoxetine: 2060 (32.2)**	Fluoxetine: <>	Fluoxetine: 19,005 (29.3)
4	**Sertraline: 4736 (23.6)**	**Sertraline: 2916 (22.6)**	Sertraline: 94 (20.2)	**Sertraline: 1668 (26.1)**	Sertraline: <>	Sertraline: 13,572 (20.9)
5	**Mirtazapine: 3564 (17.8)**	Mirtazapine: 2116 (16.4)	Mirtazapine: 58 (12.4)	**Mirtazapine: 1338 (20.9)**	Mirtazapine: <>	Mirtazapine: 10,290 (15.9)

*Note*: Percentages can be >100% where participants had more than one diagnosis/prescription. P‐values are all versus controls. Bold values represent significant values after Bonferroni correction for multiple comparisons. <> masked to prevent identification of small numbers, ‐ represents no data available.

Abbreviations: ADHD, attention‐deficit/hyperactivity disorder; ASD, autism spectrum disorder; OCD, obsessive–compulsive disorder.

### Psychiatric diagnoses

**FIGURE 1:**
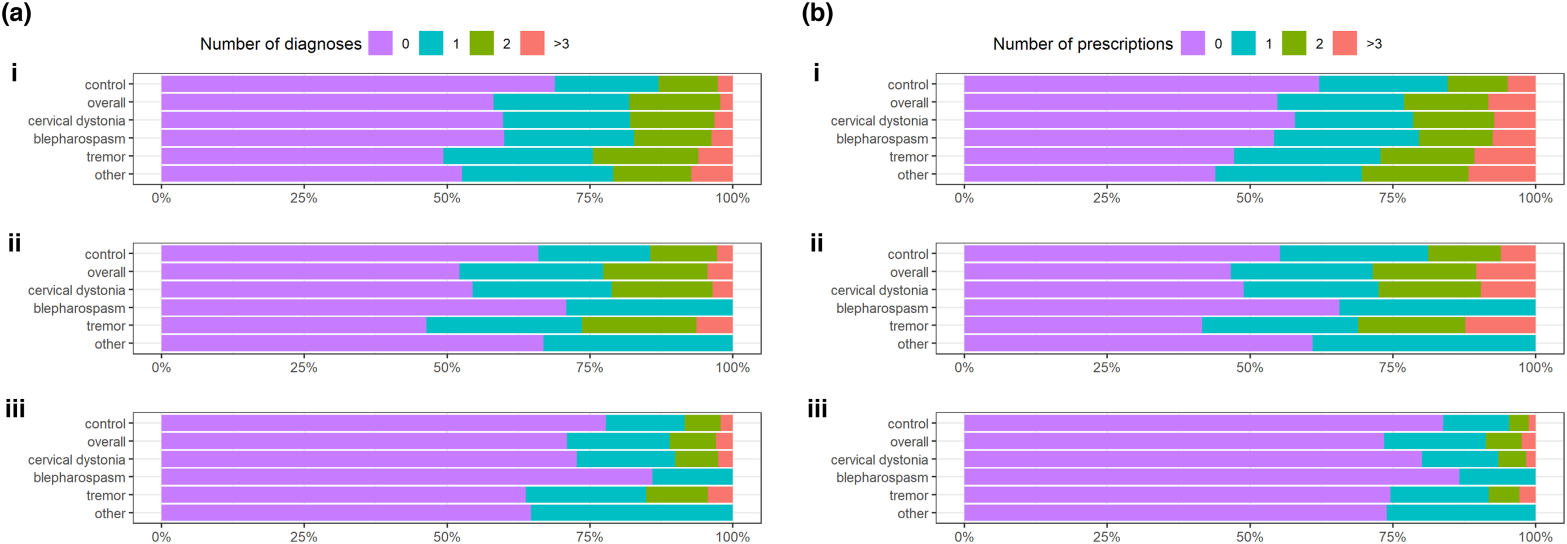
(a) Number of different diagnoses or (b) medications in the case and control groups by age group: i, overall; ii, ≥20 years old at index date; and iii, <20 years old at index date [Colour figure can be viewed at wileyonlinelibrary.com

**FIGURE 2:**
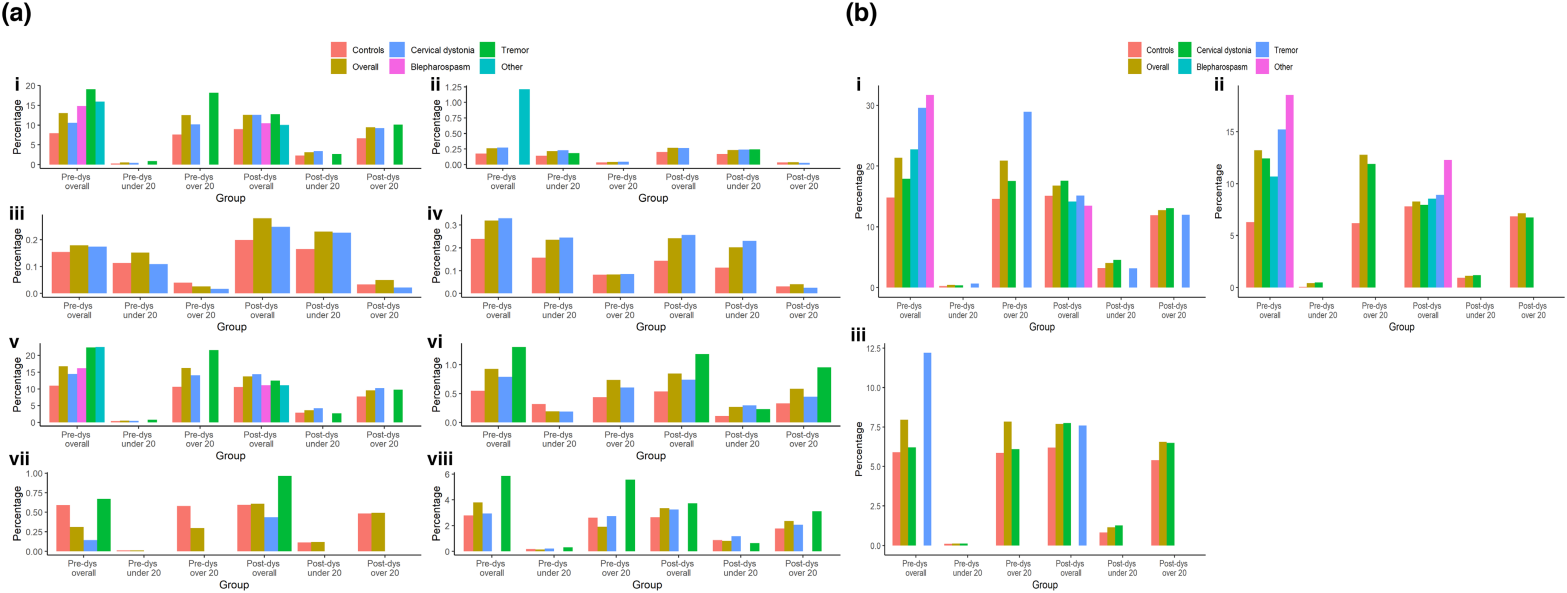
(a) Proportions of diagnoses by age and time for cases and controls: i, anxiety disorder; ii, attention‐deficit/hyperactivity disorder; iii, autism spectrum disorder; iv, conduct disorder; v, depression; vi, eating disorder; vii, severe mental illness; and viii, substance use disorder. (b) Proportions of prescriptions by age and time for cases and controls: i, antidepressants; ii, anxiolytics; and iii, hypnotics. Percentages can be >100% where participants had more than one diagnosis. dys, dystonia diagnosis [Colour figure can be viewed at wileyonlinelibrary.com

Individuals with idiopathic dystonia and all subtypes were significantly more likely to be diagnosed with multiple (two or more) psychiatric diagnoses when compared to controls (*p* < 0.001; Figure 1ai). In those with dystonia, cervical dystonia, and tremor, a higher frequency of multiple diagnoses was also observed in both age categories but was greater in those ≥20 years old at diagnosis (*p* < 0.001; Figure 1aii, Table S6). The proportions of diagnoses by age, pre‐ and postdiagnosis, are shown in Figure 2a. First recorded diagnoses of anxiety (*p* = 0.002), conduct disorder (*p* < 0.001), depression (*p* < 0.001), and SUD (*p* < 0.001) predated dystonia diagnosis, whereas a higher rate of ASD and SMI (*p* < 0.001) diagnoses postdated overall dystonia. A comparable incidence of ADHD (*p* = 0.7) and eating disorder (*p* = 0.06) was observed before and after dystonia diagnosis (Table 1). Similar patterns were observed among those with dystonic tremor, whereas depression, anxiety, and SUD predated blepharospasm and other dystonia (*p* < 0.001). Interestingly, incidence of depression was comparable (*p* = 0.7) and higher rates of anxiety were observed after cervical dystonia diagnosis (*p* < 0.001).

### Prescriptions

Two or more psychiatric medications were prescribed at a higher rate among the dystonia cohort compared to controls (*p* < 0.001; Figure 1bi), with an increased frequency of multiple prescriptions in both age groups (Figure 1bii, iii), observed to a greater extent in those ≥20 years at diagnosis (*p* < 0.001). Significantly higher rates of prescription were also observed before index diagnosis within the dystonia cohort, compared to after dystonia diagnosis across antidepressants and anxiolytics (Table 1, Figure 2b). By contrast, antipsychotics postdated dystonia diagnosis (*p* < 0.001), and hypnotics were equally distributed before and after dystonia diagnosis (*p* = 0.03). Antidepressants predated all subtypes of dystonia except for cervical dystonia, where higher frequencies were observed postdiagnosis (*p* = 0.1).

### Time between diagnoses

**FIGURE 3:**
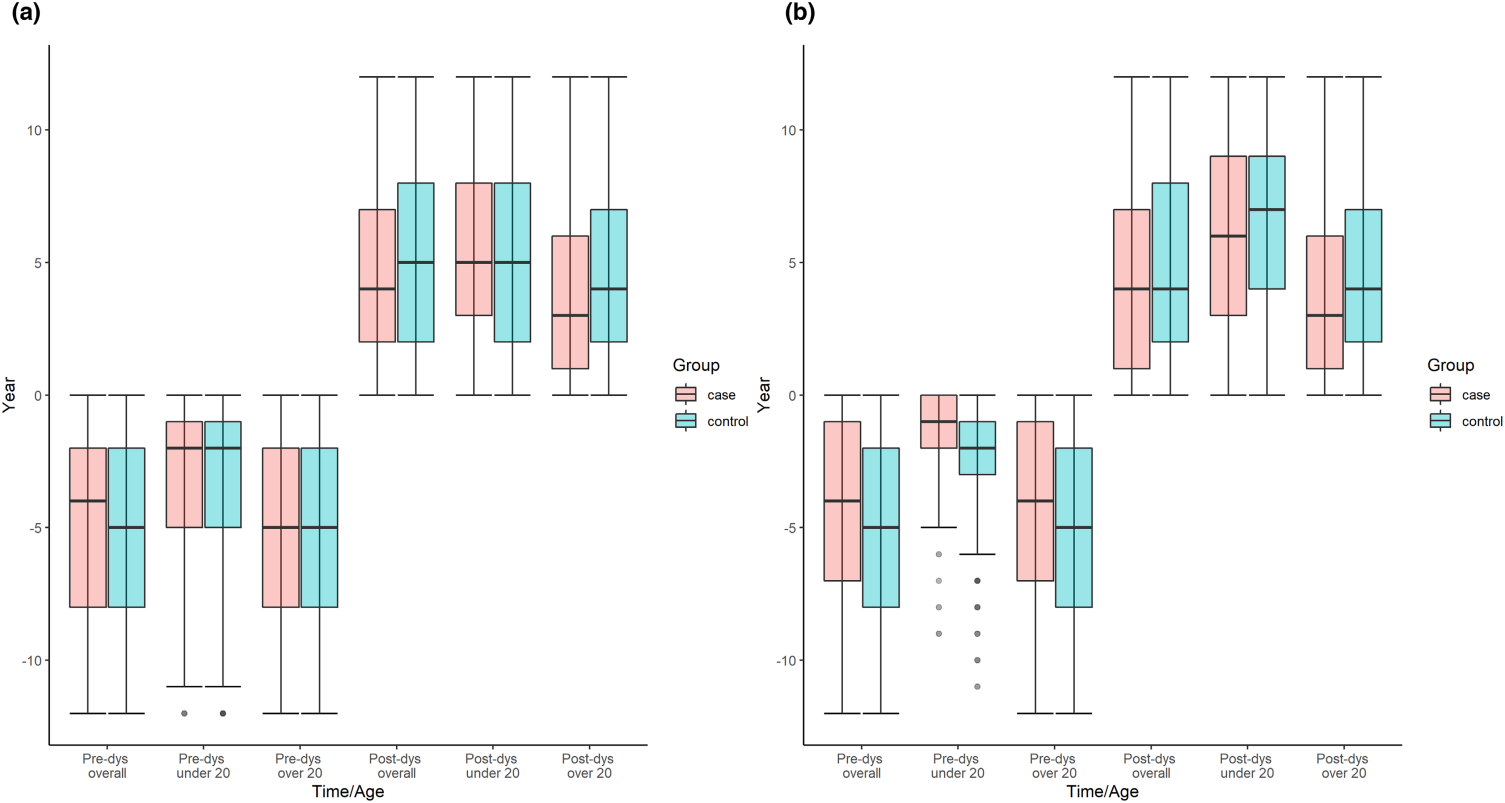
Median time (years) from first (a) psychiatric diagnoses or (b) medication to index date. dys, dystonia diagnosis [Colour figure can be viewed at wileyonlinelibrary.com

The time (years) between first psychiatric diagnosis/prescription and index date was significantly shorter in dystonia overall, cervical dystonia, and tremor compared to controls, with a significantly shorter time interval to psychiatric diagnosis/prescription seen after index date, and shortest for those ≥20 years old at dystonia diagnosis (Table S7 and Figure 3).

### Deprivation

**FIGURE 4:**
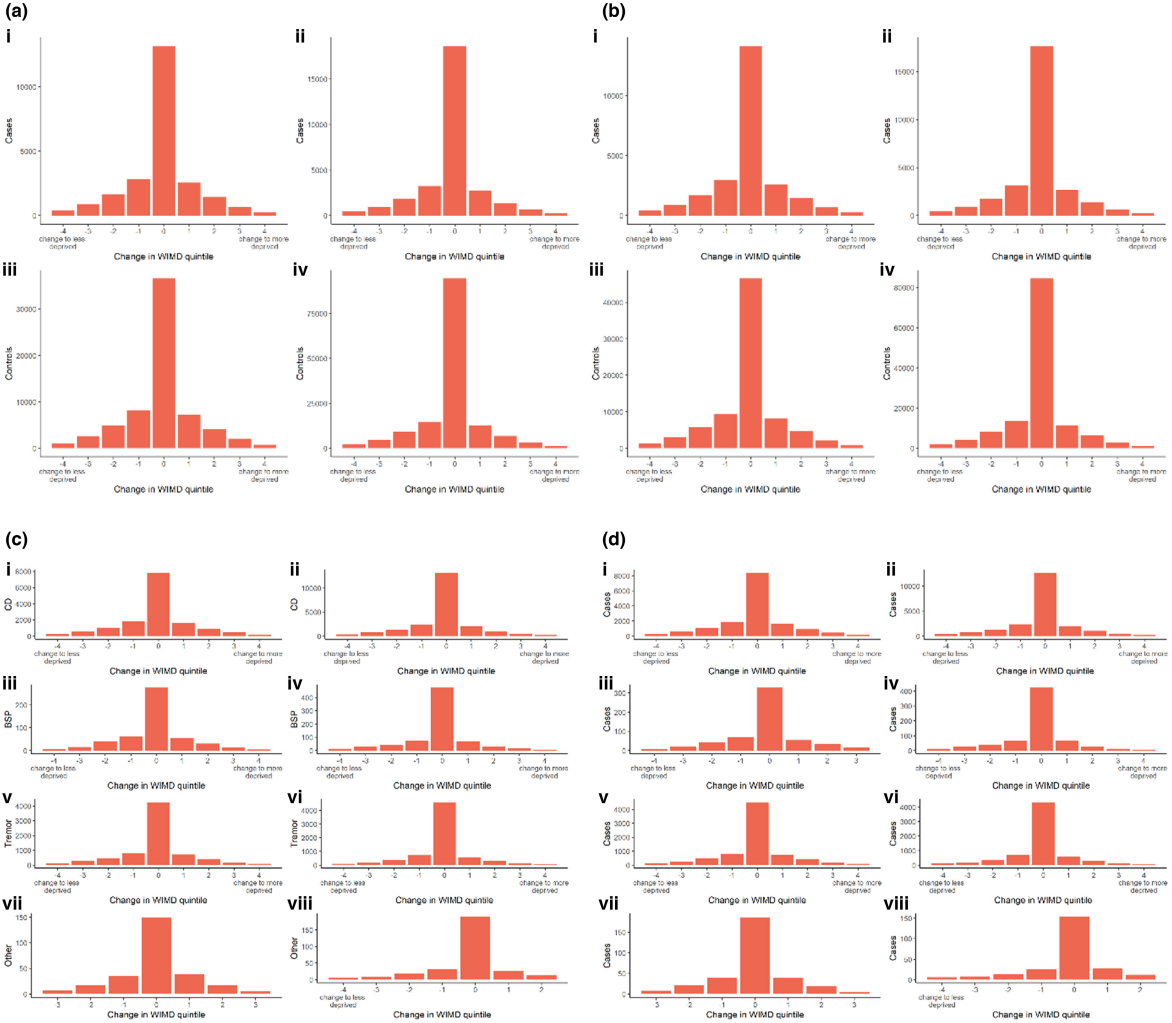
Change in Welsh Index of Multiple Deprivation (WIMD) quintile at entry in dataset and follow‐up for controls and dystonia cases by subtype: psychiatric diagnoses in cases (a; i), dystonia subtypes (c), and controls (a; iii); no psychiatric diagnoses in cases (a; ii) and controls (a; iv); psychiatric prescriptions in cases (b; i), dystonia subtypes (d), and controls (b, iii); and no psychiatric prescriptions in cases (b; ii) and controls (b; iv). BSP, blepharospasm; CD, cervical dystonia [Colour figure can be viewed at wileyonlinelibrary.com

There were no changes in deprivation quintile between entry into the dataset and follow‐up for cases with and without psychiatric diagnoses/prescriptions (Figure 4), with this pattern replicated in the cervical dystonia, blepharospasm, tremor, other dystonia, and control cohorts.

### Association between dystonia and psychiatric diagnosis/prescription

Individuals with dystonia had an increased risk for aggregated psychiatric diagnoses and all specific psychiatric disorders, except for reduced risk for SMI (Table S8A). Those with dystonia had a higher risk of anxiety, eating disorders, and depression (adjusted [aOR] = 1.64, 95% CI = 1.61–1.68; aOR = 1.62, 95% CI = 1.5–1.75; aOR = 1.55, 95% CI = 1.51–1.58, respectively). Those ≥20 years old had an increased risk of psychiatric diagnoses when compared to those <20 years old (aOR = 1.72, 95% CI = 1.68–1.76 vs. aOR = 1.4, 95% CI = 1.34–1.47). Risk of anxiety and depression was also higher among those with cervical dystonia (aOR = 1.41, 95% CI = 1.37–1.45; aOR = 1.41, 95% CI = 1.37–1.44) and blepharospasm (aOR = 1.64, 95% CI = 1.44–1.86; aOR = 1.34, 95% CI = 1.18–1.52), and nearly twice as high in other forms of dystonia (aOR = 1.82, 95% CI = 1.5–2.19; aOR = 1.95, 95% CI = 1.63–2.32) and dystonic tremor (aOR = 2.35, 95% CI = 2.26–2.44; aOR = 1.98, 95% CI = 1.91–2.06; Table S8B–E). Interestingly, those with other/unspecified dystonia were at highest risk for SMI (aOR = 3.67, 95% CI = 2.37–5.4) and ADHD (aOR = 3.66, 95% CI = 1.66–6.89). ASD was not associated with subtypes of dystonia, except for dystonic tremor (aOR = 1.58, 95% CI = 1.24–1.98).

Anxiolytic prescriptions were almost two times higher (aOR = 1.61, 95% CI = 1.57–1.65) in dystonia patients, whereas risk was lower but comparable for prescription of antidepressants and hypnotics (aOR = 1.39, 95% CI = 1.36–1.42; aOR = 1.3, 95% CI = 1.26–1.33). Interestingly, among dystonia overall and cervical dystonia, risk of use of antipsychotics was reduced (aOR = 0.77, 95% CI = 0.72–0.82; aOR = 0.54, 95% CI = 0.5–0.6), whereas risk was increased in other dystonia and tremor (aOR = 1.98, 95% CI = 1.35–2.8; aOR = 1.3, 95% CI = 1.19–1.43; Table S9). However, we excluded individuals who were prescribed an antipsychotic medication prior to their dystonia diagnosis so as not to include potential cases of drug‐induced dystonia.

### Comparative risk of psychiatric diagnosis and/or prescription of psychiatric medication

**FIGURE 5:**
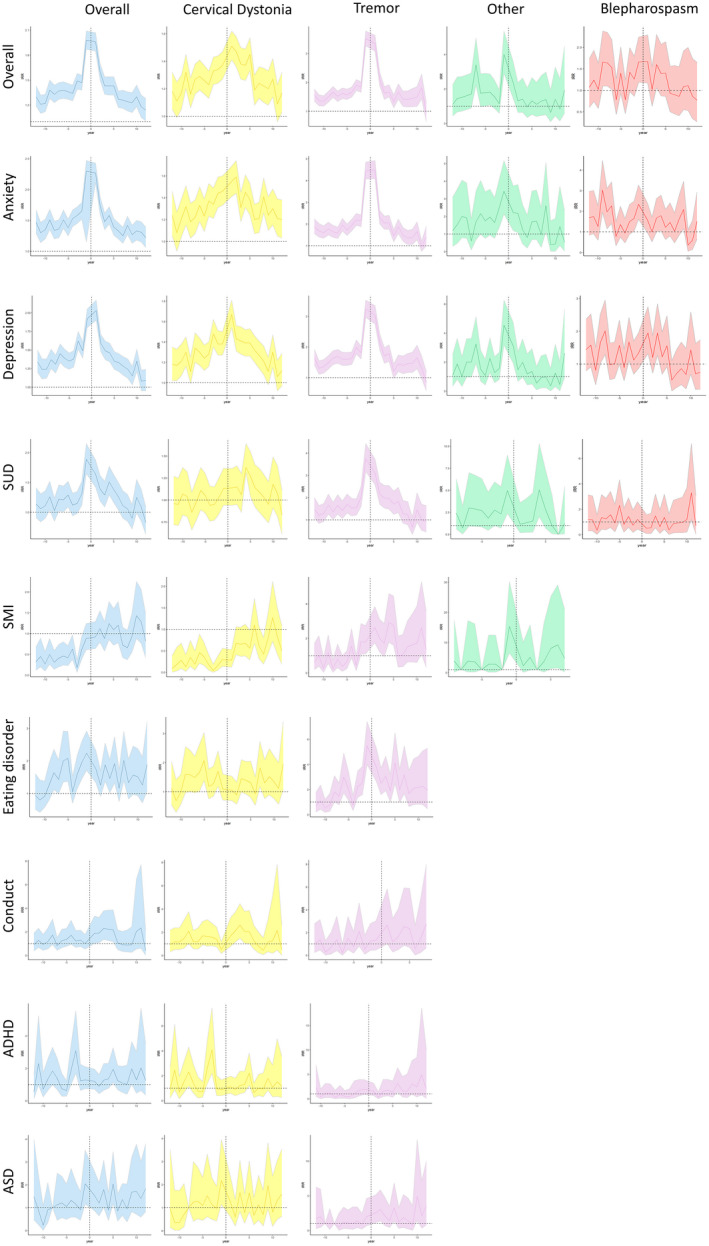
Trends in psychiatric diagnosis incidence rate ratios (IRRs) for dystonia subtypes in relation to controls, with 95% confidence intervals. ADHD, attention‐deficit/hyperactivity disorder; ASD, autism spectrum disorder; SMI, severe mental illness; SUD, substance use disorder [Colour figure can be viewed at wileyonlinelibrary.com

**FIGURE 6:**
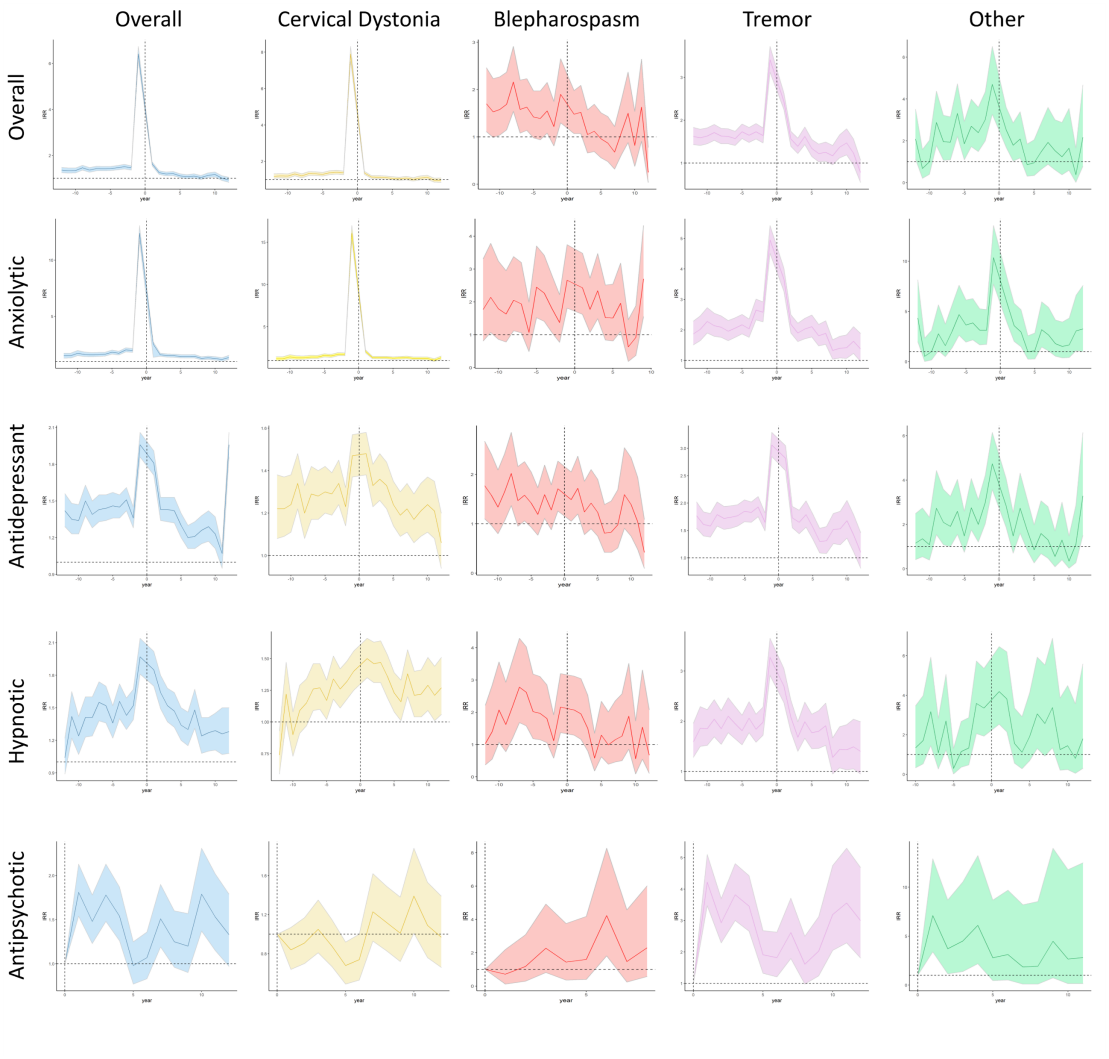
Trends in psychiatric diagnosis incidence rate ratios (IRRs) for dystonia subtypes in relation to controls, with 95% confidence intervals [Colour figure can be viewed at wileyonlinelibrary.com

There was an increased risk of psychiatric diagnosis and prescription in those with dystonia across the entire study period, with this increasing in the year on either side of dystonia diagnosis. A similar pattern was observed for anxiety and depression, and their associated oral medical therapies, whereas other psychiatric diagnoses (e.g., SMI and eating disorders) demonstrated a more consistent increased risk (Figures 5 and 6).

## DISCUSSION

This large data linkage study demonstrates a significantly higher rate of psychiatric diagnoses and/or prescription of medication in individuals diagnosed with dystonia, compared to matched controls. This difference was greater in the period predating a dystonia diagnosis.

We found higher rates of all psychiatric disorders in those with idiopathic dystonia and subtypes, with depression and anxiety being the most commonly diagnosed, and panic disorder (2.6%), specific isolated phobia (0.9%), and generalized anxiety (0.6%) the most common anxiety subtypes, comparable to that seen in multiple cross‐sectional studies of idiopathic dystonia [14, 24, 25]. The rates of psychiatric diagnoses within the control population were in keeping with those estimated within the UK, including depression and anxiety disorders (21.4%) [23], ASD (0.5%) [26], and severe mental illness (0.7%) [27]. Among the dystonia cohort and when broken down by subtypes, antidepressants and anxiolytics were the most frequently prescribed psychiatric medication. A higher number of individuals were prescribed medication compared to those who received a diagnosis (56% vs. 43%), potentially relating to the stigma associated with a psychiatric diagnosis, or because a prescription might imply a diagnosis. Alternatively, medications may have been prescribed for a nonpsychiatric diagnoses such as neuropathic pain syndromes.

We noted higher rates of neurodevelopmental disorders and conduct disorder in the <20‐year‐old dystonia group compared to the ≥20‐year‐old group, whereas depression, anxiety, SMI, and SUD were increased in the ≥20‐year‐old group. This likely reflects commonality in diagnoses; ASD, ADHD, and conduct disorders are considered in childhood/adolescence and tend to lessen with age [28], whereas rates of depression, anxiety, and psychotic disorders are more pronounced in adults [29]. Although neurodevelopmental disorders can persist into adulthood, symptoms may be interpreted differently, and this often results in misdiagnosis [30, 31].

Dystonia was also associated with a higher risk of all psychiatric diagnoses, with the exception of SMI, most notably anxiety disorder, eating disorder, and depression (aOR = 1.64, 1.62, and 1.55, respectively). Anxiolytics, antidepressants, and hypnotics were also associated with higher risk (aOR = 1.61, 1.39, and 1.3, respectively), whereas antipsychotics were associated with reduced risk (aOR = 0.77). These results are consistent with the single other population‐based study undertaken to date, which showed increased risk for depression, anxiety, ADHD, and ASD [24]. The presence of a psychiatric diagnosis or psychiatric prescription, however, does not appear to impact social deprivation, suggesting neither social drift nor causation is evident with dystonia, similarly observed in other neurological and psychiatric disorders [27, 32].

Our study is the first to investigate psychiatric comorbidity both before and after dystonia diagnosis. The majority of work to date has been limited to the period after dystonia diagnosis, with preceding data relying on recall and anecdotal information. These studies have estimated rates of 40%–69% of preceding mood disorders [14], consistent with our findings, in which 57% of new psychiatric diagnoses preceded the dystonia diagnosis. We also demonstrated a significantly increased risk of a psychiatric diagnosis immediately before and after dystonia diagnosis. This pattern is replicated across multiple specific diagnoses, particularly anxiety, in which the risk of developing an anxiety disorder is highest in the 5 years leading to dystonia diagnosis. Although this may reflect the recognized diagnostic delay with dystonia (average = 2 years) [33], onset of dystonia and psychiatric symptoms at a similar time point also potentially indicates common causative pathways or mechanisms. Similar temporal patterns were not observed across all diagnoses; for example, the risk for SUD was increased in the 2 years leading to dystonia diagnosis and most prominent in the 5 years after dystonia diagnosis. These results suggest that there may be a bidirectional relationship between psychiatric disorders and dystonia, particularly with mood disorders, whereby psychiatric symptoms may form a prodromal component of dystonia, and following dystonia diagnosis there is an increased susceptibility for psychiatric diagnosis. This is also consistent with recent findings that support a susceptibility to psychiatric symptoms, where enrichment of genes linked with psychiatric disorders, including obsessive–compulsive disorder, depression, and schizophrenia, were observed in coexpression modules enriched for dystonia‐associated genes [34].

There was a significantly higher rate of psychiatric medication prescription among those with dystonia compared to controls. Notably, there was 13.1‐fold increase in anxiolytics prescription in the year prior to dystonia diagnosis, particularly in those with cervical dystonia and other dystonia, potentially reflecting the initial burden of a chronic disease diagnosis or again suggesting a potential common mechanistic pathway. However, it is important to note that the majority of prescribed anxiolytic prescriptions were for diazepam (92%), and general practitioners may prescribe diazepam because of its added benefit in the management of dystonia symptoms. We sought to account for this by applying stringent exclusion criteria. This increased risk of psychiatric medication prescription gradually decreased over time, suggesting an absence of continued accumulation of psychiatric symptoms as a secondary consequence of a disabling motor disorder.

Several potential explanations for the association between dystonia and psychiatric disorders may exist. First, a diagnosis of dystonia is delayed, on average, by 2 years from symptom onset. During this time, 50% of patients report a negative personal impact as a result of the delay [33], with the psychological stressors experienced during this period contributing to both diagnosis and prescription of psychiatric medication. There is also considerable overlap in the cortical structures and neurotransmitters implicated in dystonia and psychiatric disorder pathogenesis. Disruption of monoaminergic neurotransmission, notably striatal dopaminergic and serotonergic pathways, has been identified across multiple models of both disorders, with evidence of lower numbers of striatal dopamine receptors, and striatal dopaminergic and midbrain serotonergic binding in AOIFCD coupled with psychiatric symptoms [35, 36, 37].

Although this study represents a step change in the examination of psychopathology in those diagnosed with dystonia, potential limitations exist that are common to all routinely collected data sources. First, it is possible that we included a proportion of cases without dystonia, as we were unable to obtain measures of specificity in our case ascertainment algorithm [1]. We excluded individuals with potential secondary causes of dystonia, which may also have biased the control group. Limited motor treatment data were available for those diagnosed with dystonia, notably use of injectable botulinum toxin, frequently used in the management of the motor symptoms of dystonia but also known to impact psychiatric symptoms in the same patient group [38, 39, 40]. We also had no access to the indication for prescribing, and whether the drug was dispensed or taken, and it is possible some drugs may have been prescribed for dystonic motor symptoms (e.g., diazepam). However, to account for this, we excluded individuals with dystonia who were prescribed benzodiazepines and had no recorded psychiatric diagnoses. Finally, individuals with dystonia may be more likely to seek medical treatment for a psychiatric problem than our control cohort without a neurological diagnosis, and in turn this may explain the increased incidence rates among the cases.

Our study demonstrates that idiopathic dystonia and specific subtypes of dystonia are associated with an increased risk for psychiatric disorders and medication prescription when compared to controls. Higher rates of anxiety and depression, and anxiolytic and antidepressant use, were observed across all dystonia subtypes. Psychiatric disorders generally predated dystonia diagnosis, with incidence rates peaking 12 months prior to dystonia diagnosis. These findings may indicate that psychiatric disorders are either prodromal symptoms or reflect shared underlying pathophysiology. To a lesser extent, increased risk of psychiatric diagnoses and prescriptions were also noted following dystonia diagnosis, implying a potential bidirectional relationship. Further prospective work, using both epidemiological and mechanistic models, is needed to better understand the association between dystonia and psychiatric symptomatology, with particular emphasis on early diagnosis and therapeutic intervention.

## AUTHOR CONTRIBUTIONS

**Grace Bailey:** Conceptualization (equal); data curation (equal); formal analysis (lead); investigation (equal); methodology (equal); project administration (equal); visualization (lead); writing – original draft (lead); writing – review and editing (lead). **Anna Rawlings:** Data curation (lead); resources (lead); validation (lead); writing – review and editing (equal). **Fatemeh Torabi:** Data curation (equal); resources (equal); writing – review and editing (equal). **W Owen Pickrell:** Conceptualization (equal); formal analysis (equal); methodology (equal); supervision (equal); validation (equal); writing – review and editing (equal). **Kathryn J Peall:** Conceptualization (equal); formal analysis (equal); funding acquisition (equal); investigation (equal); methodology (equal); project administration (equal); supervision (equal); visualization (equal); writing – review and editing (equal).

## FUNDING INFORMATION

G.A.B. is funded by a KESS2, European Social Fund, and Cardiff University PhD Studentship. F.T. is funded by Health Data Research UK, which receives its funding from HDR UK (HDR‐9006) funded by the UK Medical Research Council, Engineering and Physical Sciences Research Council, Economic and Social Research Council, Department of Health and Social Care (England), Chief Scientist Office of the Scottish Government Health and Social Care Directorates, Health and Social Care Research and Development Division (Welsh Government), Public Health Agency (Northern Ireland), British Heart Foundation, and Wellcome Trust. W.O.P. is funded by the Brain Repair and Intracranial Neurotherapeutics (BRAIN) Unit Infrastructure Award (grant number: UA05). The BRAIN Unit is funded by Welsh Government through Health and Care Research Wales. K.J.P. is funded by an MRC Clinician–Scientist Fellowship (MR/P008593/1).

## CONFLICT OF INTEREST

The authors report no conflicts of interest.

## ETHICS STATEMENT

In accordance with Health Research Authority guidance, ethical approval is not mandatory for studies using only anonymized data. The SAIL independent Information Governance Review Panel, experts in information governance, and members of the public approved this study (reference: 0768).

## Supporting information


FIGURE S1

FIGURE S2

TABLE S1

TABLE S2

TABLE S3

TABLE S4

TABLE S5

TABLE S6

TABLE S7

TABLE S8

TABLE S9

## Data Availability

Not applicable.
