# Supplementary figures and images for: Longitudinal analysis of the relationship between motor and psychiatric symptoms in idiopathic dystonia
Source: Eur J Neurol. 2022 Sep 11;29(12):3513–27. doi: 10.1111/ene.15530 (PMC9826317; doi:10.1111/ene.15530)

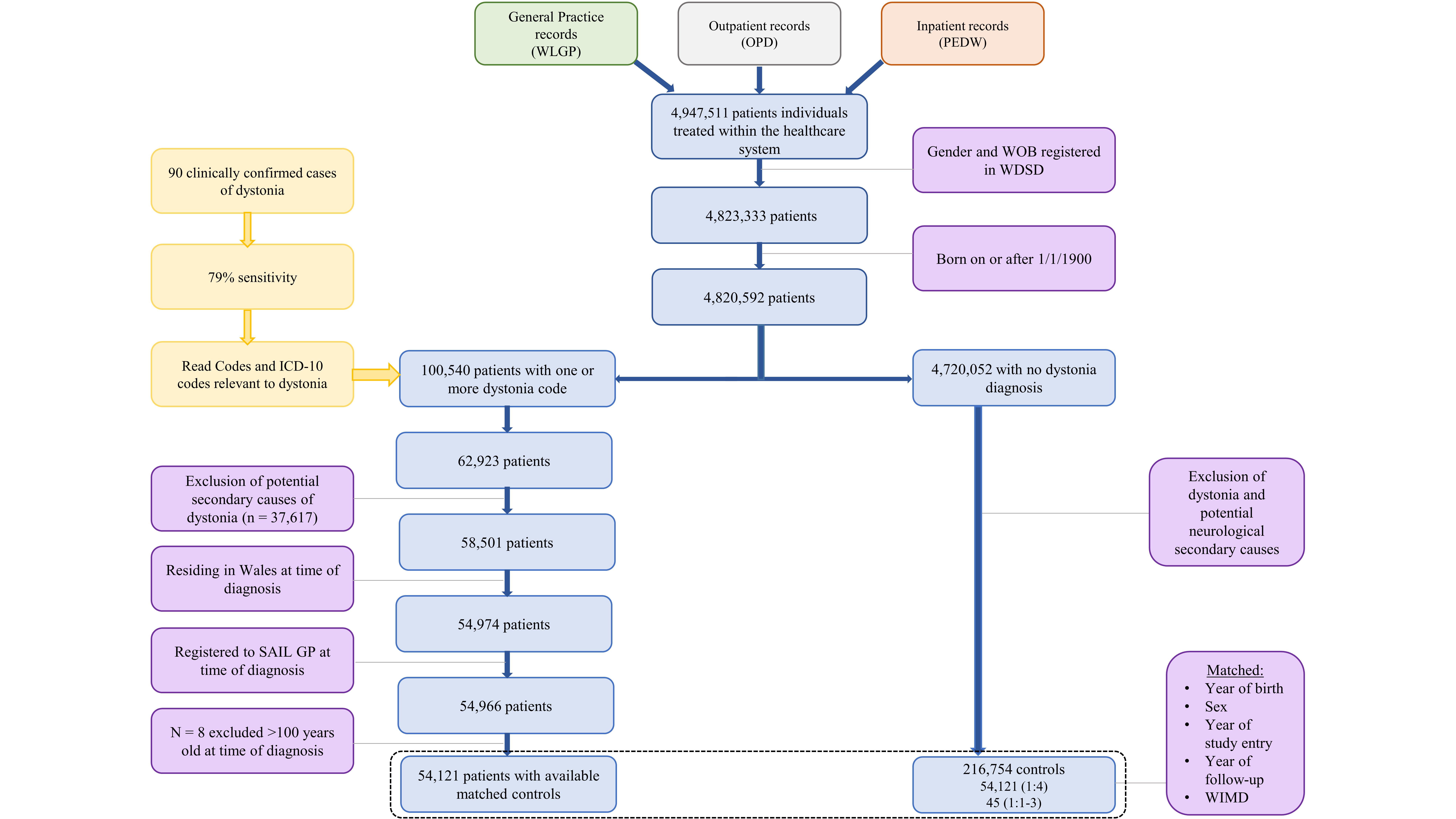

Supplement: Supplementary file 1 — FIGURE S1 [file ENE-29-3513-s011.tif]

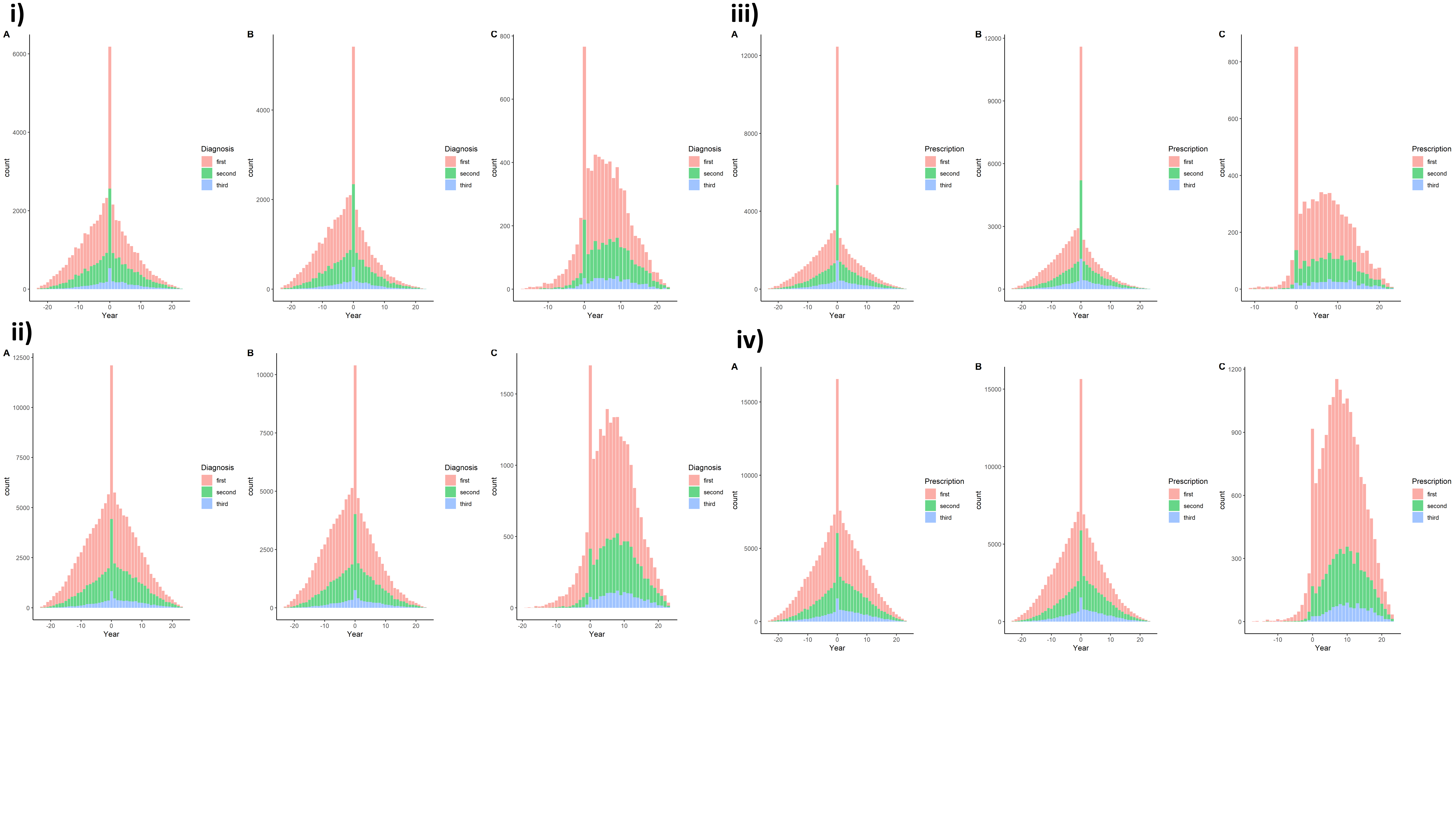

Supplement: Supplementary file 2 — FIGURE S2 [file ENE-29-3513-s002.tif]
