# Supplementary material for: Longitudinal analysis of the relationship between motor and psychiatric symptoms in idiopathic dystonia
Source: Eur J Neurol. 2022 Sep 11;29(12):3513–27. doi: 10.1111/ene.15530 (PMC9826317; doi:10.1111/ene.15530)
Supplement: Supplementary file 3 — TABLE S1 [file ENE-29-3513-s001.docx]

**Supplementary Table 1. Read codes and ICD-10 codes used to identify dystonia cases**

| **Dystonia subtype** | **ICD-10 Code** | **Read Code** | | | **Read Code Description** |
| --- | --- | --- | --- | --- | --- |
| Genetic torsion dystonia | G24.1 |  |  |  |  |
| Idiopathic torsion dystonia |  |  | F136. |  | Idiopathic torsion dystonia |
|  |  |  | F137. |  | Symptomatic torsion dystonia |
|  |  |  | F137y |  | Symptomatic torsion dystonia OS |
|  |  |  | F137z |  | Symptomatic torsion dystonia NOS |
|  |  |  | F138. |  | Fragment of torsion dystonia |
|  |  |  | F138z |  | Torsion dystonia fragment NOS |
| Idiopathic nonfamilial dystonia | G24.2 |  |  |  |  |
| Idiopathic familial dystonia |  |  | F1360 |  | Idiopathic familial dystonia |
| Cervical dystonia | G24.3 |  | F1382 |  | Spasmodic torticollis |
|  |  |  | 16A3. |  | Torticollis - symptom |
|  |  |  | N135. |  | Torticollis unspecified |
|  |  |  | N1350 |  | Intermittent torticollis |
|  |  |  | N135z |  | Torticollis NOS |
| Idiopathic Orofacial dystonia | G24.4 |  |  |  |  |
| Blepharospasm | G24.5 |  | F1380 |  | Blepharospasm |
| Writer’s cramp |  |  | F1383 |  | Organic Writer’s cramp |
| Myoclonic dystonia |  |  | F13B. |  | Myoclonic dystonia |
| Segawa syndrome |  |  | F13C. |  | Segawa syndrome |
| Other | G24.8 |  | Fyu24 |  | [X]Other dystonia |
| Unspecified | G24.9 |  | Fyu2A |  | [X]Dystonia, unspecified |
|  |  |  | F13X. |  | Dystonia, unspecified |
| Tremor |  |  | 1B22. |  | Has a tremor |
