# Supplementary material for: Longitudinal analysis of the relationship between motor and psychiatric symptoms in idiopathic dystonia
Source: Eur J Neurol. 2022 Sep 11;29(12):3513–27. doi: 10.1111/ene.15530 (PMC9826317; doi:10.1111/ene.15530)
Supplement: Supplementary file 5 — TABLE S3 [file ENE-29-3513-s008.docx]

**Supplementary Table 3. Read and ICD-10 Codes used to identify psychiatric disorders**

| **Psychiatric disorder** | **ICD-10 Code** | **Read Code** | | | **Description** |
| --- | --- | --- | --- | --- | --- |
| ADHD |  |  |  |  |  |
|  |  |  | 6A61. |  | ADHD annual review |
|  |  |  | 8BPT. |  | Drug therapy for ADHD |
|  |  |  | 8BPT0 |  | Stimulant drug therapy ADHD |
|  |  |  | 8BPT1 |  | Non-stimulnt drug therapy ADHD |
|  |  |  | 9Ngp. |  | On drug therapy ADHD |
|  |  |  | 9Ngp0 |  | On stimulant drug therapy ADHD |
|  |  |  | 9Ngp1 |  | On non-stimulnt drug ther ADHD |
|  |  |  | 9Ol8. |  | ADHD monitr invitatn 1st lettr |
|  |  |  | 9Ol9. |  | ADHD monitr invitatn 2nd lettr |
|  |  |  | 9OlA. |  | ADHD monitr invitatn 3rd lettr |
|  |  |  | E2E.. |  | Childhood hyperkinetic syndr. |
|  |  |  | E2E0. |  | Child attention deficit disord |
|  |  |  | E2E00 |  | Attention deficit-not hyperact |
|  |  |  | E2E01 |  | Attention deficit +hyperactive |
|  |  |  | E2E0z |  | Child attent.deficit dis.NOS |
|  |  |  | E2E1. |  | Hyperkinesis+development delay |
|  | F901 |  | E2E2. |  | Hyperkinetic conduct disorder |
|  |  |  | E2Ey. |  | Other hyperkinetic manifestat. |
|  |  |  | E2Ez. |  | Hyperkinetic syndrome NOS |
|  | F90 |  | Eu90. |  | [X]Hyperkinetic disorders |
|  | F900 |  | Eu900 |  | [X]Disturbance activity/attntn |
|  |  |  | Eu901 |  | [X]Hyperkinetic conduct disord |
|  |  |  | Eu902 |  | [X]Def atten motor cont percep |
|  | F908 |  | Eu90y |  | [X]Oth hyperkinetic disorders |
|  | F909 |  | Eu90z |  | [X]Hyperkinetic disorder, unsp |
|  |  |  | Eu9y7 |  | [X]Attention deficit disorder |
| Anxiety |  |  |  |  |  |
|  |  |  | 2258. |  | O/E - anxious |
|  |  |  | 2259. |  | O/E - nervous |
|  |  |  | 1B12. |  | Nerves - nervousness |
|  |  |  | 1B13. |  | Anxiousness |
|  |  |  | 1B1V. |  | C/O - panic attack |
|  |  |  | 225J. |  | O/E - panic attack |
|  |  |  | 8G52. |  | Antiphobic therapy |
|  |  |  | 8G94. |  | Anxiety management training |
|  |  |  | 8HHp. |  | Ref guid self-help for anxiety |
|  |  |  | E200. |  | Anxiety states |
|  |  |  | E2000 |  | Anxiety state unspecified |
|  |  |  | E2001 |  | Panic disorder |
|  |  |  | E2002 |  | Generalised anxiety disorder |
|  |  |  | E2003 |  | Anxiety with depression |
|  |  |  | E2004 |  | Chronic anxiety |
|  |  |  | E2005 |  | Recurrent anxiety |
|  |  |  | E200z |  | Anxiety state NOS |
|  | F40 |  | E202. |  | Phobic disorders |
|  |  |  | E2020 |  | Phobia unspecified |
|  |  |  | E2021 |  | Agoraphobia with panic attacks |
|  |  |  | E2022 |  | Agoraphobia - no panic attacks |
|  |  |  | E2023 |  | Social phobia-eating in public |
|  |  |  | E2024 |  | Social phobia-public speaking |
|  |  |  | E2025 |  | Social phobia-public washing |
|  |  |  | E2026 |  | Acrophobia |
|  |  |  | E2027 |  | Animal phobia |
|  |  |  | E2028 |  | Claustrophobia |
|  |  |  | E2029 |  | Fear of crowds |
|  |  |  | E202A |  | Fear of flying |
|  |  |  | E202B |  | Cancer phobia |
|  |  |  | E202C |  | Dental phobia |
|  |  |  | E202D |  | Fear of death |
|  |  |  | E202E |  | Fear of pregnancy |
|  |  |  | E202z |  | Phobic disorder NOS |
|  |  |  | E203. |  | Obsessive-compulsive disorders |
|  |  |  | E2030 |  | Compulsive neurosis |
|  |  |  | E2031 |  | Obsessional neurosis |
|  |  |  | E203z |  | Obsessive-compulsive dis NOS |
|  |  |  | E28z. |  | Acute stress reaction NOS |
|  |  |  | E2920 |  | Separation anxiety disorder |
|  |  |  | E2D0. |  | Anxiety/fear child/adoles.dis. |
|  |  |  | E2D00 |  | Child/adolesc.overanxious.dis. |
|  |  |  | E2D01 |  | Child/adolesc.fearfulness dis. |
|  |  |  | E2D0z |  | Anxiety/fear child/adolesc.NOS |
|  |  |  | Eu40. |  | [X]Phobic anxiety disorders |
|  | F400 |  | Eu400 |  | [X]Agoraphobia |
|  | F401 |  | Eu401 |  | [X]Social phobias |
|  | F402 |  | Eu402 |  | [X]Specific (isolated) phobia |
|  |  |  | Eu403 |  | [X]Needle phobia |
|  | F408 |  | Eu40y |  | [X]Other phobic anxiety disord |
|  | F409 |  | Eu40z |  | [X]Phobic anxiety disordr unsp |
|  | F41 |  | Eu41. |  | [X]Other anxiety disorders |
|  | F410 |  | Eu410 |  | [X]Panic episodic paroxysm anx |
|  | F411 |  | Eu411 |  | [X]Generalized anxiety disord |
|  | F412 |  | Eu412 |  | [X]Mixed anxiety/depressve dis |
|  | F413 |  | Eu413 |  | [X]Other mixed anxiety disord |
|  | F418 |  | Eu41y |  | [X]Other specif anxiety disord |
|  | F419 |  | Eu41z |  | [X]Anxiety disord unspecified |
|  | F42 |  | Eu42. |  | [X]Obsessive - compulsive dis |
|  | F420 |  | Eu420 |  | [X]Predom obsessional thoughts |
|  | F421 |  | Eu421 |  | [X]Predom compuls acts/ritual |
|  | F422 |  | Eu422 |  | [X]Mixed obsess thoughts/acts |
|  | F428 |  | Eu42y |  | [X]Oth obsessve-compulsve dis |
|  | F429 |  | Eu42z |  | [X]Obsessve-complsve dis unsp |
|  | F930 |  | Eu930 |  | [X]Separ anxiety dis childhood |
|  | F931 |  | Eu931 |  | [X]Phobic anxiet dis childhood |
|  | F932 |  | Eu932 |  | [X]Social anx dis childhood |
|  |  |  | R2y2. |  | [D]Nervousness |
| Autism |  |  |  |  |  |
|  |  |  | 1J9.. |  | Suspected autism |
|  |  |  | E140. |  | Infantile autism |
|  |  |  | E1400 |  | Infantile autism - active |
|  |  |  | E1401 |  | Infantile autism - residual |
|  |  |  | E140z |  | Infantile autism NOS |
|  |  |  | Eu84. |  | [X]Pervasive development dis |
|  | F840 |  | Eu840 |  | [X]Childhood autism |
|  | F841 |  | Eu841 |  | [X]Atypical autism |
|  | F842 |  | Eu842 |  | [X]Rett's syndrome |
|  | F843 |  | Eu843 |  | [X]Oth child disintegrat dis |
|  | F844 |  | Eu844 |  | [X]Overact+retard+stereotyp mv |
|  | F845 |  | Eu845 |  | [X]Asperger's syndrome |
|  |  |  | Eu846 |  | Pathological demand avoidance |
|  | F848 |  | Eu84y |  | [X]Oth pervasive develop dis |
|  | F849 |  | Eu84z |  | [X]Pervasve develop dis unsp |
| Conduct disorder |  |  |  |  |  |
|  |  |  | E2C.. |  | Disturbance of conduct NEC |
|  |  |  | E2C0. |  | Aggress.unsocial conduct dis. |
|  |  |  | E2C0z |  | Aggressive unsocial disord.NOS |
|  |  |  | E2C1. |  | Nonaggr.unsocial conduct dis. |
|  |  |  | E2C10 |  | Unsocial childhood truancy |
|  |  |  | E2C1z |  | Nonaggr.unsocial cond.dis.NOS |
|  |  |  | E2C2. |  | Socialised conduct disorder |
|  |  |  | E2C20 |  | Socialised childhood truancy |
|  |  |  | E2C2z |  | Socialised conduct disord. NOS |
|  |  |  | E2C4. |  | Mixed conduct/emotion disturb. |
|  |  |  | E2C4z |  | Mixed conduct/emotion dist.NOS |
|  |  |  | E2Cy. |  | Other conduct disturbances |
|  |  |  | E2Cyz |  | Other conduct disturbances NOS |
|  |  |  | E2Cz. |  | Disturbance of conduct unspec. |
|  |  |  | E2Czz |  | Conduct disturbance NOS |
|  |  |  | E2Dy0 |  | Childhood oppositional disord. |
|  |  |  | E2E2. |  | Hyperkinetic conduct disorder |
|  | F901 |  | Eu901 |  | [X]Hyperkinetic conduct disord |
|  | F91 |  | Eu91. |  | [X]Conduct disorders |
|  | F910 |  | Eu910 |  | [X]Conduct dis family context |
|  | F911 |  | Eu911 |  | [X]Unsocialized conduct disord |
|  | F912 |  | Eu912 |  | [X]Socialized conduct disorder |
|  | F913 |  | Eu913 |  | [X]Oppositional defiant disord |
|  | F918 |  | Eu91y |  | [X]Other conduct disorders |
|  | F919 |  | Eu91z |  | [X]Conduct disorder, unspecif |
|  | F92 |  | Eu92. |  | [X]Mixed dis conduct/emotion |
|  | F920 |  | Eu920 |  | [X]Depressive conduct disorder |
|  | F928 |  | Eu92y |  | [X]Oth mix disord conduct/emot |
|  | F929 |  | Eu92z |  | [X]Mixed dis conduct/emot unsp |
| Depression |  |  |  |  |  |
|  |  |  | 2257. |  | O/E - depressed |
|  |  |  | 1B17. |  | Depressed |
|  |  |  | 1B1U. |  | Symptoms of depression |
|  |  |  | 1BP.. |  | Loss of interest |
|  |  |  | 1BP0. |  | Loss of inter prev enjoy activ |
|  |  |  | 1BQ.. |  | Loss of capacity for enjoyment |
|  |  |  | 1BT.. |  | Depressed mood |
|  |  |  | 1BU.. |  | Loss of hope for the future |
|  |  |  | E112. |  | Single major depressive episod |
|  |  |  | E1120 |  | Single major depression-unspec |
|  |  |  | E1121 |  | Single major depression-mild |
|  |  |  | E1122 |  | Single major depress.-moderate |
|  |  |  | E1123 |  | Single major depression-severe |
|  |  |  | E1125 |  | Single maj.depres.-part remiss |
|  |  |  | E1126 |  | Single maj.depres.-full remiss |
|  |  |  | E112z |  | Single major depression NOS |
|  |  |  | E113. |  | Recurrent major depressive epi |
|  |  |  | E1130 |  | Recurr.major depression-unspec |
|  |  |  | E1131 |  | Recurr.major depression-mild |
|  |  |  | E1132 |  | Recurr.major depress.-moderate |
|  |  |  | E1133 |  | Recurr.major depression-severe |
|  |  |  | E1135 |  | Recurr.maj.depres.-part remiss |
|  |  |  | E1136 |  | Recurr.maj.depres.-full remiss |
|  |  |  | E1137 |  | Recurrent depression |
|  |  |  | E113z |  | Recurr. major depression NOS |
|  |  |  | E118. |  | Seasonal affective disorder |
|  |  |  | E135. |  | Agitated depression |
|  |  |  | E2003 |  | Anxiety with depression |
|  |  |  | E204. |  | Neurotic (reactive) depression |
|  |  |  | E291. |  | Prolonged depressive reaction |
|  |  |  | E2B.. |  | Depressive disorder NEC |
|  |  |  | E2B0. |  | Postviral depression |
|  |  |  | E2B1. |  | Chronic depression |
|  | F32 |  | Eu32. |  | [X]Depressive episode |
|  | F320 |  | Eu320 |  | [X]Mild depressive episode |
|  | F321 |  | Eu321 |  | [X]Moderate depressve episode |
|  | F322 |  | Eu322 |  | [X]Severe depressiv no psychot |
|  | F328 |  | Eu324 |  | [X]Mild depression |
|  | F329 |  | Eu325 |  | [X]Major depression, mild |
|  | F33 |  | Eu326 |  | [X]Major depression, moder sev |
|  | F330 |  | Eu327 |  | [X]Maj dep, sev wthout psy sym |
|  | F331 |  | Eu32B |  | [X]Antenatal depression |
|  | F332 |  | Eu32y |  | [X]Other depressive episodes |
|  | F334 |  | Eu32z |  | [X]Depressive episode, unspecf |
|  | F338 |  | Eu33. |  | [X]Recurrent depressive disord |
|  | F339 |  | Eu330 |  | [X]Recurr depress current mild |
|  | F341 |  | Eu331 |  | [X]Recurr depress current mod |
|  | F412 |  | Eu332 |  | [X]Recurr dep now sever no psy |
|  | F32 |  | Eu334 |  | [X]Recurr depress in remission |
|  | F320 |  | Eu33y |  | [X]Oth recurr depressve disord |
|  | F321 |  | Eu33z |  | [X]Recurrent depress dis unsp |
|  | F322 |  | Eu341 |  | [X]Dysthymia |
|  | F328 |  | Eu412 |  | [X]Mixed anxiety/depressve dis |
| Eating disorder |  |  |  |  |  |
|  |  |  | 1467. |  | H/O: anorexia nervosa |
|  |  |  | 1612. |  | Appetite loss - anorexia |
|  |  |  | 1FF.. |  | Binge eating |
|  |  |  | 8HTN. |  | Refer eating disorders clinic |
|  |  |  | 9Nk9. |  | Seen in eating disorder clinic |
|  |  |  | E271. |  | Anorexia nervosa |
|  |  |  | E275. |  | Other non-organic eating dis. |
|  |  |  | E2750 |  | Non-organic eating dis.unspec. |
|  |  |  | E2751 |  | Bulimia (non-org. overeating) |
|  |  |  | E275y |  | Non-organic eating disord. OS |
|  |  |  | E275z |  | Non-organic eating disord. NOS |
|  | F50 |  | Eu50. |  | [X]Eating disorders |
|  | F500 |  | Eu500 |  | [X]Anorexia nervosa |
|  | F501 |  | Eu501 |  | [X]Atypical anorexia nervosa |
|  | F502 |  | Eu502 |  | [X]Bulimia nervosa |
|  | F503 |  | Eu503 |  | [X]Atypical bulimia nervosa |
|  |  |  | Eu504 |  | [X]Overeat + oth psychol dist |
|  |  |  | Eu50y |  | [X]Other eating disorders |
|  | F509 |  | Eu50z |  | [X]Eating disorder unspecified |
|  |  |  | R030. |  | [D]Anorexia |
|  |  |  | R030z |  | [D]Anorexia NOS |
|  |  |  | R0360 |  | [D]Excessive eating |
|  | F982 |  |  |  | Feeding disorder of infancy and childhood |
| Learning difficulties |  |  |  |  |  |
|  |  |  | 2B5.. |  | O/E - symbolic dysfunction |
|  |  |  | 2B55. |  | O/E - dyslexia |
|  |  |  | 8E23. |  | Dyslexia training |
|  |  |  | E2F02 |  | Developmental dyslexia |
|  | F80 |  | Eu80. |  | [X]Spec develop dis speech/lan |
|  | F800 |  | Eu800 |  | [X]Specif speech articulat dis |
|  | F801 |  | Eu801 |  | [X]Expressive language disordr |
|  | F809 |  | Eu80z |  | [X]Dev disord speech/lang unsp |
|  | F81 |  | Eu81. |  | [X]Specif scholastic devel dis |
|  | F810 |  | Eu810 |  | [X]Specific reading disorder |
|  | F811 |  | Eu811 |  | [X]Specific spelling disorder |
|  | F812 |  | Eu812 |  | [X]Specific disord arithmetic |
|  | F813 |  | Eu813 |  | [X]Mixed scholastic skill dis |
|  | F818 |  | Eu81y |  | [X]Oth dis scholastic skills |
|  | F819 |  | Eu81z |  | [X]Dev dis scholas skills unsp |
|  | F82 |  | Eu82. |  | [X]Spec devel disor motor func |
|  | F83 |  | Eu83. |  | [X]Mix specific develop disord |
|  |  |  | R046. |  | [D]Other symbolic dysfunction |
|  |  |  | R0463 |  | [D]Dyslexia |
|  | R48 |  |  |  | Dyslexia and other symbolic dysfunctions, not elsewhere classified |
|  | R480 |  |  |  | Dyslexia and alexia |
| Severe mental illness |  |  |  |  |  |
|  |  |  | 1464. |  | H/O: schizophrenia |
|  |  |  | 146D. |  | H/O: manic depressive disorder |
|  |  |  | 146H. |  | H/O: psychosis |
|  |  |  | 8HHs. |  | Ref psychosis early inter ser |
|  |  |  | 9H8.. |  | On severe mental illnes regist |
|  |  |  | E10.. |  | Schizophrenic disorders |
|  |  |  | E100. |  | Simple schizophrenia |
|  |  |  | E1000 |  | Schizophrenia - unspecified |
|  |  |  | E1001 |  | Schizophrenia - subchronic |
|  |  |  | E1002 |  | Schizophrenia - chronic type |
|  |  |  | E1003 |  | Schizophrenia-subchr.+acute ex |
|  |  |  | E1004 |  | Schizophrenia-chr.+acute exac. |
|  |  |  | E1005 |  | Schizophrenia in remission |
|  |  |  | E100z |  | Simple schizophrenia NOS |
|  |  |  | E101. |  | Hebephrenic schizophrenia |
|  |  |  | E1010 |  | Hebephrenia - unspecified |
|  |  |  | E1011 |  | Hebephrenia - subchronic |
|  |  |  | E1012 |  | Hebephrenia - chronic |
|  |  |  | E1013 |  | Hebephrenia-subchr.+acute exac |
|  |  |  | E1014 |  | Hebephrenia-chronic+acute exac |
|  |  |  | E1015 |  | Hebephrenia - in remission |
|  |  |  | E101z |  | Hebephrenic schizophrenia NOS |
|  |  |  | E102. |  | Catatonic schizophrenia |
|  |  |  | E1020 |  | Catatonia - unspecified |
|  |  |  | E1021 |  | Catatonia - subchronic |
|  |  |  | E1022 |  | Catatonia - chronic |
|  |  |  | E1023 |  | Catatonia-subchr.+acute exac. |
|  |  |  | E1024 |  | Catatonia-chronic+acute exacer |
|  |  |  | E1025 |  | Catatonia - in remission |
|  |  |  | E102z |  | Catatonic schizophrenia NOS |
|  |  |  | E103. |  | Paranoid schizophrenia |
|  |  |  | E1030 |  | Paranoid schizo.- unspecified |
|  |  |  | E1031 |  | Paranoid schizo.- subchronic |
|  |  |  | E1032 |  | Paranoid schizo.- chronic |
|  |  |  | E1033 |  | Paranoid schizo.-subchr.+ac ex |
|  |  |  | E1034 |  | Paranoid schizo.-chr.+acute ex |
|  |  |  | E1035 |  | Paranoid schizo.-in remission |
|  |  |  | E103z |  | Paranoid schizophrenia NOS |
|  |  |  | E104. |  | Acute schizophrenic episode |
|  |  |  | E105. |  | Latent schizophrenia |
|  |  |  | E1050 |  | Latent schizo.- unspecified |
|  |  |  | E1051 |  | Latent schizo.- subchronic |
|  |  |  | E1052 |  | Latent schizo.- chronic |
|  |  |  | E1053 |  | Latent schizo.-subchr.+ac.exac |
|  |  |  | E1054 |  | Latent schizo.-chr.+acute exac |
|  |  |  | E1055 |  | Latent schizo.- in remission |
|  |  |  | E105z |  | Latent schizophrenia NOS |
|  |  |  | E106. |  | Residual schizophrenia |
|  |  |  | E107. |  | Schizo-affective schizophrenia |
|  |  |  | E1070 |  | Schizo-affective - unspecified |
|  |  |  | E1071 |  | Schizo-affective - subchronic |
|  |  |  | E1072 |  | Schizo-affective - chronic |
|  |  |  | E1073 |  | Schizo-affective-subchr.+ac ex |
|  |  |  | E1074 |  | Schizo-affective-chr.+acute ex |
|  |  |  | E1075 |  | Schizo-affective-in remission |
|  |  |  | E107z |  | Schizo-affective schizophr.NOS |
|  |  |  | E10y. |  | Other schizophrenia |
|  |  |  | E10y0 |  | Atypical schizophrenia |
|  |  |  | E10y1 |  | Coenesthopathic schizophrenia |
|  |  |  | E10yz |  | Other schizophrenia NOS |
|  |  |  | E10z. |  | Schizophrenia NOS |
|  |  |  | E11.. |  | Affective psychoses |
|  |  |  | E110. |  | Manic disorder, single episode |
|  |  |  | E1100 |  | Single manic episode-unspecif |
|  |  |  | E1101 |  | Single manic episode-mild |
|  |  |  | E1102 |  | Single manic episode-moderate |
|  |  |  | E1103 |  | Single manic episode-severe |
|  |  |  | E1104 |  | Single manic epis.-severe+psyc |
|  |  |  | E1105 |  | Single manic epis-part remiss. |
|  |  |  | E1106 |  | Single manic epis-full remiss. |
|  |  |  | E110z |  | Manic disorder-single epis.NOS |
|  |  |  | E111. |  | Recurrent manic episodes |
|  |  |  | E1110 |  | Recurrent manic episode-unspec |
|  |  |  | E1111 |  | Recurrent manic episode-mild |
|  |  |  | E1112 |  | Recurrent manic epis.-moderate |
|  |  |  | E1113 |  | Recurrent manic epis.-severe |
|  |  |  | E1114 |  | Recurr.manic epis.-severe+psyc |
|  |  |  | E1115 |  | Recur.manic epis.-part remiss. |
|  |  |  | E1116 |  | Recur.manic epis.-full remiss. |
|  |  |  | E111z |  | Recurrent manic episode NOS |
|  |  |  | E1124 |  | Single maj.depress.severe+psyc |
|  |  |  | E1134 |  | Recurr.maj.depres.-severe+psyc |
|  |  |  | E114. |  | Bipolar affective - now manic |
|  |  |  | E1140 |  | Manic bipolar affective-unspec |
|  |  |  | E1141 |  | Manic bipolar affective-mild |
|  |  |  | E1142 |  | Manic bipolar affect.-moderate |
|  |  |  | E1143 |  | Manic bipolar affect.-severe |
|  |  |  | E1144 |  | Manic bipol.affect.severe+psyc |
|  |  |  | E1145 |  | Manic bipol.affect.part remiss |
|  |  |  | E1146 |  | Manic bipol.affect.full remiss |
|  |  |  | E114z |  | Manic bipolar affective NOS |
|  |  |  | E115. |  | Bipolar affective - now depres |
|  |  |  | E1150 |  | Depressed bipolar affect.-unsp |
|  |  |  | E1151 |  | Depress.bipolar affect.-mild |
|  |  |  | E1152 |  | Depr.bipolar affect.-moderate |
|  |  |  | E1153 |  | Depr.bipolar affect.-severe |
|  |  |  | E1154 |  | Depr.bipol.affect.-severe+psyc |
|  |  |  | E1155 |  | Depr.bipol.affect.-part remiss |
|  |  |  | E1156 |  | Depr.bipol.affect.-full remiss |
|  |  |  | E115z |  | Depressed bipolar affect. NOS |
|  |  |  | E116. |  | Mixed bipolar affective disord |
|  |  |  | E1160 |  | Mixed bipolar affective-unspec |
|  |  |  | E1161 |  | Mixed bipolar affective-mild |
|  |  |  | E1162 |  | Mixed bipolar affect.-moderate |
|  |  |  | E1163 |  | Mixed bipolar affect.-severe |
|  |  |  | E1164 |  | Mixed bipol.affect.severe+psyc |
|  |  |  | E1165 |  | Mixed bipol.affect.part remiss |
|  |  |  | E1166 |  | Mixed bipol.affect.full remiss |
|  |  |  | E116z |  | Mixed bipolar affective NOS |
|  |  |  | E117. |  | Unspec bipolar affect disord |
|  |  |  | E1170 |  | Unspecified bipolar affective |
|  |  |  | E1171 |  | Unsp.bipolar affective-mild |
|  |  |  | E1172 |  | Unsp.bipolar affect.-moderate |
|  |  |  | E1173 |  | Unsp.bipolar affect.-severe |
|  |  |  | E1174 |  | Unsp.bipol.affect.-severe+psyc |
|  |  |  | E1175 |  | Unsp.bipol.affect.-part remiss |
|  |  |  | E1176 |  | Unsp.bipol.affect.-full remiss |
|  |  |  | E117z |  | Unspecif.bipolar affective NOS |
|  |  |  | E11y. |  | Other manic-depressive psychos |
|  |  |  | E11y0 |  | Unspec manic-depressive psycho |
|  |  |  | E11y1 |  | Atypical manic disorder |
|  |  |  | E11y2 |  | Atypical depressive disorder |
|  |  |  | E11y3 |  | Other mixed manic-depres psych |
|  |  |  | E11yz |  | Other manic-depress.psych.NOS |
|  |  |  | E11z. |  | Other/unsp.affective psychoses |
|  |  |  | E11z0 |  | Unspecif.affective psych.NOS |
|  |  |  | E11zz |  | Other affective psychosis NOS |
|  |  |  | E12.. |  | Paranoid states |
|  |  |  | E120. |  | Simple paranoid state |
|  |  |  | E121. |  | Chronic paranoid psychosis |
|  |  |  | E122. |  | Paraphrenia |
|  |  |  | E123. |  | Shared paranoid disorder |
|  |  |  | E12y. |  | Other paranoid states |
|  |  |  | E12y0 |  | Paranoia querulans |
|  |  |  | E12yz |  | Other paranoid states NOS |
|  |  |  | E12z. |  | Paranoid psychosis NOS |
|  |  |  | E13.. |  | Other nonorganic psychoses |
|  |  |  | E130. |  | Reactive depressive psychosis |
|  |  |  | E131. |  | Acute hysterical psychosis |
|  |  |  | E132. |  | Reactive confusion |
|  |  |  | E133. |  | Acute paranoid reaction |
|  |  |  | E134. |  | Psychogenic paranoid psychosis |
|  |  |  | E13y. |  | Other reactive psychoses |
|  |  |  | E13y0 |  | Psychogenic stupor |
|  |  |  | E13y1 |  | Brief reactive psychosis |
|  |  |  | E13yz |  | Other reactive psychoses NOS |
|  |  |  | E13z. |  | Nonorganic psychosis NOS |
|  |  |  | E1y.. |  | Non-organic psychoses OS |
|  |  |  | E1z.. |  | Non-organic psychosis NOS |
|  |  |  | E2122 |  | Schizotypal personality |
|  |  |  | Eu2.. |  | [X]Schizoph,schizotyp,delusion |
|  | F20 |  | Eu20. |  | [X]Schizophrenia |
|  | F200 |  | Eu200 |  | [X]Paranoid schizophrenia |
|  | F201 |  | Eu201 |  | [X]Hebephrenic schizophrenia |
|  | F202 |  | Eu202 |  | [X]Catatonic schizophrenia |
|  | F203 |  | Eu203 |  | [X]Undifferentiated schizophrn |
|  | F204 |  | Eu204 |  | [X]Post-schizophrenic depressn |
|  | F205 |  | Eu205 |  | [X]Residual schizophrenia |
|  | F206 |  | Eu206 |  | [X]Simple schizophrenia |
|  | F208 |  | Eu20y |  | [X]Other schizophrenia |
|  | F209 |  | Eu20z |  | [X]Schizophrenia, unspecified |
|  | F21, F21X |  | Eu21. |  | [X]Schizotypal disorder |
|  | F22 |  | Eu22. |  | [X]Persistent delusional dis |
|  | F220 |  | Eu220 |  | [X]Delusional disorder |
|  |  |  | Eu221 |  | [X]Delusion misidentificat syn |
|  |  |  | Eu222 |  | [X]Cotard syndrome |
|  |  |  | Eu223 |  | [X]Paranoid state in remission |
|  | F228 |  | Eu22y |  | [X]Oth persistent delusion dis |
|  | F229 |  | Eu22z |  | [X]Persist delusion dis, unsp |
|  | F23 |  | Eu23. |  | [X]Acute/transient psychot dis |
|  | F230 |  | Eu230 |  | [X]Ac polymorph psych,no schiz |
|  | F231 |  | Eu231 |  | [X]Ac polymorph psych + schiz |
|  | F232 |  | Eu232 |  | [X]Ac schizoph-like psych dis |
|  | F233 |  | Eu233 |  | [X]Oth ac predom delus psy dis |
|  | F238 |  | Eu23y |  | [X]Oth ac and trans psych dis |
|  | F239 |  | Eu23z |  | [X]Ac and trans psych dis unsp |
|  | F24, F24X |  | Eu24. |  | [X]Induced delusional disorder |
|  | F25 |  | Eu25. |  | [X]Schizoaffective disorders |
|  | F250 |  | Eu250 |  | [X]Schizaffect dis manic type |
|  | F251 |  | Eu251 |  | [X]Schzaffctve dis depres type |
|  | F252 |  | Eu252 |  | [X]Schizaffctve dis mixed type |
|  | F258 |  | Eu25y |  | [X]Ot schizoaffective disorder |
|  | F259 |  | Eu25z |  | [X]Schizoaffective disord unsp |
|  |  |  | Eu26. |  | [X]Nonorgan psychos in remiss |
|  | F28, F28X |  | Eu2y. |  | [X]Ot nonorganic psychotic dis |
|  | F29, F29X |  | Eu2z. |  | [X]Unspec nonorganic psychosis |
|  | F30 |  | Eu30. |  | [X]Manic episode |
|  | F300 |  | Eu300 |  | [X]Hypomania |
|  | F301 |  | Eu301 |  | [X]Mania without psychotic sym |
|  | F302 |  | Eu302 |  | [X]Mania with psychotic sympts |
|  | F308 |  | Eu30y |  | [X]Other manic episodes |
|  | F309 |  | Eu30z |  | [X]Manic episode, unspecified |
|  | F31 |  | Eu31. |  | [X]Bipolar affective disorder |
|  | F310 |  | Eu310 |  | [X]Bipol affec current hypoman |
|  | F311 |  | Eu311 |  | [X]Bipol aff, manic no psychos |
|  | F312 |  | Eu312 |  | [X]Bipol affect manic+psychos |
|  | F313 |  | Eu313 |  | [X]Bipol aff mild/mod depress |
|  | F314 |  | Eu314 |  | [X]Bipol AD,cur sev dep,no psy |
|  | F315 |  | Eu315 |  | [X]Bipol aff sev depress/psych |
|  | F316 |  | Eu316 |  | [X]Bipol affective dis, mixed |
|  | F317 |  | Eu317 |  | [X]Bipol affect dis remission |
|  |  |  | Eu318 |  | [X]Bipol affect disord type I |
|  |  |  | Eu319 |  | [X]Bipol affect disord type II |
|  | F318 |  | Eu31y |  | [X]Oth bipolar affectve disord |
|  | F319 |  | Eu31z |  | [X]Bipolar affectve dis unsp |
|  | F323 |  | Eu323 |  | [X]Severe depressive + psychot |
|  |  |  | Eu328 |  | [X]Maj dep, sev with psyc symp |
|  |  |  | Eu329 |  | [X]Sin ma dep ep sev ps ps rem |
|  |  |  | Eu32A |  | [X]Rec ma dep ep sev ps ps rem |
|  | F333 |  | Eu333 |  | [X]Recurr dep now sever+psych |
|  |  |  | ZV110 |  | [V]PH - Schizophrenia |
|  | F38 |  |  |  | Other mood [affective] disorders |
|  | F380 |  |  |  | Other single mood [affective] disorders |
|  | F381 |  |  |  | Other recurrent mood [affective] disorders |
|  | F388 |  |  |  | Other specified mood [affective] disorders |
|  | F39, F39X |  |  |  | Unspecified mood [affective] disorder |
| Substance use disorder |  |  |  |  |  |
|  |  |  | E01.. |  | Alcoholic psychoses |
|  |  |  | E010. |  | Alcohol withdrawal delirium |
|  |  |  | E011. |  | Alcohol amnestic syndrome |
|  |  |  | E0110 |  | Korsakovs alcoholic psychosis |
|  |  |  | E0111 |  | Korsakov + peripheral neuritis |
|  |  |  | E0112 |  | Wernicke-Korsakov syndrome |
|  |  |  | E011z |  | Alcohol amnestic syndrome NOS |
|  |  |  | E012. |  | Other alcoholic dementia |
|  |  |  | E0120 |  | Chronic alcoholic brain syndr. |
|  |  |  | E013. |  | Alcohol withdrawal hallucinos. |
|  |  |  | E014. |  | Pathological alcohol intoxic. |
|  |  |  | E015. |  | Alcoholic paranoia |
|  |  |  | E01y. |  | Other alcoholic psychosis |
|  |  |  | E01y0 |  | Alcohol withdrawal syndrome |
|  |  |  | E01yz |  | Other alcoholic psychosis NOS |
|  |  |  | E01z. |  | Alcoholic psychosis NOS |
|  |  |  | E02.. |  | Drug psychoses |
|  |  |  | E020. |  | Drug withdrawal syndrome |
|  |  |  | E021. |  | Drug-induced paranoia/hallucin |
|  |  |  | E0210 |  | Drug-induced paranoid state |
|  |  |  | E0211 |  | Drug-induced hallucinosis |
|  |  |  | E021z |  | Drug-induc.paranoia/halluc NOS |
|  |  |  | E022. |  | Pathological drug intoxication |
|  |  |  | E02y. |  | Other drug psychoses |
|  |  |  | E02y0 |  | Drug-induced delirium |
|  |  |  | E02y1 |  | Drug-induced dementia |
|  |  |  | E02y2 |  | Drug-induced amnestic syndrome |
|  |  |  | E02y3 |  | Drug-induced depressive state |
|  |  |  | E02y4 |  | Drug-induced personality dis. |
|  |  |  | E02yz |  | Other drug psychoses NOS |
|  |  |  | E02z. |  | Drug psychosis NOS |
|  |  |  | Eu1.. |  | [X]Mental dis, psychoact subst |
|  | F10, F100-109 |  | Eu10. |  | [X]Mental dis due to alcohol |
|  |  |  | Eu100 |  | [X]Acute alcohol intoxication |
|  |  |  | Eu101 |  | [X]Harmful use of alcohol |
|  |  |  | Eu102 |  | [X]Alcohol dependence syndrome |
|  |  |  | Eu103 |  | [X]Alcohol withdrawal state |
|  |  |  | Eu104 |  | [X]Alcohol withdrawal delirium |
|  |  |  | Eu105 |  | [X]Psychot dis due to alcohol |
|  |  |  | Eu106 |  | [X]Amnesic synd due to alcohol |
|  |  |  | Eu107 |  | [X]Resid psychotic due alcohol |
|  |  |  | Eu108 |  | [X]Alcohol withdraw-induc seiz |
|  |  |  | Eu10y |  | [X]Ot ment/beh dis due alcohol |
|  |  |  | Eu10z |  | [X]Uns ment/beh dis due alcohl |
|  | F11, F110-F119 |  | Eu11. |  | [X]Mental dis due to opioids |
|  |  |  | Eu110 |  | [X]Acute opioid intoxication |
|  |  |  | Eu111 |  | [X]Harmful use of opioids |
|  |  |  | Eu112 |  | [X]Opioid dependence syndrome |
|  |  |  | Eu113 |  | [X]Opioid withthdrawal state |
|  |  |  | Eu114 |  | [X]Opioid withdrawal delirium |
|  |  |  | Eu115 |  | [X]Psychot dis due to opioids |
|  |  |  | Eu116 |  | [X]Amnesic synd due to opioids |
|  |  |  | Eu117 |  | [X]Resid psychotic due opioid |
|  |  |  | Eu11y |  | [X]Oth ment/beh dis due opioid |
|  |  |  | Eu11z |  | [X]Uns ment/beh dis due opioid |
|  | F12, F120-F129 |  | Eu12. |  | [X]Mental dis due cannabinoids |
|  |  |  | Eu120 |  | [X]Acute cannabis intoxication |
|  |  |  | Eu121 |  | [X]Harmful use of cannabis |
|  |  |  | Eu122 |  | [X]Cannabis dependence syndrom |
|  |  |  | Eu123 |  | [X]Cannabis withdrawal state |
|  |  |  | Eu124 |  | [X]Cannabis withdrawl delirium |
|  |  |  | Eu125 |  | [X]Psychot dis due to cannabis |
|  |  |  | Eu126 |  | [X]Amnesic synd due cannabis |
|  |  |  | Eu127 |  | [X]Resid psychot due cannabis |
|  |  |  | Eu12y |  | [X]Oth ment/beh dis cannabinds |
|  |  |  | Eu12z |  | [X]Unsp mnt/beh dis cannabinds |
|  | F13, F130-F139 |  | Eu13. |  | [X]Mental dis due sedat/hypnot |
|  |  |  | Eu130 |  | [X]Acute sedat/hypnotic intox |
|  |  |  | Eu131 |  | [X]Harmful use sedat/hypnotic |
|  |  |  | Eu132 |  | [X]Sedat/hypnotic depend syndr |
|  |  |  | Eu133 |  | [X]Sedat/hypnot withdraw state |
|  |  |  | Eu134 |  | [X]Sed/hypn withdraw delirium |
|  |  |  | Eu135 |  | [X]Psychot dis due sedat/hypn |
|  |  |  | Eu136 |  | [X]Amnesic synd due sedat/hypn |
|  |  |  | Eu137 |  | [X]Resid psychot due sed/hypn |
|  |  |  | Eu13y |  | [X]Oth ment/beh dis sed/hypnot |
|  |  |  | Eu13z |  | [X]Uns ment/beh dis sed/hypnot |
|  | F14, F140-F149 |  | Eu14. |  | [X]Mental dis due use cocaine |
|  |  |  | Eu140 |  | [X]Acute cocaine intoxication |
|  |  |  | Eu141 |  | [X]Harmful use of cocaine |
|  |  |  | Eu142 |  | [X]Cocaine dependence syndrome |
|  |  |  | Eu143 |  | [X]Cocaine withdrawal state |
|  |  |  | Eu144 |  | [X]Cocaine withdrawal delirium |
|  |  |  | Eu145 |  | [X]Psychot dis due to cocaine |
|  |  |  | Eu146 |  | [X]Amnesic synd due to cocaine |
|  |  |  | Eu147 |  | [X]Resid psychot due cocaine |
|  |  |  | Eu14y |  | [X]Ot ment/beh dis due cocaine |
|  |  |  | Eu14z |  | [X]Uns ment/bh dis due cocaine |
|  | F15, F150-F159 |  | Eu15. |  | [X]Ment dis oth stimul/caffein |
|  |  |  | Eu150 |  | [X]Acute intoxic oth stimulant |
|  |  |  | Eu151 |  | [X]Harmful use other stimulant |
|  |  |  | Eu152 |  | [X]Oth stimulant dependen synd |
|  |  |  | Eu153 |  | [X]Oth stimulant withdr state |
|  |  |  | Eu154 |  | [X]Oth stimulant withdr delir |
|  |  |  | Eu155 |  | [X]Psychotic dis oth stimulant |
|  |  |  | Eu156 |  | [X]Amnesic syndr oth stimulant |
|  |  |  | Eu157 |  | [X]Resid psychot oth stimulant |
|  |  |  | Eu15y |  | [X]Oth ment/beh dis stimulant |
|  |  |  | Eu15z |  | [X]Uns ment/beh dis stimulant |
|  | F16, F160-F169 |  | Eu16. |  | [X]Mental disord hallucinogens |
|  |  |  | Eu160 |  | [X]Acute hallucinogen intoxic |
|  |  |  | Eu161 |  | [X]Harmful use hallucinogens |
|  |  |  | Eu162 |  | [X]Hallucinogen depend synd |
|  |  |  | Eu163 |  | [X]Hallucinogen withdraw state |
|  |  |  | Eu164 |  | [X]Hallucin withdraw delirium |
|  |  |  | Eu165 |  | [X]Psychotic due hallucinogen |
|  |  |  | Eu166 |  | [X]Amnesic synd due hallucinog |
|  |  |  | Eu167 |  | [X]Resid psychot hallucinogen |
|  |  |  | Eu16y |  | [X]Oth ment/beh dis hallucinog |
|  |  |  | Eu16z |  | [X]Uns ment/beh dis hallucinog |
|  | F18, F180-F189 |  | Eu18. |  | [X]Ment dis volatile solvents |
|  |  |  | Eu180 |  | [X]Acute solvent intoxication |
|  |  |  | Eu181 |  | [X]Harmful use of solvents |
|  |  |  | Eu182 |  | [X]Solvent dependence syndrome |
|  |  |  | Eu183 |  | [X]Solvent withdrawal state |
|  |  |  | Eu184 |  | [X]Solvent withdrawal delirium |
|  |  |  | Eu185 |  | [X]Psychotic dis due solvent |
|  |  |  | Eu186 |  | [X]Amnesic syndr due solvent |
|  |  |  | Eu187 |  | [X]Resid psychotic due solvent |
|  |  |  | Eu18y |  | [X]Ot ment/beh dis due solvent |
|  |  |  | Eu18z |  | [X]Uns ment/beh due solvent |
|  | F19, F190-F199 |  | Eu19. |  | [X]Ment disord multi drug use |
|  |  |  | Eu190 |  | [X]Acute intox multi drug use |
|  |  |  | Eu191 |  | [X]Harmful use multiple drugs |
|  |  |  | Eu192 |  | [X]Multiple drug dependence |
|  |  |  | Eu193 |  | [X]Multiple drug withdrawal |
|  |  |  | Eu194 |  | [X]Multi drug withdr delirium |
|  |  |  | Eu195 |  | [X]Psychotic due multi drugs |
|  |  |  | Eu196 |  | [X]Amnesic syn due multi drugs |
|  |  |  | Eu197 |  | [X]Resid psychotic multi drugs |
|  |  |  | Eu19y |  | [X]Ot ment/beh due multi drugs |
|  |  |  | Eu19z |  | [X]Un ment/beh due multi drugs |
|  |  |  | Eu1A. |  | [X]Men behav dis due crack coc |
|  |  |  | Eu1A0 |  | [X]Acute crack cocaine intoxic |
|  |  |  | Eu1A1 |  | [X]Harmful use crack cocaine |
|  |  |  | Eu1A2 |  | [X]Crack cocaine depend synd |
|  |  |  | Eu1A3 |  | [X]Crack cocaine withdraw stat |
|  |  |  | Eu1A4 |  | [X]Crack coc withdraw stat del |
|  |  |  | Eu1A5 |  | [X]Crack cocaine psychotic dis |
|  |  |  | Eu1A6 |  | [X]Crack cocaine amnesic synd |
|  |  |  | Eu1A7 |  | [X]Cra coc res late-on psy dis |
|  |  |  | Eu1Ay |  | [X]Crac coc other ment beh dis |
|  |  |  | Eu1Az |  | [X]Crack coc unsp men beh dis |
|  |  |  | 1365. |  | Heavy drinker - 7-9u/day |
|  |  |  | 1366. |  | Very heavy drinker - >9u/day |
|  |  |  | 1462. |  | H/O: alcoholism |
|  |  |  | 1463. |  | H/O: drug dependency |
|  |  |  | 136c. |  | Higher risk drinking |
|  |  |  | 136P. |  | Heavy drinker |
|  |  |  | 136Q. |  | Very heavy drinker |
|  |  |  | 136R. |  | Binge drinker |
|  |  |  | 136S. |  | Hazardous alcohol use |
|  |  |  | 136T. |  | Harmful alcohol use |
|  |  |  | 136W. |  | Alcohol misuse |
|  |  |  | 136Y. |  | Drink in a.m. to rid hangover |
|  |  |  | 13c.. |  | Drug user |
|  |  |  | 13c0. |  | Injecting drug user |
|  |  |  | 13c1. |  | Intravenous drug user |
|  |  |  | 13c2. |  | Never injecting drug user |
|  |  |  | 13c3. |  | Intramuscular drug user |
|  |  |  | 13c4. |  | Intranasal drug user |
|  |  |  | 13c5. |  | Substance misuse increased |
|  |  |  | 13c6. |  | Substance misuse decreased |
|  |  |  | 13c7. |  | Current drug user |
|  |  |  | 13c8. |  | Reduced drugs misuse |
|  |  |  | 13c9. |  | Subcutaneous drug user |
|  |  |  | 13cA. |  | Smokes drugs |
|  |  |  | 13cB. |  | Misuses drugs orally |
|  |  |  | 13cC. |  | Continuous use of drugs |
|  |  |  | 13cD. |  | Episodic use of drugs |
|  |  |  | 13cE. |  | Prolong high dose use cannabis |
|  |  |  | 13cF. |  | Preoccup with substance misuse |
|  |  |  | 13cG. |  | Drug tolerance |
|  |  |  | 13cG0 |  | Opioid tolerant |
|  |  |  | 13cG1 |  | Opioid naive |
|  |  |  | 13cH. |  | Persistent substance misuse |
|  |  |  | 13cJ. |  | Previously injecting drug user |
|  |  |  | 13cK. |  | Current non recreat drug user |
|  |  |  | 13cL. |  | Has never injected drugs |
|  |  |  | 13cM. |  | Substance misuse |
|  |  |  | 13cM0 |  | Novl psychactve sbstnce misuse |
|  |  |  | 13cM1 |  | Opioid analgesic dependence |
|  |  |  | 13cN. |  | Has nvr shrd drg injctn equipt |
|  |  |  | 13cQ. |  | Behavioural tolerance to drug |
|  |  |  | 13cR. |  | Physical tolerance to drug |
|  |  |  | 13cS. |  | Psychological drug tolerance |
|  |  |  | 13cT. |  | Reverse tolerance to drug |
|  |  |  | 13Y8. |  | Alcoholics anonymous |
|  |  |  | 146C. |  | Failed heroin detoxification |
|  |  |  | 146E. |  | H/O: recreational drug use |
|  |  |  | 146F. |  | H/O: drug abuse |
|  |  |  | 1B1c. |  | Alcohol induced hallucinations |
|  |  |  | 1P30. |  | Compul uncontrollable drug tak |
|  |  |  | 1P31. |  | Compulsive drug taking |
|  |  |  | 1T... |  | History of substance misuse |
|  |  |  | 1T0.. |  | H/O heroin misuse |
|  |  |  | 1T00. |  | H/O daily heroin misuse |
|  |  |  | 1T01. |  | H/O weekly heroin misuse |
|  |  |  | 1T02. |  | Prev history of heroin misuse |
|  |  |  | 1T03. |  | H/O infrequent heroin misuse |
|  |  |  | 1T1.. |  | H/O methadone misuse |
|  |  |  | 1T10. |  | H/O daily methadone misuse |
|  |  |  | 1T11. |  | H/O weekly methadone misuse |
|  |  |  | 1T12. |  | H/O infrequent methadone misus |
|  |  |  | 1T13. |  | Prev history methadone misuse |
|  |  |  | 1T2.. |  | H/O ecstasy misuse |
|  |  |  | 1T20. |  | H/O daily ecstasy misuse |
|  |  |  | 1T21. |  | H/O weekly ecstasy misuse |
|  |  |  | 1T22. |  | H/O infrequent ecstasy misuse |
|  |  |  | 1T23. |  | Prev history of ecstasy misuse |
|  |  |  | 1T3.. |  | H/O benzodiazepine misuse |
|  |  |  | 1T30. |  | H/O daily benzodiazepin misuse |
|  |  |  | 1T31. |  | H/O weekly benzodiazep misuse |
|  |  |  | 1T32. |  | H/O infreq benzodiazep misuse |
|  |  |  | 1T33. |  | Prev H/O benzodiazepine misuse |
|  |  |  | 1T4.. |  | H/O amphetamine misuse |
|  |  |  | 1T40. |  | H/O daily amphetamine misuse |
|  |  |  | 1T41. |  | H/O weekly amphetamine misuse |
|  |  |  | 1T42. |  | H/O infrequent amphetam misuse |
|  |  |  | 1T43. |  | Prev H/O amphetamine misuse |
|  |  |  | 1T5.. |  | H/O cocaine misuse |
|  |  |  | 1T50. |  | H/O daily cocaine misuse |
|  |  |  | 1T51. |  | H/O weekly cocaine misuse |
|  |  |  | 1T52. |  | H/O infrequent cocaine misuse |
|  |  |  | 1T53. |  | Prev H/O cocaine misuse |
|  |  |  | 1T6.. |  | H/O crack cocaine misuse |
|  |  |  | 1T60. |  | H/O daily crack cocaine misuse |
|  |  |  | 1T61. |  | H/O weekly crack cocain misuse |
|  |  |  | 1T62. |  | H/O infrequ crack cocain misus |
|  |  |  | 1T63. |  | Prev H/O crack cocaine misuse |
|  |  |  | 1T7.. |  | H/O hallucinogen misuse |
|  |  |  | 1T70. |  | H/O daily hallucinogen misuse |
|  |  |  | 1T71. |  | H/O weekly hallucinogen misuse |
|  |  |  | 1T72. |  | H/O infrequ hallucinog misuse |
|  |  |  | 1T73. |  | Prev H/O hallucinogen misuse |
|  |  |  | 1T8.. |  | H/O cannabis misuse |
|  |  |  | 1T80. |  | H/O daily cannabis misuse |
|  |  |  | 1T81. |  | H/O weekly cannabis misuse |
|  |  |  | 1T82. |  | H/O infrequent cannabis misuse |
|  |  |  | 1T83. |  | Prev H/O cannabis misuse |
|  |  |  | 1T9.. |  | H/O solvent misuse |
|  |  |  | 1T90. |  | H/O daily solvent misuse |
|  |  |  | 1T91. |  | H/O weekly solvent misuse |
|  |  |  | 1T92. |  | H/O infrequent solvent misuse |
|  |  |  | 1T93. |  | Prev history of solvent misuse |
|  |  |  | 1TA.. |  | H/O barbiturate misuse |
|  |  |  | 1TA0. |  | H/O daily barbiturate misuse |
|  |  |  | 1TA1. |  | H/O weekly barbiturate misuse |
|  |  |  | 1TA2. |  | H/O infrequ barbiturate misuse |
|  |  |  | 1TA3. |  | Prev H/O barbiturate misuse |
|  |  |  | 1TB.. |  | H/O major tranquilliser misuse |
|  |  |  | 1TB0. |  | H/O daily maj tranquilli misus |
|  |  |  | 1TB1. |  | H/O weekl maj tranquilli misus |
|  |  |  | 1TB2. |  | H/O infreq maj trnquillis miss |
|  |  |  | 1TB3. |  | Prev H/O major tranq misuse |
|  |  |  | 1TC.. |  | H/O anti-depressant misuse |
|  |  |  | 1TC0. |  | H/O daily anti-depress misuse |
|  |  |  | 1TC1. |  | H/O weekly anti-depress misuse |
|  |  |  | 1TC2. |  | H/O infreq anti-depress misuse |
|  |  |  | 1TC3. |  | Prev H/O anti-depressnt misuse |
|  |  |  | 1TD.. |  | H/O opiate misuse |
|  |  |  | 1TD0. |  | H/O daily opiate misuse |
|  |  |  | 1TD1. |  | H/O weekly opiate misuse |
|  |  |  | 1TD2. |  | H/O infrequent opiate misuse |
|  |  |  | 1TD3. |  | Prev history of opiate misuse |
|  |  |  | 1TE.. |  | Uses heroin on top subst ther |
|  |  |  | 1TF.. |  | Dsnt use heroin top subst ther |
|  |  |  | 1TG.. |  | H/O nov psychoact subst misuse |
|  |  |  | 1V... |  | Drug misuse behaviour |
|  |  |  | 1V0.. |  | Misuses drugs |
|  |  |  | 1V00. |  | Occasional drug user |
|  |  |  | 1V01. |  | Long-term drug misuser |
|  |  |  | 1V02. |  | Poly-drug misuser |
|  |  |  | 1V03. |  | Misuses drugs sublingually |
|  |  |  | 1V04. |  | Misuses drugs rectally |
|  |  |  | 1V05. |  | Misuses drugs vaginally |
|  |  |  | 1V06. |  | Uses drug paraphernalia |
|  |  |  | 1V07. |  | Notified addict |
|  |  |  | 1V08. |  | Smokes drugs in cigarette form |
|  |  |  | 1V09. |  | Smokes drugs through a pipe |
|  |  |  | 1V0A. |  | Chases the dragon |
|  |  |  | 1V0B. |  | Sniffs drugs |
|  |  |  | 1V0C. |  | Drug addict |
|  |  |  | 1V0D. |  | Am spent per day on drug habit |
|  |  |  | 1V0E. |  | Health prob sec to drug misuse |
|  |  |  | 1V1.. |  | Time devotd drug rel activties |
|  |  |  | 1V10. |  | Time spent obtaining drugs |
|  |  |  | 1V11. |  | Time spent taking drugs |
|  |  |  | 1V12. |  | Time spent recover from drugs |
|  |  |  | 1V2.. |  | Frequency of drug misuse |
|  |  |  | 1V22. |  | Age at starting drug misuse |
|  |  |  | 1V23. |  | Time since stopped drug misuse |
|  |  |  | 1V24. |  | Total time drugs misused |
|  |  |  | 1V26. |  | Misused drugs in past |
|  |  |  | 1V3.. |  | Drug injection behaviour |
|  |  |  | 1V30. |  | Injects drugs subcutaneously |
|  |  |  | 1V31. |  | Injects drugs intramuscularly |
|  |  |  | 1V32. |  | Neck injector |
|  |  |  | 1V33. |  | Groin injector |
|  |  |  | 1V34. |  | Does not inject drugs |
|  |  |  | 1V35. |  | Shares drug equipment |
|  |  |  | 1V36. |  | Frontloading |
|  |  |  | 1V37. |  | Drug inject equipment hygiene |
|  |  |  | 1V38. |  | Sharing drug inject equipment |
|  |  |  | 1V3A. |  | Not share drug inject equipmen |
|  |  |  | 1V3B. |  | Shares syringes |
|  |  |  | 1V3C. |  | Shares needles |
|  |  |  | 1V3D. |  | Cleaning of needles |
|  |  |  | 1V3E. |  | Cleans own needles |
|  |  |  | 1V3F. |  | Cleans needles with bleach |
|  |  |  | 1V3G. |  | Does not clean needles |
|  |  |  | 1V3H. |  | Obtains clean needles |
|  |  |  | 1V3J. |  | Uses needle exchange scheme |
|  |  |  | 1V3K. |  | Obtains clean syringes |
|  |  |  | 1V3L. |  | Needle syringe exch scheme use |
|  |  |  | 1V3M. |  | Needle + syringe exch not used |
|  |  |  | 1V3N. |  | Needle and syringe exch used |
|  |  |  | 1V4.. |  | Priority of drug activity |
|  |  |  | 1V40. |  | No priority to drug activities |
|  |  |  | 1V41. |  | Priority to drug activities |
|  |  |  | 1V42. |  | Drug priority ov social obligs |
|  |  |  | 1V43. |  | Drug priority over family |
|  |  |  | 1V44. |  | Drug priority ov finance oblig |
|  |  |  | 1V5.. |  | Routine drug-related activity |
|  |  |  | 1V50. |  | No routine of drug activities |
|  |  |  | 1V51. |  | Has routine of drug activities |
|  |  |  | 1V52. |  | Same drug routine every day |
|  |  |  | 1V53. |  | Drug-related rituals |
|  |  |  | 1V54. |  | Follows drug-related rituals |
|  |  |  | 1V55. |  | Not follow drug-relate rituals |
|  |  |  | 1V6.. |  | Drug-relat offending behaviour |
|  |  |  | 1V64. |  | Illicit drug use |
|  |  |  | 1V65. |  | Heroin misuse |
|  |  |  | 1V66. |  | Ecstasy misuse |
|  |  |  | 38Dz. |  | Sever alcoh depend questionn |
|  |  |  | 66e.. |  | Alcohol disorder monitoring |
|  |  |  | 66e0. |  | Alcohol abuse monitoring |
|  |  |  | 677T. |  | Subst misuse structurd counsel |
|  |  |  | 7P220 |  | Delivery rehab drug addiction |
|  |  |  | 7P221 |  | Del rehab alcohol addiction |
|  |  |  | 8AA.. |  | Drug abuse monitoring |
|  |  |  | 8B23. |  | Drug addiction therapy |
|  |  |  | 8B230 |  | Drug add maint ther naltrexone |
|  |  |  | 8B231 |  | Drug add maint ther lofexidine |
|  |  |  | 8B2M. |  | Buprenorphine maintenance ther |
|  |  |  | 8B2N. |  | Drug add detox ther methadone |
|  |  |  | 8B2P. |  | Drug add maint ther methadone |
|  |  |  | 8B2Q. |  | Drug add maint ther buprenorph |
|  |  |  | 8B2R. |  | Drug add detox ther buprenorph |
|  |  |  | 8B2S. |  | Opioid agonist substitut thera |
|  |  |  | 8B2T. |  | Opioid antagonist therapy |
|  |  |  | 8BA8. |  | Alcohol detoxification |
|  |  |  | 8BA9. |  | Detoxification dependence drug |
|  |  |  | 8BAc. |  | Subs mis mgt stop - self withd |
|  |  |  | 8BAd. |  | Opiate dependence detoxificatn |
|  |  |  | 8BAs. |  | Alcohol relapse prevention |
|  |  |  | 8BAt. |  | Drug relapse prevention |
|  |  |  | 8BAu. |  | Alcohol harm reduction prog |
|  |  |  | 8BAv. |  | Drug harm reduction programme |
|  |  |  | 8BAw. |  | Alcohol twelve step programme |
|  |  |  | 8BAW. |  | Drug depen self detoxification |
|  |  |  | 8BAX. |  | Drug depen home detoxification |
|  |  |  | 8BAx. |  | Drug twelve step programme |
|  |  |  | 8BE.. |  | Maintenance therapy |
|  |  |  | 8BE0. |  | Reinduct methadone maint thera |
|  |  |  | 8BE1. |  | Reinduct buprenorph maint ther |
|  |  |  | 8CAv. |  | Adv cont prim care alcoh workr |
|  |  |  | 8CR9. |  | Benzodiazepi clinical mgt plan |
|  | Z503 |  | 8FB.. |  | Drug rehabilitation |
|  |  |  | 8FB0. |  | Drug detox programme completed |
|  |  |  | 8G32. |  | Aversion therapy - alcoholism |
|  |  |  | 8H35. |  | Admit to alcohol detox centre |
|  |  |  | 8H7p. |  | Refer community alcohol team |
|  |  |  | 8H7x. |  | Refer to drug abuse counsellor |
|  |  |  | 8Hh1. |  | Self refer substanc misus serv |
|  |  |  | 8HHd. |  | Referral to drug treatment cen |
|  |  |  | 8HHe. |  | Referral to com drug alco team |
|  |  |  | 8HHL. |  | Ref to comm drug dependen team |
|  |  |  | 8HkF. |  | Refer substance misuse service |
|  |  |  | 8HkG. |  | Ref to special alco treat serv |
|  |  |  | 8HkJ. |  | Ref alchl brief intervntin ser |
|  |  |  | 8Hl5. |  | Referral to drugs therapist |
|  |  |  | 8Hl6. |  | Referral to drugs worker |
|  |  |  | 8Hq.. |  | Admsn substnc misuse detox cnt |
|  |  |  | 8I2N. |  | Drug depend home detox contra |
|  |  |  | 8IAF. |  | Brief intervent ex alc declind |
|  |  |  | 8IAJ. |  | Decld ref spec alco treat serv |
|  |  |  | 8IAt. |  | Ext inter exc alco consump dec |
|  |  |  | 8IE7. |  | Substance misuse assess declin |
|  |  |  | 8IEA. |  | Ref comm alcohol team declined |
|  |  |  | 9G2.. |  | Drug addiction notification |
|  |  |  | 9G21. |  | Drug addict notific to CMO |
|  |  |  | 9G22. |  | Drug addict re-notific due |
|  |  |  | 9G23. |  | Drug addict re-notif to CMO |
|  |  |  | 9G2Z. |  | Drug addiction notif NOS |
|  |  |  | 9HC.. |  | Substance misuse monitoring |
|  |  |  | 9HC0. |  | Initial substance misuse asses |
|  |  |  | 9HC1. |  | Follow up substa misuse assess |
|  |  |  | 9HC2. |  | Subst mis clin man plan agreed |
|  |  |  | 9HC3. |  | Subst mis clin man plan review |
|  |  |  | 9HC4. |  | Sub misuse treatment withdrawn |
|  |  |  | 9HC5. |  | Sub misus treat prog completed |
|  |  |  | 9HC6. |  | Substance misuse treatm declin |
|  |  |  | 9HC7. |  | Subst misuse treat not availbl |
|  |  |  | 9HC8. |  | Decl to give subst misuse hist |
|  |  |  | 9HC9. |  | Snc mse tmnt gvn othr hcr prdr |
|  |  |  | 9HCA. |  | Sbstnce misuse mntr 6 mnth rvw |
|  |  |  | 9HCB. |  | Substance misuse mntr annl rvw |
|  |  |  | 9HCC. |  | On substance misuse programme |
|  |  |  | 9k1.. |  | Alcohol misuse - enh ser admin |
|  |  |  | 9k10. |  | Comm detoxification registered |
|  |  |  | 9k11. |  | Alcohol consumption counsellin |
|  |  |  | 9k12. |  | Alco misuse - enh serv complet |
|  |  |  | 9k14. |  | Alco counsel by other agencies |
|  |  |  | 9k19. |  | Alco asses dclnd - enh ser adm |
|  |  |  | 9k1A. |  | Brief intervent ex alc complet |
|  |  |  | 9k1B. |  | Extend intervent ex alc complt |
|  |  |  | 9k5.. |  | Drug misuse - enhan serv admin |
|  |  |  | 9k50. |  | Drug misuse - enh serv complet |
|  |  |  | 9k51. |  | Share care drug misu trt - ESA |
|  |  |  | 9k52. |  | Drug misus trt prim care - ESA |
|  |  |  | 9k53. |  | Phrmcy attend drug misus - ESA |
|  |  |  | 9kS.. |  | Drug mis asse decl - enha serv |
|  |  |  | 9N0Z. |  | Seen in drug rehab centre |
|  |  |  | 9N1yJ |  | Seen in drug misuse clinic |
|  |  |  | 9N6a. |  | Refer by drug statutor service |
|  |  |  | 9N6b. |  | Ref by drug non-statutory serv |
|  |  |  | 9N6g. |  | Refer by syringe excha service |
|  |  |  | 9NdN. |  | Declnd consnt notif drug misus |
|  |  |  | 9NgzH |  | Withdrawn alcohol detox progra |
|  |  |  | 9NJz. |  | In-house alcohol detoxificatin |
|  |  |  | 9NN1. |  | Under care community drug team |
|  |  |  | 9NN2. |  | Under care comm alcohol team |
|  |  |  | 9No5. |  | Seen in substance misuse clinc |
|  |  |  | 9NX2. |  | In-house subs misuse treatment |
|  |  |  | 9Nz9. |  | Emrgcy dept attn alcohl consum |
|  |  |  | 9NzA. |  | Hospital attend alcohl consump |
|  | E244 |  | C1505 |  | Alchl-indc pseud-Cushings syn |
|  | E512 |  | C253. |  | Wernickes encephalopathy |
|  |  |  | dj36. |  | SUBUTEX 400micrograms s/l tabs |
|  |  |  | dj37. |  | SUBUTEX 2mg sublingual tablets |
|  |  |  | dj38. |  | SUBUTEX 8mg sublingual tablets |
|  |  |  | dj3c. |  | PREFIBIN 400mcg sublingual tab |
|  |  |  | dj3d. |  | PREFIBIN 2mg sublingual tabs |
|  |  |  | dj3D. |  | BUPRNRPHNE+NALOXN 2/0.5mg tabs |
|  |  |  | dj3E. |  | SUBOXONE 2mg/0.5mg s/l tabs |
|  |  |  | dj3e. |  | PREFIBIN 8mg sublingual tabs |
|  |  |  | dj3F. |  | BUPRNRPHNE+NALOXN 8mg/2mg tabs |
|  |  |  | dj3G. |  | SUBOXONE 8mg/2mg s/l tabs |
|  |  |  | dj3K. |  | NATZON 400micrograms s/l tabs |
|  |  |  | dj3L. |  | NATZON 2mg sublingual tablets |
|  |  |  | dj3M. |  | NATZON 8mg sublingual tablets |
|  |  |  | dj3N. |  | GABUP 400micrograms s/l tabs |
|  |  |  | dj3O. |  | GABUP 1mg sublingual tablets |
|  |  |  | dj3P. |  | GABUP 2mg sublingual tablets |
|  |  |  | dj3Q. |  | GABUP 4mg sublingual tablets |
|  |  |  | dj3R. |  | GABUP 6mg sublingual tablets |
|  |  |  | dj3S. |  | GABUP 8mg sublingual tablets |
|  |  |  | dj3T. |  | BUPRENORPHINE 1mg s/l tabs |
|  |  |  | dj3U. |  | BUPRENORPHINE 4mg s/l tabs |
|  |  |  | dj3u. |  | BUPRENORPHINE 2mg s/l tabs |
|  |  |  | dj3V. |  | BUPRENORPHINE 6mg s/l tabs |
|  |  |  | dj3v. |  | BUPRENORPHINE 8mg s/l tabs |
|  |  |  | djc.. |  | METHADONE HCL [ANALGESIC] |
|  |  |  | djc1. |  | PHYSEPTONE 5mg tablets |
|  |  |  | djc2. |  | PHYSEPTONE 10mg/1mL injection |
|  |  |  | djc3. |  | METHADONE 1mg/1mL mixture |
|  |  |  | djc4. |  | METHADONE HCL 50mg/5mL s/f liq |
|  |  |  | djc5. |  | MARTINDALE METHADONE DTF mixt |
|  |  |  | djc6. |  | METHODEX 1mg/1mL mixture |
|  |  |  | djc7. |  | METHADOSE 10mg/mL s/f liq |
|  |  |  | djc8. |  | METHADONE HCL 20mg/mL s/f liq |
|  |  |  | djc9. |  | METHADOSE 20mg/mL s/f liq |
|  |  |  | djcA. |  | METHADONE DILUENT liquid |
|  |  |  | djcB. |  | METHADOSE DILUENT liquid |
|  |  |  | djcC. |  | METHADONE 1mg/1mL s/f mixt |
|  |  |  | djcD. |  | METHAROSE 1mg/1mL s/f soln |
|  |  |  | djcE. |  | *PINADONE 1mg/1mL mixture |
|  |  |  | djcF. |  | *PINADONE 1mg/1mL s/f mixt |
|  |  |  | djcG. |  | PHYSEPTONE 20mg/2mL injection |
|  |  |  | djcH. |  | PHYSEPTONE 35mg/3.5mL inj |
|  |  |  | djcJ. |  | PHYSEPTONE 50mg/5mL injection |
|  |  |  | djcK. |  | PHYSEPTONE 1mg/1mL s/f mixture |
|  |  |  | djcL. |  | PHYSEPTONE 1mg/1mL mixture |
|  |  |  | djcM. |  | SYNASTONE 10mg/1mL injection |
|  |  |  | djcN. |  | SYNASTONE 20mg/2mL injection |
|  |  |  | djco. |  | METHADONE 20mg/20mL oral soln |
|  |  |  | djcO. |  | SYNASTONE 35mg/3.5mL injection |
|  |  |  | djcP. |  | SYNASTONE 50mg/5mL injection |
|  |  |  | djcp. |  | METHADONE 40mg/40mL oral soln |
|  |  |  | djcQ. |  | SYNASTONE 50mg/2mL injection |
|  |  |  | djcq. |  | METHADONE 60mg/60mL oral soln |
|  |  |  | djcR. |  | SYNASTONE 50mg/1mL injection |
|  |  |  | djcr. |  | METHADONE 100mg/20mL oral soln |
|  |  |  | djcS. |  | PHYSEPTONE 50mg/2mL injection |
|  |  |  | djcs. |  | METHADONE 5mg/mL oral solution |
|  |  |  | djct. |  | METHADONE HCL 50mg/2mL inj |
|  |  |  | djcT. |  | PHYSEPTONE 50mg/1mL injection |
|  |  |  | djcu. |  | METHADONE HCL 50mg/1mL inj |
|  |  |  | djcU. |  | EPTADONE 1mg/mL oral solution |
|  |  |  | djcV. |  | EPTADONE 5mg/mL oral solution |
|  |  |  | djcv. |  | METHADONE HCL 20mg/2mL inj |
|  |  |  | djcW. |  | EPTADONE 20mg/20mL oral soln |
|  |  |  | djcw. |  | METHADONE HCL 35mg/3.5mL inj |
|  |  |  | djcx. |  | METHADONE HCL 50mg/5mL inj |
|  |  |  | djcX. |  | EPTADONE 40mg/40mL oral soln |
|  |  |  | djcY. |  | EPTADONE 60mg/60mL oral soln |
|  |  |  | djcy. |  | METHADONE HCL 5mg tablets |
|  |  |  | djcz. |  | METHADONE HCL 10mg/1mL inj |
|  |  |  | djcZ. |  | EPTADONE 100mg/20mL oral soln |
|  |  |  | du1.. |  | DISULFIRAM |
|  |  |  | du11. |  | DISULFIRAM 200mg tablets |
|  |  |  | du12. |  | ANTABUSE 200mg tablets |
|  |  |  | du2.. |  | NALTREXONE HYDROCHLORIDE |
|  |  |  | du21. |  | NALTREXONE HCL 50mg tablets |
|  |  |  | du22. |  | NALOREX 50mg tablets |
|  |  |  | du23. |  | OPIZONE 50mg tablets |
|  |  |  | du24. |  | ADEPEND 50mg tablets |
|  |  |  | du4.. |  | LOFEXIDINE HYDROCHLORIDE |
|  |  |  | du41. |  | BRITLOFEX 200mcg tablets |
|  |  |  | du42. |  | LOFEXIDINE HCL 200mcg tablets |
|  |  |  | du5.. |  | ACAMPROSATE CALCIUM |
|  |  |  | du51. |  | ACAMPROSATE CAL 333mg e/c tabs |
|  |  |  | du52. |  | CAMPRAL EC 333mg e/c tablets |
|  |  |  | E23.. |  | Alcohol dependence syndrome |
|  |  |  | E230. |  | Acute alcoholic intoxication |
|  |  |  | E2300 |  | Acute alcoholic intoxic. unsp. |
|  |  |  | E2301 |  | Acute alcoh.intox.-continuous |
|  |  |  | E2302 |  | Acute alcoh.intox.-episodic |
|  |  |  | E2303 |  | Acute alcoh.intox.in remission |
|  |  |  | E230z |  | Acute alcoholic intoxic. NOS |
|  |  |  | E231. |  | Chronic alcoholism |
|  |  |  | E2310 |  | Chronic alcoholism unspecified |
|  |  |  | E2311 |  | Chronic alcoholism-continuous |
|  |  |  | E2312 |  | Chronic alcoholism-episodic |
|  |  |  | E2313 |  | Chronic alcohol.- in remission |
|  |  |  | E231z |  | Chronic alcoholism NOS |
|  |  |  | E23z. |  | Alcohol dependence syndr. NOS |
|  |  |  | E24.. |  | Drug dependence |
|  |  |  | E240. |  | Opioid type drug dependence |
|  |  |  | E2400 |  | Opioid dependence-unspecified |
|  |  |  | E2401 |  | Opioid dependence-continuous |
|  |  |  | E2402 |  | Opioid dependence - episodic |
|  |  |  | E2403 |  | Opioid dependence-in remission |
|  |  |  | E240z |  | Opioid drug dependence NOS |
|  |  |  | E241. |  | Hypnotic/anxiolytic dependence |
|  |  |  | E2410 |  | Hypnotic/anxiol.depend.-unspec |
|  |  |  | E2411 |  | Hypnot/anxiol.dep.-continuous |
|  |  |  | E2412 |  | Hypnot/anxiol.dep.-episodic |
|  |  |  | E2413 |  | Hypnot/anxiol.dep-in remission |
|  |  |  | E241z |  | Hypnotic/anxiolytic depend.NOS |
|  |  |  | E242. |  | Cocaine type drug dependence |
|  |  |  | E2420 |  | Cocaine dependence-unspecified |
|  |  |  | E2421 |  | Cocaine dependence-continuous |
|  |  |  | E2422 |  | Cocaine dependence-episodic |
|  |  |  | E2423 |  | Cocaine depend. - in remission |
|  |  |  | E242z |  | Cocaine drug dependence NOS |
|  |  |  | E243. |  | Cannabis type drug dependence |
|  |  |  | E2430 |  | Cannabis dependence-unspecif. |
|  |  |  | E2431 |  | Cannabis dependence-continuous |
|  |  |  | E2432 |  | Cannabis dependence-episodic |
|  |  |  | E2433 |  | Cannabis depend.- in remission |
|  |  |  | E243z |  | Cannabis drug dependence NOS |
|  |  |  | E244. |  | Amphetamine/psychostim.depend. |
|  |  |  | E2440 |  | Amphetamine depend.-unspecif. |
|  |  |  | E2441 |  | Amphetamine depend.-continuous |
|  |  |  | E2442 |  | Amphetamine depend.-episodic |
|  |  |  | E2443 |  | Amphetamine dep.-in remission |
|  |  |  | E244z |  | Amphetamine dependence NOS |
|  |  |  | E245. |  | Hallucinogen dependence |
|  |  |  | E2450 |  | Hallucinogen depend.-unspecif. |
|  |  |  | E2451 |  | Hallucinogen depend-continuous |
|  |  |  | E2452 |  | Hallucinogen depend.-episodic |
|  |  |  | E2453 |  | Hallucinogen dep.-in remission |
|  |  |  | E245z |  | Hallucinogen dependence NOS |
|  |  |  | E246. |  | Glue sniffing dependence |
|  |  |  | E2460 |  | Glue sniffing - unspecified |
|  |  |  | E2461 |  | Glue sniffing - continuous |
|  |  |  | E2462 |  | Glue sniffing - episodic |
|  |  |  | E2463 |  | Glue sniffing - in remission |
|  |  |  | E246z |  | Glue sniffing dependence NOS |
|  |  |  | E247. |  | Other specified drug dependen. |
|  |  |  | E2470 |  | Other drug dependence unspecif |
|  |  |  | E2471 |  | Other drug depend.-continuous |
|  |  |  | E2472 |  | Other drug depend.-episodic |
|  |  |  | E2473 |  | Other drug dep.-in remission |
|  |  |  | E247z |  | Other drug dependence NOS |
|  |  |  | E248. |  | Combined opioid+other drug dep |
|  |  |  | E2480 |  | Opioid+other drug dep. unspec. |
|  |  |  | E2481 |  | Continuous opioid+other depen. |
|  |  |  | E2482 |  | Episodic opioid+other depend. |
|  |  |  | E2483 |  | In remission-opioid+other dep. |
|  |  |  | E248z |  | Opioid+other drug depend. NOS |
|  |  |  | E249. |  | Combined drug dep. excl.opioid |
|  |  |  | E2490 |  | Comb.drug dep ex opioid-unspec |
|  |  |  | E2491 |  | Comb.drug dep ex opioid-contin |
|  |  |  | E2492 |  | Comb.drug dep ex opioid-episod |
|  |  |  | E2493 |  | Comb.drug dep ex opioid-in rem |
|  |  |  | E249z |  | Comb.drug dep ex opioid NOS |
|  |  |  | E24A. |  | Ecstasy type drug dependence |
|  |  |  | E24z. |  | Drug dependence NOS |
|  |  |  | E25.. |  | Nondependent abuse of drugs |
|  |  |  | E250. |  | Alcohol abuse - nondependent |
|  |  |  | E2500 |  | Alcohol abuse - unspecified |
|  |  |  | E2501 |  | Alcohol abuse - continuous |
|  |  |  | E2502 |  | Alcohol abuse - episodic |
|  |  |  | E2503 |  | Alcohol abuse - in remission |
|  |  |  | E250z |  | Nondependent alcohol abuse NOS |
|  |  |  | E252. |  | Nondependent cannabis abuse |
|  |  |  | E2520 |  | Nondep cannabis abuse - unspec |
|  |  |  | E2521 |  | Nondep cannabis abuse - contin |
|  |  |  | E2522 |  | Nondep cannabis abuse - episod |
|  |  |  | E2523 |  | Nondep cannabis abuse in remis |
|  |  |  | E252z |  | Nondep cannabis abuse NOS |
|  |  |  | E253. |  | Nondependen hallucinogen abuse |
|  |  |  | E2530 |  | Nondep hallucinogen abuse-unsp |
|  |  |  | E2531 |  | Nondep hallucinogen abuse-cont |
|  |  |  | E2532 |  | Nondep hallucinogen abuse-epis |
|  |  |  | E2533 |  | Nondep hallucin abuse-in remis |
|  |  |  | E253z |  | Nondep hallucinogen abuse NOS |
|  |  |  | E254. |  | Nondep hypnot/anxiolytic abuse |
|  |  |  | E2540 |  | Nondep hypnot/anxio.abuse-unsp |
|  |  |  | E2541 |  | Nondep hypnot/anxio.abuse-cont |
|  |  |  | E2542 |  | Nondep hypnot/anxio.abuse-epis |
|  |  |  | E2543 |  | Nondep hypn/anxio.abuse-in rem |
|  |  |  | E254z |  | Nondep hypnot/anxiol abuse NOS |
|  |  |  | E255. |  | Nondependent opioid abuse |
|  |  |  | E2550 |  | Nondep opioid abuse - unspecif |
|  |  |  | E2551 |  | Nondep opioid abuse - continuo |
|  |  |  | E2552 |  | Nondep opioid abuse - episodic |
|  |  |  | E2553 |  | Nondep opioid abuse - in remis |
|  |  |  | E255z |  | Nondependent opioid abuse NOS |
|  |  |  | E256. |  | Nondependent cocaine abuse |
|  |  |  | E2560 |  | Nondep cocaine abuse - unspec. |
|  |  |  | E2561 |  | Nondep cocaine abuse - contin. |
|  |  |  | E2562 |  | Nondep cocaine abuse - episod. |
|  |  |  | E2563 |  | Nondep cocaine abuse -in remis |
|  |  |  | E256z |  | Nondependent cocaine abuse NOS |
|  |  |  | E257. |  | Nondep amphetamine type abuse |
|  |  |  | E2570 |  | Nondep amphet type abuse -unsp |
|  |  |  | E2571 |  | Nondep amphet type abuse -cont |
|  |  |  | E2572 |  | Nondep amphet type abuse -epis |
|  |  |  | E2573 |  | Nondep amph. type abuse-in rem |
|  |  |  | E257z |  | Nondep amphet. type abuse NOS |
|  |  |  | E258. |  | Nondep antidepress type abuse |
|  |  |  | E2580 |  | Nondep antidep type abuse-unsp |
|  |  |  | E2581 |  | Nondep antidep type abuse-cont |
|  |  |  | E2582 |  | Nondep antidep type abuse-epis |
|  |  |  | E2583 |  | Nondep antidep tp abuse-in rem |
|  |  |  | E258z |  | Nondep antidep type abuse NOS |
|  |  |  | E259. |  | Nondependent mixed drug abuse |
|  |  |  | E2590 |  | Nondep mixed drug abuse-unspec |
|  |  |  | E2591 |  | Nondep mixed drug abuse-contin |
|  |  |  | E2592 |  | Nondep mixed drug abuse-episod |
|  |  |  | E2593 |  | Nondep mixed drug abuse-in rem |
|  |  |  | E2594 |  | Misuse of prescription drugs |
|  |  |  | E259z |  | Nondep mixed drug abuse NOS |
|  |  |  | E25y. |  | Nondependent other drug abuse |
|  |  |  | E25y0 |  | Nondep other drug abuse-unspec |
|  |  |  | E25y1 |  | Nondep other drug abuse-contin |
|  |  |  | E25y2 |  | Nondep other drug abuse-episod |
|  |  |  | E25y3 |  | Nondep other drug abuse-in rem |
|  |  |  | E25yz |  | Nondep other drug abuse NOS |
|  |  |  | E25z. |  | Misuse of drugs NOS |
|  | G312 |  |  |  | Degeneration of nervous system due to alcohol |
|  |  |  | F11x0 |  | Cerebral degenerat.-alcoholism |
|  |  |  | F1440 |  | Cerebellar ataxia-alcoholism |
|  |  |  | F25B. |  | Alcohol-induced epilepsy |
|  | G621 |  | F375. |  | Alcoholic polyneuropathy |
|  | G721 |  | F3941 |  | Alcoholic myopathy |
|  | I426 |  | G555. |  | Alcoholic cardiomyopathy |
|  |  |  | G8523 |  | Oes varic alcohol cirr liver |
|  | K292 |  | J153. |  | Alcoholic gastritis |
|  | K70 |  |  |  | Alcoholic liver disease |
|  | K700 |  | J610. |  | Alcoholic fatty liver |
|  |  |  | J611. |  | Acute alcoholic hepatitis |
|  | K703 |  | J612. |  | Alcoholic cirrhosis of liver |
|  | K702 |  | J6120 |  | Alcoholic fibrosis and scleros |
|  |  |  | J613. |  | Alcoholic liver damage unspec. |
|  | K704 |  | J6130 |  | Alcoholic hepatic failure |
|  | K701 |  | J617. |  | Alcoholic hepatitis |
|  |  |  | J6170 |  | Chronic alcoholic hepatitis |
|  | K709 |  |  |  | Alcoholic liver disease, unspecified |
|  | K852 |  | J6708 |  | Alcohol-induced acute panc |
|  | K860 |  | J6710 |  | Alcohol-ind chron pancreatitis |
|  |  |  | L183. |  | Pregnancy+drug dependence |
|  |  |  | L1830 |  | Preg.+drug dependence unspecif |
|  |  |  | L1831 |  | Preg.+drug dependence-deliver. |
|  |  |  | L1832 |  | Preg.+drug depend-del+p/n comp |
|  |  |  | L1833 |  | Preg.+drug depend-not deliver. |
|  |  |  | L1834 |  | Preg.+drug depend.+p/n complic |
|  |  |  | L183z |  | Preg.+drug dependence NOS |
|  |  |  | L255. |  | Fetus+drug damage |
|  |  |  | L2550 |  | Fetus+drug damage unspecified |
|  |  |  | L2551 |  | Fetus+drug damage-delivered |
|  |  |  | L2552 |  | Fetus+drug damage+a/n problem |
|  | O354 |  | L2553 |  | Mat care,susp dam fet from alc |
|  | O355 |  |  |  | Mat care susp dam fet by drugs |
|  |  |  | L255z |  | Fetus+drug damage NOS |
|  |  |  | R103. |  | [D]Alcohol blood level excess. |
|  | R782 |  | R10B0 |  | [D]Finding of cocain in blood |
|  | R783 |  | R10B1 |  | [D]Find hallucinogen in blood |
|  |  |  | R10B2 |  | [D]Find psychotrop drug blood |
|  | R781 |  | R10B4 |  | [D]Finding, opiate drug in bld |
|  | R785 |  | Ryu86 |  | [X]Find ot drg addic poten,bld |
|  |  |  | SL50. |  | Opiate/narcotic poisoning |
|  |  |  | SL500 |  | Unspecified opium poisoning |
|  | T401 |  | SL501 |  | Heroin poisoning |
|  | T403 |  | SL502 |  | Methadone poisoning |
|  |  |  | SL50z |  | Opiate/narcotic poisoning NOS |
|  | T405 |  | SL850 |  | Cocaine poisoning |
|  |  |  | SL96. |  | Hallucinogen poisoning |
|  | T407 |  | SL960 |  | Cannabis poisoning |
|  | T408 |  | SL961 |  | Lysergide (LSD) poisoning |
|  |  |  | SL963 |  | Mescaline poisoning |
|  |  |  | SL964 |  | Psilocybin poisoning |
|  |  |  | SL96z |  | Hallucinogen poisoning NOS |
|  |  |  | SL97. |  | Psychostimulant poisoning |
|  |  |  | SL970 |  | Amfetamine poisoning |
|  |  |  | SL972 |  | Ecstasy poisoning |
|  |  |  | SL97z |  | Psychostimulant poisoning NOS |
|  | T436 |  |  |  | Psychostimulants with abust potential |
|  |  |  | SLH3. |  | Alcohol deterrent poisoning |
|  | T51 |  | SM0.. |  | Alcohol - toxic effect |
|  |  |  | SM00. |  | Ethyl alcohol - toxic effect |
|  | T510 |  | SM000 |  | Ethanol - toxic effect |
|  |  |  | SM001 |  | Denatured alcohol-toxic effect |
|  |  |  | SM002 |  | Grain alcohol - toxic effect |
|  |  |  | SM00z |  | Ethyl alcohol-toxic effect NOS |
|  |  |  | SM01. |  | Methyl alcohol - toxic effect |
|  | T511 |  | SM010 |  | Methanol - toxic effect |
|  |  |  | SM011 |  | Wood alcohol - toxic effect |
|  |  |  | SM01z |  | Methyl alcohol-toxic eff.NOS |
|  | T512 |  | SM02. |  | Isopropyl alcohol-toxic effect |
|  |  |  | SM020 |  | Dimethyl carbinol-toxic effect |
|  |  |  | SM021 |  | Isopropanol - toxic effect |
|  |  |  | SM022 |  | Rubbing alcohol - toxic effect |
|  |  |  | SM02z |  | Isopropyl alcohol-tox.eff.NOS |
|  | T513 |  | SM03. |  | Fusel oil - toxic effect |
|  |  |  | SM030 |  | Amyl alcohol - toxic effect |
|  |  |  | SM031 |  | Butyl alcohol - toxic effect |
|  |  |  | SM032 |  | Propyl alcohol - toxic effect |
|  |  |  | SM03z |  | Fusel oil - toxic effect NOS |
|  | T518 |  | SM0y. |  | Other alcohol - toxic effect |
|  |  |  | SM0z. |  | Alcohol - toxic effect NOS |
|  | T402 |  | SyuFB |  | [X]Poisoning by other opioids |
|  | T404 |  | SyuFC |  | [X]Poisoning by oth synth narc |
|  | T406 |  | SyuFD |  | [X]Poisoning by oth/unsp narc |
|  | T409 |  | SyuFE |  | [X]Pois,oth/un psychodysl/hall |
|  | T519 |  | SyuG0 |  | [X]Toxic eff of oth alcohols |
|  |  |  | T800. |  | Accid.pois.- heroin |
|  |  |  | T801. |  | Accid.pois.- methadone |
|  |  |  | T8023 |  | Accid.pois.- opium |
|  |  |  | T841. |  | Accid.pois.- hallucinogens |
|  |  |  | T8410 |  | Accid.pois.- cannabis derivat. |
|  |  |  | T8413 |  | Accid.pois.- mescaline |
|  |  |  | T8414 |  | Accid.pois.- psilocin |
|  |  |  | T8415 |  | Accid.pois.- psilocybin |
|  |  |  | T842. |  | Accid.pois.- psychostimulants |
|  |  |  | T8420 |  | Accid.pois.- amphetamine |
|  |  |  | T8520 |  | Accid.pois.- cocaine |
|  |  |  | T900. |  | Accid.pois.- alcoholic drinks |
|  |  |  | T9010 |  | Accid.pois.- denatured alcohol |
|  |  |  | T9011 |  | Accid.pois.- methylated spirit |
|  |  |  | T902. |  | Accid.pois.- methyl alcohol |
|  |  |  | T903. |  | Accid.pois.- isopropyl alcohol |
|  |  |  | T9032 |  | Accid.pois.- rubbing alc.subst |
|  |  |  | T904. |  | Accid.pois.- fusel oil |
|  | X42,X420-X429 |  | U1A5. |  | [X]Accident poisoning narcotic |
|  |  |  | U1A50 |  | [X]Acc poison narcotic home |
|  |  |  | U1A51 |  | [X]Ac pois narcotic res ins |
|  |  |  | U1A52 |  | [X]Ac pois narcotic pub ins |
|  |  |  | U1A53 |  | [X]Ac pois narcotic sport ar |
|  |  |  | U1A54 |  | [X]Ac pois narcotic on hway |
|  |  |  | U1A55 |  | [X]Ac pois narcotic trade ar |
|  |  |  | U1A56 |  | [X]Ac pois narcotic indus ar |
|  |  |  | U1A57 |  | [X]Ac pois narcotic on farm |
|  |  |  | U1A5y |  | [X]Ac pois narcotic OS place |
|  |  |  | U1A5z |  | [X]Ac pois narcotic unsp pl |
|  |  |  | U1A6. |  | [X]Acc poisoning hallucinogens |
|  |  |  | U1A60 |  | [X]Acc poison hallucinog home |
|  |  |  | U1A61 |  | [X]Ac pois hallucinog res ins |
|  |  |  | U1A62 |  | [X]Ac pois hallucinog pub ins |
|  |  |  | U1A63 |  | [X]Ac pois hallucinog sport ar |
|  |  |  | U1A64 |  | [X]Ac pois hallucinog on hway |
|  |  |  | U1A65 |  | [X]Ac pois hallucinog trade ar |
|  |  |  | U1A66 |  | [X]Ac pois hallucinog indus ar |
|  |  |  | U1A67 |  | [X]Ac pois hallucinog on farm |
|  |  |  | U1A6y |  | [X]Ac pois hallucinog OS place |
|  |  |  | U1A6z |  | [X]Ac pois hallucinog unsp pl |
|  | X45,X450-X459 |  | U1A9. |  | [X]Accid poisoning by alcohol |
|  |  |  | U1A90 |  | [X]Acc poison alcohol home |
|  |  |  | U1A91 |  | [X]Ac pois alcohol res instit |
|  |  |  | U1A92 |  | [X]Ac pois alcohol pub instit |
|  |  |  | U1A93 |  | [X]Ac pois alcohol sport area |
|  |  |  | U1A94 |  | [X]Ac pois alcohol on highway |
|  |  |  | U1A95 |  | [X]Ac pois alcohol trade area |
|  |  |  | U1A96 |  | [X]Ac pois alcohol indust area |
|  |  |  | U1A97 |  | [X]Ac pois alcohol on farm |
|  |  |  | U1A9y |  | [X]Ac pois alcohol OS place |
|  |  |  | U1A9z |  | [X]Ac pois alcohol unsp place |
|  | X62,X620-X629 |  | U205. |  | [X]Intent self poison narcotic |
|  |  |  | U2050 |  | [X]Self pois narcotic home |
|  |  |  | U2051 |  | [X]S/pois narcotic res ins |
|  |  |  | U2052 |  | [X]S/pois narcotic pub ins |
|  |  |  | U2053 |  | [X]S/pois narcotic sport ar |
|  |  |  | U2054 |  | [X]S/pois narcotic on hway |
|  |  |  | U2055 |  | [X]S/pois narcotic trade ar |
|  |  |  | U2056 |  | [X]S/pois narcotic indus ar |
|  |  |  | U2057 |  | [X]S/pois narcotic on farm |
|  |  |  | U205y |  | [X]S/pois narcotic OS place |
|  |  |  | U205z |  | [X]S/pois narcotic unsp pl |
|  |  |  | U206. |  | [X]Int s/poising hallucinogens |
|  |  |  | U2060 |  | [X]Self pois hallucinog home |
|  |  |  | U2061 |  | [X]S/pois hallucinog res ins |
|  |  |  | U2062 |  | [X]S/pois hallucinog pub ins |
|  |  |  | U2063 |  | [X]S/pois hallucinog sport ar |
|  |  |  | U2064 |  | [X]S/pois hallucinog on hway |
|  |  |  | U2065 |  | [X]S/pois hallucinog trade ar |
|  |  |  | U2066 |  | [X]S/pois hallucinog indus ar |
|  |  |  | U2067 |  | [X]S/pois hallucinog on farm |
|  |  |  | U206y |  | [X]S/pois hallucinog OS place |
|  |  |  | U206z |  | [X]S/pois hallucinog unsp pl |
|  | X65, X650-X659 |  |  |  | Int s/poisoning by alcohol |
|  |  |  | U209. |  | [X]Self poisoning by alcohol |
|  |  |  | U2090 |  | [X]Self pois alcohol home |
|  |  |  | U2091 |  | [X]S/pois alcohol res instit |
|  |  |  | U2092 |  | [X]S/pois alcohol pub instit |
|  |  |  | U2093 |  | [X]S/pois alcohol sport area |
|  |  |  | U2094 |  | [X]S/pois alcohol on highway |
|  |  |  | U2095 |  | [X]S/pois alcohol trade area |
|  |  |  | U2096 |  | [X]S/pois alcohol indust area |
|  |  |  | U2097 |  | [X]S/pois alcohol on farm |
|  |  |  | U209y |  | [X]S/pois alcohol OS place |
|  |  |  | U209z |  | [X]S/pois alcohol unsp place |
|  | Y12, Y120-Y129 |  |  |  | Poisoning by exposure to narcotic and psychodysleptics, NEC, ?intent |
|  |  |  | U405. |  | [X]Poisoning ?intent narcotic |
|  |  |  | U4050 |  | [X]Pois ?intent narcotic home |
|  |  |  | U4051 |  | [X]Pois ?int narcotic resid |
|  |  |  | U4052 |  | [X]Pois ?int narcotic pub ins |
|  |  |  | U4053 |  | [X]Pois ?int narcotic sport ar |
|  |  |  | U4054 |  | [X]Pois ?int narcotic on hway |
|  |  |  | U4055 |  | [X]Pois ?int narcotic trade ar |
|  |  |  | U4056 |  | [X]Pois ?int narcotic indus ar |
|  |  |  | U4057 |  | [X]Pois ?int narcotic on farm |
|  |  |  | U405y |  | [X]Pois ?int narcotic OS place |
|  |  |  | U405z |  | [X]Pois ?int narcotic unsp pl |
|  |  |  | U406. |  | [X]Poison ?intent hallucinogen |
|  |  |  | U4060 |  | [X]Pois ?intent hallucin home |
|  |  |  | U4061 |  | [X]Pois ?int hallucinog resid |
|  |  |  | U4062 |  | [X]Pois ?int hallucin pub ins |
|  |  |  | U4063 |  | [X]Pois ?int hallucin sport ar |
|  |  |  | U4064 |  | [X]Pois ?int hallucin on hway |
|  |  |  | U4065 |  | [X]Pois ?int hallucin trade ar |
|  |  |  | U4066 |  | [X]Pois ?int hallucin indus ar |
|  |  |  | U4067 |  | [X]Pois ?int hallucin on farm |
|  |  |  | U406y |  | [X]Pois ?int hallucin OS place |
|  |  |  | U406z |  | [X]Pois ?int hallucin unsp pl |
|  | Y15, Y151-Y159 |  | U409. |  | [X]Poisoning ?intent alcohol |
|  |  |  | U4090 |  | [X]Poison ?intent alcohol home |
|  |  |  | U4091 |  | [X]Pois ?int alcohol res ins |
|  |  |  | U4092 |  | [X]Pois ?int alcohol pub inst |
|  |  |  | U4093 |  | [X]Pois ?int alcohol sport ar |
|  |  |  | U4094 |  | [X]Pois ?int alcohol on hway |
|  |  |  | U4095 |  | [X]Pois ?int alcohol trade ar |
|  |  |  | U4096 |  | [X]Pois ?int alcohol indust ar |
|  |  |  | U4097 |  | [X]Pois ?intent alcoh on farm |
|  |  |  | U409y |  | [X]Pois ?int alcohol OS place |
|  |  |  | U409z |  | [X]Pois ?int alcohol unsp plc |
|  | Y573 |  | U60H3 |  | [X]Alcohol deterrents adv eff |
|  | Y904 |  | U804. |  | [X]Evid alc inv,80-99mg/100ml |
|  | Y905 |  | U805. |  | [X]Ev alc inv,100-119mg/100ml |
|  | Y906 |  | U806. |  | [X]Ev alc inv,120-199mg/100ml |
|  | Y907 |  | U807. |  | [X]Ev alc inv,200-239mg/100ml |
|  | Y908 |  | U808. |  | [X]Evid alc inv,240mg/100ml+ |
|  | Y912 |  | U812. |  | [X]Evid alc inv,severe intoxic |
|  | Y913 |  | U813. |  | [X]Evid alc inv,very sev intox |
|  |  |  | ZV113 |  | [V]PH - Alcoholism |
|  |  |  | ZV114 |  | [V]Pers hist subst abuse |
|  | Z722 |  | ZV4K1 |  | [V]Drug use |
|  | Z721 |  |  |  | Alcohol use |
|  | Z502 |  | ZV57A |  | [V]Alcohol rehabilitation |
|  | Z714 |  | ZV6D6 |  | [V]Alcohl abus counsel+surveil |
|  | Z715 |  | ZV6D7 |  | [V]Drug abuse counsel+surveiln |

**Abbreviations:** ADHD; Attention Deficit Hyperactive Disorder, NEC; Not Elsewhere Classified, NOS; Not Otherwise Specified, O/E; On Examination, OS; Other Specified
