# Supplementary material for: Longitudinal analysis of the relationship between motor and psychiatric symptoms in idiopathic dystonia
Source: Eur J Neurol. 2022 Sep 11;29(12):3513–27. doi: 10.1111/ene.15530 (PMC9826317; doi:10.1111/ene.15530)
Supplement: Supplementary file 6 — TABLE S4 [file ENE-29-3513-s006.docx]

**Supplementary Table 4: Medication codes used to identify prescription of antidepressants, anxiolytics and hypnotics (packet/bottle level)**

| **Drug** | **Read Code** | **Description** |
| --- | --- | --- |
| Antidepressants |  |  |
|  | d71.. | Amitriptyline hydrochloride |
|  | d72.. | Butriptyline - discontinued |
|  | d73.. | Clomipramine hydrochloride |
|  | d74.. | Desipramine hydrochloride |
|  | d75.. | Dosulepin Hydrochloride |
|  | d76.. | Doxepin |
|  | d77.. | Imipramine hydrochloride |
|  | d78.. | Iprindole |
|  | d79.. | Lofepramine |
|  | d7a.. | Maprotiline hydrochloride |
|  | d7b.. | Mianserin hydrochloride |
|  | d7c.. | Nortriptyline |
|  | d7d.. | Protriptyline hydrochloride |
|  | d7e.. | Trazadone hydrochloride |
|  | d7f.. | Trimipramine |
|  | d7g.. | Viloxazine hydrochloride |
|  | d7h.. | Amoxapine |
|  | d81.. | Phenelzine |
|  | d83.. | Isocarboxazid |
|  | d84.. | Tranylcypromine |
|  | d85.. | Moclobemide |
|  | d91.. | Compound Antidepressants A-Z |
|  | da1.. | Flupentixol [Antidepressant] |
|  | da2.. | Tryptophan |
|  | da3.. | Fluvoxamine Maleate |
|  | da4.. | Fluoxetine hydrochloride |
|  | da5.. | Sertraline hydrochloride |
|  | da6.. | Paroxetine hydrochloride |
|  | da7.. | Venlafaxine |
|  | da9.. | Citalopram |
|  | daA.. | Reboxetine |
|  | daB.. | Mirtazapine |
|  | daC.. | Escitalopram |
|  | daD.. | Agomelatine |
|  | gde.. | Duloxetine |
| Hypnotics |  |  |
|  | d11.. | Chloral hydrate |
|  | d12.. | Clomethiazole edisylate (hypnotic) |
|  | d13.. | Dichloralphenazone - discontinued |
|  | d14.. | Flumtrazepam - discontinued |
|  | d15.. | Flurazepam |
|  | d16.. | Loprazolam |
|  | d17.. | Lormetazepam |
|  | d18.. | Nitrazepam |
|  | d1a.. | Temazepam (hynotic) |
|  | d1b.. | Triazolam - discontinued |
|  | d1c.. | Triclofos sodium |
|  | d1d.. | Zopiclone |
|  | d1f.. | Zolpidem |
|  | d1g.. | Zaleplon |
|  | d1h.. | Melatonin |
|  | d1i.. | Dexmedetomidine |
| Anxiolytics |  |  |
|  | d21.. | Diazepam |
|  | d22.. | Alprazolam |
|  | d23.. | Bromazepam |
|  | d24.. | Chlordiazepoxide |
|  | d25.. | Chlormezanone |
|  | d26.. | Clobazam |
|  | d27.. | Clorazepate dipotassium |
|  | d28.. | Hydroxyzine hcl (anxiolytic) |
|  | d29.. | Ketazolam - discontinued |
|  | d2a.. | Lorazepam (anxiolytic) |
|  | d2b.. | Medazepam - discontinued |
|  | d2c.. | Meprobamate |
|  | d2d.. | Oxazepam |
|  | d2f.. | Buspirone hydrocholoride |
|  | d2g.. | Flumazenil |
| Antipsychotics |  |  |
|  | d4… | ANTIPSYCHOTIC DRUGS |
|  | d4f.. | SULPIRIDE |
|  | d4f1. | DOLMATIL 200mg tablets |
|  | d4f2. | *SULPITIL 200mg tablets x28CP |
|  | d4f3. | *SULPITIL 200mg tablets x112CP |
|  | d4f4. | *SULPAREX 200mg tablets |
|  | d4f5. | DOLMATIL 400mg tablets |
|  | d4f6. | SULPOR 200mg/5mL oral solution |
|  | d4fw. | SULPIRIDE 200mg/5mL oral solution |
|  | d4fx. | SULPIRIDE 400mg tablets |
|  | d4fy. | SULPIRIDE 200mg/5mL sugar free solution |
|  | d4fz. | SULPIRIDE 200mg tablets |
|  | d41.. | CHLORPROMAZINE HYDROCHLORIDE |
|  | d411. | CHLORPROMAZINE 10mg tablets |
|  | d412. | CHLORPROMAZINE 25mg tablets |
|  | d413. | CHLORPROMAZINE 50mg tablets |
|  | d414. | CHLORPROMAZINE 100mg tablets |
|  | d415. | CHLORPROMAZINE 25mg/5mL syrup |
|  | d416. | CHLORACTIL 25mg tablets |
|  | d417. | CHLORACTIL 50mg tablets |
|  | d418. | CHLORACTIL 100mg tablets |
|  | d419. | *DOZINE 25mg/5mL syrup |
|  | d41A. | CHLORPROMAZINE 25mg/5mL sugar free solution |
|  | d41B. | CHLORPROMAZINE 100mg/5mL sugar free solution |
|  | d41a. | *LARGACTIL 10mg tablets |
|  | d41b. | *LARGACTIL 25mg tablets |
|  | d41c. | *LARGACTIL 50mg tablets |
|  | d41d. | *LARGACTIL 100mg tablets |
|  | d41e. | *LARGACTIL 25mg/5mL syrup |
|  | d41f. | LARGACTIL FORTE 100mg/5mL syrup |
|  | d41g. | *LARGACTIL 25mg/mL injection |
|  | d41h. | LARGACTIL [CNS] 50mg/2mL injection |
|  | d41i. | *LARGACTIL 100mg suppositories |
|  | d41j. | CHLORPROMAZINE 100mg/5mL sugar free suspension |
|  | d41k. | CHLORPROMAZINE 100mg suppositories |
|  | d41l. | CHLORPROMAZINE 25mg/1mL injection |
|  | d41m. | CHLORPROMAZINE 50mg/2mL injection |
|  | d41o. | CHLORPROMAZINE 100mg/5mL syrup |
|  | d42.. | BENPERIDOL |
|  | d421. | ANQUIL 250micrograms tablets |
|  | d422. | *BENQUIL 250micrograms tablets |
|  | d42z. | BENPERIDOL 250microgram tablets |
|  | d43.. | *CHLORPROTHIXENE |
|  | d431. | *TARACTAN 15mg tablets |
|  | d432. | *TARACTAN 50mg tablets |
|  | d43y. | *CHLORPROTHIXENE 15mg tablets |
|  | d43z. | *CHLORPROTHIXENE 50mg tablets |
|  | d44.. | DROPERIDOL [CENTRAL NERVOUS SYSTEM USE] |
|  | d441. | *DROLEPTAN 10mg tablets |
|  | d442. | *DROLEPTAN 1mg/mL oral liquid |
|  | d443. | *DROLEPTAN 10mg/2mL injection |
|  | d444. | XOMOLIX 2.5mg/1mL solution for injection |
|  | d44w. | DROPERIDOL 2.5mg/1mL solution for injection |
|  | d44x. | *DROPERIDOL 10mg tablets |
|  | d44y. | *DROPERIDOL 1mg/mL oral liquid |
|  | d44z. | *DROPERIDOL 10mg/2mL injection |
|  | d45.. | FLUPENTIXOL [ANTIPSYCHOTIC] |
|  | d451. | DEPIXOL 3mg tablets |
|  | d45z. | FLUPENTIXOL 3mg tablets |
|  | d46.. | FLUPHENAZINE HYDROCHLORIDE |
|  | d461. | *MODITEN 1mg tablets |
|  | d462. | *MODITEN 2.5mg tablets |
|  | d463. | *MODITEN 5mg tablets |
|  | d46x. | FLUPHENAZINE HYDROCHLORIDE 1mg tablets |
|  | d46y. | FLUPHENAZINE HYDROCHLORIDE 2.5mg tablets |
|  | d46z. | FLUPHENAZINE HYDROCHLORIDE 5mg tablets |
|  | d47.. | HALOPERIDOL [ANTIPSYCHOTIC] |
|  | d471. | HALOPERIDOL 1.5mg tablets |
|  | d472. | HALOPERIDOL 5mg tablets |
|  | d473. | HALOPERIDOL 10mg tablets |
|  | d474. | HALOPERIDOL 20mg tablets |
|  | d475. | HALOPERIDOL 2mg/mL liquid |
|  | d476. | DOZIC 1mg/mL liquid |
|  | d477. | *DOZIC 2mg/mL liquid |
|  | d478. | FORTUNAN 500micrograms tablets |
|  | d479. | *FORTUNAN 1.5mg tablets |
|  | d47A. | HALOPERIDOL 2mg/5mL sugar free solution |
|  | d47B. | HALOPERIDOL 1mg/5mL sugar free solution |
|  | d47C. | KENTACE 1.5mg tablets |
|  | d47D. | KENTACE 5mg tablets |
|  | d47E. | KENTACE 10mg tablets |
|  | d47F. | KENTACE 20mg tablets |
|  | d47a. | *FORTUNAN 5mg tablets |
|  | d47b. | *FORTUNAN 10mg tablets |
|  | d47c. | *FORTUNAN 20mg tablets |
|  | d47d. | HALDOL 5mg tablets |
|  | d47e. | HALDOL 10mg tablets |
|  | d47f. | HALDOL 2mg/mL liquid |
|  | d47g. | *HALDOL 10mg/mL liquid |
|  | d47h. | HALDOL 5mg/1mL injection |
|  | d47i. | *HALDOL 10mg/2mL injection |
|  | d47j. | SERENACE 500micrograms capsules |
|  | d47k. | SERENACE 1.5mg tablets |
|  | d47l. | SERENACE 5mg tablets |
|  | d47m. | SERENACE 10mg tablets |
|  | d47n. | SERENACE 20mg tablets |
|  | d47o. | SERENACE 2mg/mL liquid 100mL |
|  | d47p. | SERENACE 5mg/1mL injection |
|  | d47q. | SERENACE 20mg/2mL injection |
|  | d47r. | HALOPERIDOL 500microgram capsules |
|  | d47s. | SERENACE 2mg/mL liquid 500mL |
|  | d47t. | HALOPERIDOL 1mg/mL liquid |
|  | d47u. | HALOPERIDOL 500micrograms tablets |
|  | d47v. | HALOPERIDOL 5mg/1mL injection |
|  | d47w. | HALOPERIDOL 10mg/2mL injection |
|  | d47x. | HALOPERIDOL 20mg/2mL injection |
|  | d47y. | HALOPERIDOL 10mg/mL oral solution |
|  | d48.. | LEVOMEPROMAZINE |
|  | d481. | NOZINAN 25mg/1mL injection |
|  | d482. | *VERACTIL 25mg tablets |
|  | d483. | NOZINAN 25mg tablets |
|  | d48y. | LEVOMEPROMAZINE 25mg/1mL injection |
|  | d48z. | LEVOMEPROMAZINE 25mg tablets |
|  | d49.. | OXYPERTINE |
|  | d491. | *INTEGRIN 10mg capsules |
|  | d492. | *INTEGRIN 40mg tablets |
|  | d49y. | *OXYPERTINE 10mg capsules |
|  | d49z. | *OXYPERTINE 40mg tablets |
|  | d4a.. | PERICYAZINE |
|  | d4a1. | *NEULACTIL 2.5mg tablets |
|  | d4a2. | *NEULACTIL 10mg tablets |
|  | d4a3. | *NEULACTIL 25mg tablets |
|  | d4a4. | *NEULACTIL FORTE 10mg/5mL syrp |
|  | d4aw. | PERICYAZINE 2.5mg tablets |
|  | d4ax. | PERICYAZINE 10mg tablets |
|  | d4ay. | *PERICYAZINE 25mg tablets |
|  | d4az. | PERICYAZINE 10mg/5mL syrup |
|  | d4b.. | PERPHENAZINE [CENTRAL NERVOUS SYSTEM USE] |
|  | d4b1. | FENTAZIN 2mg tablets |
|  | d4b2. | FENTAZIN 4mg tablets |
|  | d4b3. | *FENTAZIN 8mg tablets |
|  | d4b4. | *FENTAZIN 5mg/1mL injection |
|  | d4b5. | PERPHENAZINE 2mg/5mL sugar free solution |
|  | d4b6. | PERPHENAZINE 4mg/5mL sugar free solution |
|  | d4bx. | PERPHENAZINE 2mg tablets |
|  | d4by. | PERPHENAZINE 4mg tablets |
|  | d4bz. | *PERPHENAZINE 8mg tablets |
|  | d4c.. | PIMOZIDE |
|  | d4c1. | *ORAP 2mg tablets |
|  | d4c2. | ORAP 4mg tablets |
|  | d4c3. | *ORAP 10mg tablets |
|  | d4cx. | *PIMOZIDE 2mg tablets |
|  | d4cy. | PIMOZIDE 4mg tablets |
|  | d4cz. | *PIMOZIDE 10mg tablets |
|  | d4d.. | PROCHLORPERAZINE [antipsych] [see dhe..] |
|  | d4e.. | PROMAZINE HYDROCHLORIDE |
|  | d4e1. | *SPARINE 50mg/5mL suspension |
|  | d4e2. | *SPARINE 50mg/1mL injection |
|  | d4e3. | *SPARINE 100mg/2mL injection |
|  | d4e4. | PROMAZINE 25mg tablets |
|  | d4e5. | PROMAZINE 50mg tablets |
|  | d4ev. | PROMAZINE 25mg/5mL syrup |
|  | d4ew. | PROMAZINE 50mg/5mL syrup |
|  | d4ex. | *PROMAZINE 50mg/5mL suspension |
|  | d4ey. | PROMAZINE 50mg/1mL injection |
|  | d4ez. | *PROMAZINE 100mg/2mL injection |
|  | d4g.. | THIORIDAZINE |
|  | d4g1. | *MELLERIL 10mg tablets |
|  | d4g2. | *MELLERIL 25mg tablets |
|  | d4g3. | *MELLERIL 50mg tablets |
|  | d4g4. | *MELLERIL 100mg tablets |
|  | d4g5. | *MELLERIL 25mg/5mL suspension |
|  | d4g6. | MELLERIL 100mg/5mL oral suspension |
|  | d4g7. | MELLERIL 25mg/5mL orange syrup |
|  | d4gp. | THIORIDAZINE 10mg/5mL syrup |
|  | d4gq. | THIORIDAZINE 25mg/5mL sugar free solution |
|  | d4gr. | THIORIDAZINE 50mg/5mL sugar free solution |
|  | d4gs. | THIORIDAZINE 100mg/5mL sugar free solution |
|  | d4gt. | *THIORIDAZINE 10mg tablets |
|  | d4gu. | THIORIDAZINE 25mg tablets |
|  | d4gv. | THIORIDAZINE 50mg tablets |
|  | d4gw. | THIORIDAZINE 100mg tablets |
|  | d4gx. | THIORIDAZINE 25mg/5mL suspension |
|  | d4gy. | THIORIDAZINE 100mg/5mL oral suspension |
|  | d4gz. | *THIORIDAZINE 25mg/5mL syrup |
|  | d4h.. | TRIFLUOPERAZINE [ANTIPSYCHOTIC] |
|  | d4h1. | STELAZINE 1mg tablets |
|  | d4h2. | STELAZINE 5mg tablets |
|  | d4h3. | *STELAZINE 2mg m/r capsules |
|  | d4h4. | *STELAZINE 10mg m/r capsules |
|  | d4h5. | *STELAZINE 15mg m/r capsules |
|  | d4h6. | STELAZINE 1mg/5mL syrup |
|  | d4h7. | STELAZINE CONCENTRATE 10mg/mL liquid |
|  | d4h8. | *STELAZINE 1mg/1mL injection |
|  | d4h9. | TRIFLUOPERAZINE 5mg/5mL sugar free syrup |
|  | d4hA. | STELAZINE FORTE 5mg/5mL sugar free oral suspension |
|  | d4hr. | TRIFLUOPERAZINE 5mg/5mL sugar free oral suspension |
|  | d4hs. | TRIFLUOPERAZINE 1mg tablets |
|  | d4ht. | TRIFLUOPERAZINE 5mg tablets |
|  | d4hu. | *TRIFLUOPERAZINE 2mg m/r caps |
|  | d4hv. | *TRIFLUOPERAZINE 10mg m/r caps |
|  | d4hw. | *TRIFLUOPERAZINE 15mg m/r caps |
|  | d4hx. | TRIFLUOPERAZINE 1mg/5mL syrup |
|  | d4hy. | TRIFLUOPERAZINE 10mg/mL liquid |
|  | d4hz. | TRIFLUOPERAZINE 1mg/1mL injection |
|  | d4i1. | TRIFLUPERIDOL |
|  | d4i2. | TRIPERIDOL 500micrograms tablets |
|  | d4iy. | *TRIPERIDOL 1mg tablets |
|  | d4iz. | TRIFLUPERIDOL 500microgram tablets |
|  | d4l.. | *TRIFLUPERIDOL 1mg tablets |
|  | d4j.. | ZUCLOPENTHIXOL DIHYDROCHLORIDE |
|  | d4j1. | CLOPIXOL 2mg tablets |
|  | d4j2. | CLOPIXOL 10mg tablets |
|  | d4j3. | CLOPIXOL 25mg tablets |
|  | d4jx. | ZUCLOPENTHIXOL DIHYDROCHLORIDE 2mg tablets |
|  | d4jy. | ZUCLOPENTHIXOL DIHYDROCHLORIDE 10mg tablets |
|  | d4jz. | ZUCLOPENTHIXOL DIHYDROCHLORIDE 25mg tablets |
|  | d4k.. | LOXAPINE SUCCINATE |
|  | d4k1. | *LOXAPINE 10mg capsules |
|  | d4k2. | *LOXAPINE 25mg capsules |
|  | d4k3. | *LOXAPINE 50mg capsules |
|  | d4k4. | *LOXAPAC 10mg capsules |
|  | d4k5. | *LOXAPAC 25mg capsules |
|  | d4k6. | *LOXAPAC 50mg capsules |
|  | d4n.. | ZUCLOPENTHIXOL ACETATE |
|  | d4n1. | CLOPIXOL ACUPHASE 50mg/1mL injection (oily) |
|  | d4n2. | CLOPIXOL ACUPHASE 100mg/2mL injection (oily) |
|  | d4n3. | ZUCLOPENTHIXOL ACETATE 50mg/1mL injection (oily) |
|  | d4n4. | ZUCLOPENTHIXOL ACETATE 100mg/2mL injection (oily) |
|  | d5... | ANTIPSYCHOTIC DEPOT INJECTIONS |
|  | d51.. | FLUPENTIXOL DECANOATE |
|  | d511. | DEPIXOL 20mg/1mL injection |
|  | d512. | *DEPIXOL 20mg/1mL syringe |
|  | d513. | DEPIXOL 40mg/2mL injection |
|  | d514. | *DEPIXOL 40mg/2mL syringe |
|  | d515. | *DEPIXOL 200mg/10mL injection |
|  | d516. | DEPIXOL CONC. 100mg/1mL injection |
|  | d517. | DEPIXOL CONC. 500mg/5mL injection |
|  | d518. | DEPIXOL CONC. 50mg/0.5mL injection |
|  | d519. | FLUPENTIXOL 50mg/0.5mL injection |
|  | d51a. | DEPIXOL LOW VOLUME 200mg/1mL intramuscular injection |
|  | d51s. | FLUPENTHIXOL DECANOATE 20mg/1mL prefilled syringe |
|  | d51t. | FLUPENTHIXOL DECANOATE 40mg/2mL prefilled syringe |
|  | d51u. | FLUPENTIXOL DECANOATE 200mg/1mL intramuscular injection |
|  | d51v. | FLUPENTIXOL DECANOATE 20mg/1mL injection |
|  | d51w. | FLUPENTIXOL DECANOATE 40mg/2mL injection |
|  | d51x. | FLUPENTHIXOL DECANOATE 200mg/10mL injection |
|  | d51y. | FLUPENTIXOL DECANOATE 100mg/1mL injection |
|  | d51z. | FLUPENTHIXOL DECANOATE 500mg/5mL injection |
|  | d52.. | FLUPHENAZINE DECANOATE |
|  | d521. | MODECATE 12.5mg/0.5mL injection |
|  | d522. | MODECATE 25mg/1mL injection |
|  | d523. | *MODECATE 25mg/1mL syringe |
|  | d524. | MODECATE 50mg/2mL injection |
|  | d525. | *MODECATE 50mg/2mL syringe |
|  | d526. | *MODECATE 250mg/10mL injection |
|  | d527. | MODECATE CONCENTRATE 50mg/0.5mL injection |
|  | d528. | MODECATE CONCENTRATE 100mg/1mL injection |
|  | d529. | FLUPHENAZINE DECANOATE 50mg/0.5mL injection |
|  | d52A. | *DECAZATE 25mg/1mL injection |
|  | d52B. | *DECAZATE 50mg/0.5mL injection |
|  | d52C. | *DECAZATE 100mg/1mL injection |
|  | d52a. | FLUPHENAZINE DECANOATE 100mg/1mL injection |
|  | d52s. | FLUPHENAZINE DECANOATE 25mg/1mL prefilled syringe |
|  | d52t. | FLUPHENAZINE DECANOATE 50mg/2mL prefilled syringe |
|  | d52u. | FLUPHENAZINE DECANOATE 12.5mg/0.5mL injection |
|  | d52v. | FLUPHENAZINE DECANOATE 25mg/1mL injection |
|  | d52w. | FLUPHENAZINE DECANOATE 50mg/2mL injection |
|  | d52x. | FLUPHENAZINE DECANOATE 250mg/10mL injection |
|  | d53.. | *FLUPHENAZINE ENANTHATE |
|  | d531. | MODITEN ENANTHATE 25mg/1mL injection |
|  | d532. | FLUPHENAZINE ENANTHATE 25mg/1mL injection |
|  | d54.. | FLUSPIRILENE |
|  | d541. | *REDEPTIN 2mg/1mL injection |
|  | d542. | *REDEPTIN 6mg/3mL injection |
|  | d543. | *REDEPTIN 12mg/6mL injection |
|  | d544. | FLUSPIRILENE 2mg/1mL injection |
|  | d545. | FLUSPIRILENE 6mg/3mL injection |
|  | d546. | FLUSPIRILENE 12mg/6mL injection |
|  | d55.. | HALOPERIDOL DECANOATE |
|  | d551. | HALDOL DECANOATE 50mg/1mL injection |
|  | d552. | HALDOL DECANOATE 100mg/1mL injection |
|  | d553. | HALOPERIDOL 50mg/1mL injection |
|  | d554. | HALOPERIDOL 100mg/1mL injection |
|  | d56.. | PIPOTIAZINE PALMITATE |
|  | d561. | PIPORTIL DEPOT 50mg/1mL injection |
|  | d562. | PIPORTIL DEPOT 100mg/2mL injection |
|  | d563. | PIPOTIAZINE 50mg/1mL injection |
|  | d564. | PIPOTIAZINE 100mg/2mL injection |
|  | d57.. | ZUCLOPENTHIXOL DECANOATE |
|  | d571. | CLOPIXOL 200mg/1mL injection |
|  | d572. | *CLOPIXOL 2g/10mL injection |
|  | d573. | CLOPIXOL CONC. 500mg/1mL injection |
|  | d574. | CLOPIXOL ACUPHASE 50mg/1mL injection (oily) |
|  | d575. | CLOPIXOL ACUPHASE 100mg/2mL injection (oily) |
|  | d576. | ZUCLOPENTHIXOL DECANOATE 200mg/1mL injection |
|  | d577. | ZUCLOPENTHIXOL DECANOATE 50mg/1mL injection |
|  | d578. | ZUCLOPENTHIXOL DECANOATE 100mg/2mL injection |
|  | d57y. | ZUCLOPENTHIXOL DECANOATE 2g/10mL injection |
|  | d57z. | ZUCLOPENTHIXOL DECANOATE 500mg/1mL injection |
|  | d4l.. | CLOZAPINE |
|  | d4l1. | CLOZAPINE 25mg tablets |
|  | d4l2. | CLOZAPINE 100mg tablets |
|  | d4l3. | CLOZARIL 25mg tablets x84CP |
|  | d4l4. | CLOZARIL 100mg tablets x84CP |
|  | d4l5. | CLOZARIL COMMUNITY PACK 25mg tablets x28CP |
|  | d4l6. | CLOZARIL COMMUNITY PACK 100mg tablets x28CP |
|  | d4l7. | DENZAPINE 25mg tablets |
|  | d4l8. | DENZAPINE 100mg tablets |
|  | d4l9. | ZAPONEX 25mg tablets |
|  | d4lA. | ZAPONEX 100mg tablets |
|  | d4lB. | DENZAPINE 50mg/mL oral suspension 100mL |
|  | d4lC. | CLOZAPINE 50mg/mL oral suspension |
|  | d4lD. | DENZAPINE 50mg tablets |
|  | d4lE. | CLOZAPINE 50mg tablets |
|  | d4lF. | DENZAPINE 200mg tablets |
|  | d4lG. | CLOZAPINE 200mg tablets |
|  | d4m.. | REMOXIPRIDE |
|  | d4m1. | REMOXIPRIDE 150mg m/r capsules |
|  | d4m2. | REMOXIPRIDE 300mg m/r capsules |
|  | d4m3. | *ROXIAM 150mg m/r capsules |
|  | d4m4. | *ROXIAM 300mg m/r capsules |
|  | d4p.. | RISPERIDONE |
|  | d4p1. | RISPERIDONE 1mg tablets |
|  | d4p2. | RISPERIDONE 2mg tablets |
|  | d4p3. | RISPERIDONE 3mg tablets |
|  | d4p4. | RISPERIDONE 4mg tablets |
|  | d4p5. | RISPERDAL 1mg tablets |
|  | d4p6. | RISPERDAL 2mg tablets |
|  | d4p7. | RISPERDAL 3mg tablets |
|  | d4p8. | RISPERDAL 4mg tablets |
|  | d4p9. | RISPERIDONE 1mg/mL liquid |
|  | d4pA. | RISPERDAL 1mg/mL liquid |
|  | d4pB. | RISPERIDONE 6mg tablets |
|  | d4pC. | RISPERDAL 6mg tablets |
|  | d4pD. | RISPERDAL 0.5mg tablets |
|  | d4pE. | RISPERDAL CONSTA 25mg powder+solvent for suspension for injection |
|  | d4pF. | RISPERDAL CONSTA 37.5mg powder+solvent for suspension for injection |
|  | d4pG. | RISPERDAL CONSTA 50mg powder+solvent for suspension for injection |
|  | d4pH. | RISPERIDONE 1mg oro-dispersible tablets |
|  | d4pJ. | RISPERIDONE 2mg oro-dispersible tablets |
|  | d4pK. | RISPERDAL QUICKLET 1mg oro-dispersible tablets |
|  | d4pL. | RISPERDAL QUICKLET 2mg oro-dispersible tablets |
|  | d4pM. | RISPERIDONE 0.5mg oro-dispersible tablets |
|  | d4pN. | RISPERDAL QUICKLET 0.5mg oro-dispersible tablets |
|  | d4pO. | RISPERDAL QUICKLET 3mg oro-dispersible tablets |
|  | d4pP. | RISPERDAL QUICKLET 4mg oro-dispersible tablets |
|  | d4pQ. | RISPERIDONE 3mg oro-dispersible tablets |
|  | d4pR. | RISPERIDONE 4mg oro-dispersible tablets |
|  | d4pw. | RISPERIDONE 50mg powder+solvent for suspension for injection |
|  | d4px. | RISPERIDONE 37.5mg powder+solvent for suspension for injection |
|  | d4py. | RISPERIDONE 25mg powder+solvent for suspension for injection |
|  | d4pz. | RISPERIDONE 0.5mg tablets |
|  | d4q.. | SERTINDOLE |
|  | d4q1. | SERTINDOLE 4mg tablets |
|  | d4q2. | SERTINDOLE 12mg tablets |
|  | d4q3. | SERTINDOLE 16mg tablets |
|  | d4q4. | SERTINDOLE 20mg tablets |
|  | d4q5. | SERDOLECT 4mg tablets |
|  | d4q6. | SERDOLECT 12mg tablets |
|  | d4q7. | SERDOLECT 16mg tablets |
|  | d4q8. | SERDOLECT 20mg tablets |
|  | d4r.. | OLANZAPINE |
|  | d4r1. | OLANZAPINE 5mg tablets |
|  | d4r2. | OLANZAPINE 7.5mg tablets |
|  | d4r3. | OLANZAPINE 10mg tablets |
|  | d4r4. | ZYPREXA 5mg tablets |
|  | d4r5. | ZYPREXA 7.5mg tablets |
|  | d4r6. | ZYPREXA 10mg tablets |
|  | d4r7. | OLANZAPINE 2.5mg tablets |
|  | d4r8. | ZYPREXA 2.5mg tablets |
|  | d4r9. | ZYPREXA VELOTAB 5mg dispersible tablets |
|  | d4rA. | ZYPREXA VELOTAB 10mg dispersible tablets |
|  | d4rB. | ZYPREXA 15mg tablets |
|  | d4rC. | ZYPREXA VELOTAB 15mg dispersible tablets |
|  | d4rD. | ZYPREXA 10mg injection (pdr for recon) |
|  | d4rE. | ZYPREXA VELOTAB 20mg dispersible tablets |
|  | d4rF. | ZYPREXA 20mg tablets |
|  | d4rG. | ZALASTA 2.5mg tablets |
|  | d4rH. | ZALASTA 5mg tablets |
|  | d4rI. | ZALASTA 7.5mg tablets |
|  | d4rJ. | ZALASTA 15mg tablets |
|  | d4rK. | ZALASTA 20mg tablets |
|  | d4rL. | ZALASTA 5mg dispersible tablets |
|  | d4rM. | ZALASTA 10mg dispersible tablets |
|  | d4rN. | ZALASTA 15mg dispersible tablets |
|  | d4rO. | ZALASTA 20mg dispersible tablets |
|  | d4rP. | ZALASTA 10mg tablets |
|  | d4rt. | OLANZAPINE 20mg tablets |
|  | d4ru. | OLANZAPINE 20mg dispersible tablets |
|  | d4rv. | OLANZAPINE 10mg injection (pdr for recon) |
|  | d4rw. | OLANZAPINE 15mg dispersible tablets |
|  | d4rx. | OLANZAPINE 15mg tablets |
|  | d4ry. | OLANZAPINE 5mg dispersible tablets |
|  | d4rz. | OLANZAPINE 10mg dispersible tablets |
|  | d4s.. | QUETIAPINE |
|  | d4s1. | QUETIAPINE 25mg tablets |
|  | d4s2. | QUETIAPINE 100mg tablets |
|  | d4s3. | QUETIAPINE 200mg tablets |
|  | d4s4. | QUETIAPINE 25mg+100mg tablets starter pack |
|  | d4s5. | SEROQUEL 25mg tablets |
|  | d4s6. | SEROQUEL 100mg tablets |
|  | d4s7. | SEROQUEL 200mg tablets |
|  | d4s8. | SEROQUEL 25mg+100mg tablets starter pack |
|  | d4s9. | SEROQUEL 150mg tablets |
|  | d4sA. | SEROQUEL 25mg+100mg+150mg tablets starter pack |
|  | d4sB. | SEROQUEL 300mg tablets |
|  | d4sC. | SEROQUEL XL 50mg m/r tablets |
|  | d4sD. | SEROQUEL XL 200mg m/r tablets |
|  | d4sE. | SEROQUEL XL 300mg m/r tablets |
|  | d4sF. | SEROQUEL XL 400mg m/r tablets |
|  | d4sG. | SEROQUEL XL 150mg m/r tablets |
|  | d4ss. | QUETIAPINE 150mg m/r tablets |
|  | d4st. | QUETIAPINE 400mg m/r tablets |
|  | d4su. | QUETIAPINE 300mg m/r tablets |
|  | d4sv. | QUETIAPINE 200mg m/r tablets |
|  | d4sw. | QUETIAPINE 50mg m/r tablets |
|  | d4sx. | QUETIAPINE 300mg tablets |
|  | d4sy. | QUETIAPINE 25mg+100mg+150mg tablets starter pack |
|  | d4sz. | QUETIAPINE 150mg tablets |
|  | d4t.. | AMISULPRIDE |
|  | d4t1. | AMISULPRIDE 50mg tablets |
|  | d4t2. | AMISULPRIDE 200mg tablets |
|  | d4t3. | SOLIAN 50mg tablets |
|  | d4t4. | SOLIAN 200mg tablets |
|  | d4t5. | SOLIAN 400mg tablets |
|  | d4t6. | SOLIAN 100mg/mL sugar free oral solution |
|  | d4t7. | SOLIAN 100mg tablets |
|  | d4tx. | AMISULPRIDE 100mg tablets |
|  | d4ty. | AMISULPRIDE 100mg/mL sugar free oral solution |
|  | d4tz. | AMISULPRIDE 400mg tablets |
|  | d4u.. | ZOTEPINE |
|  | d4u1. | *ZOTEPINE 25mg tablets |
|  | d4u2. | *ZOTEPINE 50mg tablets |
|  | d4u3. | *ZOTEPINE 100mg tablets |
|  | d4u4. | *ZOLEPTIL 25mg tablets |
|  | d4u5. | *ZOLEPTIL 50mg tablets |
|  | d4u6. | *ZOLEPTIL 100mg tablets |
|  | d4v.. | ARIPIPRAZOLE |
|  | d4v1. | ABILIFY 10mg tablets |
|  | d4v2. | ABILIFY 15mg tablets |
|  | d4v3. | ABILIFY 30mg tablets |
|  | d4v4. | ABILIFY 5mg tablets |
|  | d4v5. | ABILIFY 10mg oro-dispersible tablets |
|  | d4v6. | ABILIFY 15mg oro-dispersible tablets |
|  | d4v7. | ABILIFY 1mg/mL oral solution |
|  | d4v8. | ABILIFY 9.75mg/1.3mL solution for injection |
|  | d4vs. | ARIPIPRAZOLE 9.75mg/1.3mL solution for injection |
|  | d4vt. | ARIPIPRAZOLE 1mg/mL oral solution |
|  | d4vu. | ARIPIPRAZOLE 10mg oro-dispersible tablets |
|  | d4vv. | ARIPIPRAZOLE 15mg oro-dispersible tablets |
|  | d4vw. | ARIPIPRAZOLE 5mg tablets |
|  | d4vx. | ARIPIPRAZOLE 30mg tablets |
|  | d4vy. | ARIPIPRAZOLE 15mg tablets |
|  | d4vz. | ARIPIPRAZOLE 10mg tablets |
|  | d4w.. | PALIPERIDONE |
|  | d4w1. | INVEGA 3mg m/r tablets |
|  | d4w2. | INVEGA 6mg m/r tablets |
|  | d4w3. | INVEGA 9mg m/r tablets |
|  | d4w4. | *INVEGA 12mg m/r tablets |
|  | d4w5. | XEPLION 50mg suspension for injection prefilled syringe |
|  | d4w6. | XEPLION 75mg suspension for injection prefilled syringe |
|  | d4w7. | XEPLION 100mg suspension for injection prefilled syringe |
|  | d4w8. | XEPLION 150mg suspension for injection prefilled syringe |
|  | d4ws. | PALIPERIDONE 150mg suspension for injection pfs |
|  | d4wt. | PALIPERIDONE 100mg suspension for injection pfs |
|  | d4wu. | PALIPERIDONE 75mg suspension for injection prefilled syringe |
|  | d4wv. | PALIPERIDONE 50mg suspension for injection prefilled syringe |
|  | d4ww. | *PALIPERIDONE 12mg m/r tablets |
|  | d4wx. | PALIPERIDONE 9mg m/r tablets |
|  | d4wy. | PALIPERIDONE 6mg m/r tablets |
|  | d4wz. | PALIPERIDONE 3mg m/r tablets |
|  | d4x.. | ASENAPINE |
|  | d4x1. | SYCREST 5mg sublingual tablets |
|  | d4x2. | ASENAPINE 5mg sublingual tablets |
|  | d4x3. | SYCREST 10mg sublingual tablets |
|  | d4x4. | ASENAPINE 10mg sublingual tablets |
|  | d58.. | OLANZAPINE PAMOATE |
|  | d581. | ZYPADHERA 210mg powder+solvent for suspension for injection |
|  | d582. | ZYPADHERA 300mg powder+solvent for suspension for injection |
|  | d583. | ZYPADHERA 405mg powder+solvent for suspension for injection |
|  | d58x. | OLANZAPINE 405mg powder+solvent for suspension for injection |
|  | d58y. | OLANZAPINE 300mg powder+solvent for suspension for injection |
|  | d58z. | OLANZAPINE 210mg powder+solvent for suspension for injection |
|  | d4gv. | THIORIDAZINE 50mg tablets |
|  | d4gw. | THIORIDAZINE 100mg tablets |
|  | d4gx. | THIORIDAZINE 25mg/5mL suspension |
|  | d4gy. | THIORIDAZINE 100mg/5mL oral suspension |
|  | d4gz. | *THIORIDAZINE 25mg/5mL syrup |
|  | d4h.. | TRIFLUOPERAZINE [ANTIPSYCHOTIC] |
|  | d4h1. | STELAZINE 1mg tablets |
|  | d4h2. | STELAZINE 5mg tablets |
|  | d4h3. | *STELAZINE 2mg m/r capsules |
|  | d4h4. | *STELAZINE 10mg m/r capsules |
|  | d4h5. | *STELAZINE 15mg m/r capsules |
|  | d4h6. | STELAZINE 1mg/5mL syrup |
|  | d4h7. | STELAZINE CONCENTRATE 10mg/mL liquid |
|  | d4h8. | *STELAZINE 1mg/1mL injection |
|  | d4h9. | TRIFLUOPERAZINE 5mg/5mL sugar free syrup |
|  | d4hA. | STELAZINE FORTE 5mg/5mL sugar free oral suspension |
|  | d4hr. | TRIFLUOPERAZINE 5mg/5mL sugar free oral suspension |
|  | d4hs. | TRIFLUOPERAZINE 1mg tablets |
|  | d4ht. | TRIFLUOPERAZINE 5mg tablets |
|  | d4hu. | *TRIFLUOPERAZINE 2mg m/r caps |
|  | d4hv. | *TRIFLUOPERAZINE 10mg m/r caps |
|  | d4hw. | *TRIFLUOPERAZINE 15mg m/r caps |
|  | d4hx. | TRIFLUOPERAZINE 1mg/5mL syrup |
|  | d4hy. | TRIFLUOPERAZINE 10mg/mL liquid |
|  | d4hz. | TRIFLUOPERAZINE 1mg/1mL injection |
|  | d4i1. | TRIFLUPERIDOL |
|  | d4i2. | TRIPERIDOL 500micrograms tablets |
|  | d4iy. | *TRIPERIDOL 1mg tablets |
|  | d4iz. | TRIFLUPERIDOL 500microgram tablets |
|  | d4l.. | *TRIFLUPERIDOL 1mg tablets |
|  | d4j.. | ZUCLOPENTHIXOL DIHYDROCHLORIDE |
|  | d4j1. | CLOPIXOL 2mg tablets |
|  | d4j2. | CLOPIXOL 10mg tablets |
|  | d4j3. | CLOPIXOL 25mg tablets |
|  | d4jx. | ZUCLOPENTHIXOL DIHYDROCHLORIDE 2mg tablets |
|  | d4jy. | ZUCLOPENTHIXOL DIHYDROCHLORIDE 10mg tablets |
|  | d4jz. | ZUCLOPENTHIXOL DIHYDROCHLORIDE 25mg tablets |
|  | d4k.. | LOXAPINE SUCCINATE |
|  | d4k1. | *LOXAPINE 10mg capsules |
|  | d4k2. | *LOXAPINE 25mg capsules |
|  | d4k3. | *LOXAPINE 50mg capsules |
|  | d4k4. | *LOXAPAC 10mg capsules |
|  | d4k5. | *LOXAPAC 25mg capsules |
|  | d4k6. | *LOXAPAC 50mg capsules |
|  | d4n.. | ZUCLOPENTHIXOL ACETATE |
|  | d4n1. | CLOPIXOL ACUPHASE 50mg/1mL injection (oily) |
|  | d4n2. | CLOPIXOL ACUPHASE 100mg/2mL injection (oily) |
|  | d4n3. | ZUCLOPENTHIXOL ACETATE 50mg/1mL injection (oily) |
|  | d4n4. | ZUCLOPENTHIXOL ACETATE 100mg/2mL injection (oily) |
|  | d5... | ANTIPSYCHOTIC DEPOT INJECTIONS |
|  | d51.. | FLUPENTIXOL DECANOATE |
|  | d511. | DEPIXOL 20mg/1mL injection |
|  | d512. | *DEPIXOL 20mg/1mL syringe |
|  | d513. | DEPIXOL 40mg/2mL injection |
|  | d514. | *DEPIXOL 40mg/2mL syringe |
|  | d515. | *DEPIXOL 200mg/10mL injection |
|  | d516. | DEPIXOL CONC. 100mg/1mL injection |
|  | d517. | DEPIXOL CONC. 500mg/5mL injection |
|  | d518. | DEPIXOL CONC. 50mg/0.5mL injection |
|  | d519. | FLUPENTIXOL 50mg/0.5mL injection |
|  | d51a. | DEPIXOL LOW VOLUME 200mg/1mL intramuscular injection |
|  | d51s. | FLUPENTHIXOL DECANOATE 20mg/1mL prefilled syringe |
|  | d51t. | FLUPENTHIXOL DECANOATE 40mg/2mL prefilled syringe |
|  | d51u. | FLUPENTIXOL DECANOATE 200mg/1mL intramuscular injection |
|  | d51v. | FLUPENTIXOL DECANOATE 20mg/1mL injection |
|  | d51w. | FLUPENTIXOL DECANOATE 40mg/2mL injection |
|  | d51x. | FLUPENTHIXOL DECANOATE 200mg/10mL injection |
|  | d51y. | FLUPENTIXOL DECANOATE 100mg/1mL injection |
|  | d51z. | FLUPENTHIXOL DECANOATE 500mg/5mL injection |
|  | d52.. | FLUPHENAZINE DECANOATE |
|  | d521. | MODECATE 12.5mg/0.5mL injection |
|  | d522. | MODECATE 25mg/1mL injection |
|  | d523. | *MODECATE 25mg/1mL syringe |
|  | d524. | MODECATE 50mg/2mL injection |
|  | d525. | *MODECATE 50mg/2mL syringe |
|  | d526. | *MODECATE 250mg/10mL injection |
|  | d527. | MODECATE CONCENTRATE 50mg/0.5mL injection |
|  | d528. | MODECATE CONCENTRATE 100mg/1mL injection |
|  | d529. | FLUPHENAZINE DECANOATE 50mg/0.5mL injection |
|  | d52A. | *DECAZATE 25mg/1mL injection |
|  | d52B. | *DECAZATE 50mg/0.5mL injection |
|  | d52C. | *DECAZATE 100mg/1mL injection |
|  | d52a. | FLUPHENAZINE DECANOATE 100mg/1mL injection |
|  | d52s. | FLUPHENAZINE DECANOATE 25mg/1mL prefilled syringe |
|  | d52t. | FLUPHENAZINE DECANOATE 50mg/2mL prefilled syringe |
|  | d52u. | FLUPHENAZINE DECANOATE 12.5mg/0.5mL injection |
|  | d52v. | FLUPHENAZINE DECANOATE 25mg/1mL injection |
|  | d52w. | FLUPHENAZINE DECANOATE 50mg/2mL injection |
|  | d52x. | FLUPHENAZINE DECANOATE 250mg/10mL injection |
|  | d53.. | *FLUPHENAZINE ENANTHATE |
|  | d531. | MODITEN ENANTHATE 25mg/1mL injection |
|  | d532. | FLUPHENAZINE ENANTHATE 25mg/1mL injection |
|  | d54.. | FLUSPIRILENE |
|  | d541. | *REDEPTIN 2mg/1mL injection |
|  | d542. | *REDEPTIN 6mg/3mL injection |
|  | d543. | *REDEPTIN 12mg/6mL injection |
|  | d544. | FLUSPIRILENE 2mg/1mL injection |
|  | d545. | FLUSPIRILENE 6mg/3mL injection |
|  | d546. | FLUSPIRILENE 12mg/6mL injection |
|  | d55.. | HALOPERIDOL DECANOATE |
|  | d551. | HALDOL DECANOATE 50mg/1mL injection |
|  | d552. | HALDOL DECANOATE 100mg/1mL injection |
|  | d553. | HALOPERIDOL 50mg/1mL injection |
|  | d554. | HALOPERIDOL 100mg/1mL injection |
|  | d56.. | PIPOTIAZINE PALMITATE |
|  | d561. | PIPORTIL DEPOT 50mg/1mL injection |
|  | d562. | PIPORTIL DEPOT 100mg/2mL injection |
|  | d563. | PIPOTIAZINE 50mg/1mL injection |
|  | d564. | PIPOTIAZINE 100mg/2mL injection |
|  | d57.. | ZUCLOPENTHIXOL DECANOATE |
|  | d571. | CLOPIXOL 200mg/1mL injection |
|  | d572. | *CLOPIXOL 2g/10mL injection |
|  | d573. | CLOPIXOL CONC. 500mg/1mL injection |
|  | d574. | CLOPIXOL ACUPHASE 50mg/1mL injection (oily) |
|  | d575. | CLOPIXOL ACUPHASE 100mg/2mL injection (oily) |
|  | d576. | ZUCLOPENTHIXOL DECANOATE 200mg/1mL injection |
|  | d577. | ZUCLOPENTHIXOL DECANOATE 50mg/1mL injection |
|  | d578. | ZUCLOPENTHIXOL DECANOATE 100mg/2mL injection |
|  | d57y. | ZUCLOPENTHIXOL DECANOATE 2g/10mL injection |
|  | d57z. | ZUCLOPENTHIXOL DECANOATE 500mg/1mL injection |
|  | d4l.. | CLOZAPINE |
|  | d4l1. | CLOZAPINE 25mg tablets |
|  | d4l2. | CLOZAPINE 100mg tablets |
|  | d4l3. | CLOZARIL 25mg tablets x84CP |
|  | d4l4. | CLOZARIL 100mg tablets x84CP |
|  | d4l5. | CLOZARIL COMMUNITY PACK 25mg tablets x28CP |
|  | d4l6. | CLOZARIL COMMUNITY PACK 100mg tablets x28CP |
|  | d4l7. | DENZAPINE 25mg tablets |
|  | d4l8. | DENZAPINE 100mg tablets |
|  | d4l9. | ZAPONEX 25mg tablets |
|  | d4lA. | ZAPONEX 100mg tablets |
|  | d4lB. | DENZAPINE 50mg/mL oral suspension 100mL |
|  | d4lC. | CLOZAPINE 50mg/mL oral suspension |
|  | d4lD. | DENZAPINE 50mg tablets |
|  | d4lE. | CLOZAPINE 50mg tablets |
|  | d4lF. | DENZAPINE 200mg tablets |
|  | d4lG. | CLOZAPINE 200mg tablets |
|  | d4m.. | REMOXIPRIDE |
|  | d4m1. | REMOXIPRIDE 150mg m/r capsules |
|  | d4m2. | REMOXIPRIDE 300mg m/r capsules |
|  | d4m3. | *ROXIAM 150mg m/r capsules |
|  | d4m4. | *ROXIAM 300mg m/r capsules |
|  | d4p.. | RISPERIDONE |
|  | d4p1. | RISPERIDONE 1mg tablets |
|  | d4p2. | RISPERIDONE 2mg tablets |
|  | d4p3. | RISPERIDONE 3mg tablets |
|  | d4p4. | RISPERIDONE 4mg tablets |
|  | d4p5. | RISPERDAL 1mg tablets |
|  | d4p6. | RISPERDAL 2mg tablets |
|  | d4p7. | RISPERDAL 3mg tablets |
|  | d4p8. | RISPERDAL 4mg tablets |
|  | d4p9. | RISPERIDONE 1mg/mL liquid |
|  | d4pA. | RISPERDAL 1mg/mL liquid |
|  | d4pB. | RISPERIDONE 6mg tablets |
|  | d4pC. | RISPERDAL 6mg tablets |
|  | d4pD. | RISPERDAL 0.5mg tablets |
|  | d4pE. | RISPERDAL CONSTA 25mg powder+solvent for suspension for injection |
|  | d4pF. | RISPERDAL CONSTA 37.5mg powder+solvent for suspension for injection |
|  | d4pG. | RISPERDAL CONSTA 50mg powder+solvent for suspension for injection |
|  | d4pH. | RISPERIDONE 1mg oro-dispersible tablets |
|  | d4pJ. | RISPERIDONE 2mg oro-dispersible tablets |
|  | d4pK. | RISPERDAL QUICKLET 1mg oro-dispersible tablets |
|  | d4pL. | RISPERDAL QUICKLET 2mg oro-dispersible tablets |
|  | d4pM. | RISPERIDONE 0.5mg oro-dispersible tablets |
|  | d4pN. | RISPERDAL QUICKLET 0.5mg oro-dispersible tablets |
|  | d4pO. | RISPERDAL QUICKLET 3mg oro-dispersible tablets |
|  | d4pP. | RISPERDAL QUICKLET 4mg oro-dispersible tablets |
|  | d4pQ. | RISPERIDONE 3mg oro-dispersible tablets |
|  | d4pR. | RISPERIDONE 4mg oro-dispersible tablets |
|  | d4pw. | RISPERIDONE 50mg powder+solvent for suspension for injection |
|  | d4px. | RISPERIDONE 37.5mg powder+solvent for suspension for injection |
|  | d4py. | RISPERIDONE 25mg powder+solvent for suspension for injection |
|  | d4pz. | RISPERIDONE 0.5mg tablets |
|  | d4q.. | SERTINDOLE |
|  | d4q1. | SERTINDOLE 4mg tablets |
|  | d4q2. | SERTINDOLE 12mg tablets |
|  | d4q3. | SERTINDOLE 16mg tablets |
|  | d4q4. | SERTINDOLE 20mg tablets |
|  | d4q5. | SERDOLECT 4mg tablets |
|  | d4q6. | SERDOLECT 12mg tablets |
|  | d4q7. | SERDOLECT 16mg tablets |
|  | d4q8. | SERDOLECT 20mg tablets |
|  | d4r.. | OLANZAPINE |
|  | d4r1. | OLANZAPINE 5mg tablets |
|  | d4r2. | OLANZAPINE 7.5mg tablets |
|  | d4r3. | OLANZAPINE 10mg tablets |
|  | d4r4. | ZYPREXA 5mg tablets |
|  | d4r5. | ZYPREXA 7.5mg tablets |
|  | d4r6. | ZYPREXA 10mg tablets |
|  | d4r7. | OLANZAPINE 2.5mg tablets |
|  | d4r8. | ZYPREXA 2.5mg tablets |
|  | d4r9. | ZYPREXA VELOTAB 5mg dispersible tablets |
|  | d4rA. | ZYPREXA VELOTAB 10mg dispersible tablets |
|  | d4rB. | ZYPREXA 15mg tablets |
|  | d4rC. | ZYPREXA VELOTAB 15mg dispersible tablets |
|  | d4rD. | ZYPREXA 10mg injection (pdr for recon) |
|  | d4rE. | ZYPREXA VELOTAB 20mg dispersible tablets |
|  | d4rF. | ZYPREXA 20mg tablets |
|  | d4rG. | ZALASTA 2.5mg tablets |
|  | d4rH. | ZALASTA 5mg tablets |
|  | d4rI. | ZALASTA 7.5mg tablets |
|  | d4rJ. | ZALASTA 15mg tablets |
|  | d4rK. | ZALASTA 20mg tablets |
|  | d4rL. | ZALASTA 5mg dispersible tablets |
|  | d4rM. | ZALASTA 10mg dispersible tablets |
|  | d4rN. | ZALASTA 15mg dispersible tablets |
|  | d4rO. | ZALASTA 20mg dispersible tablets |
|  | d4rP. | ZALASTA 10mg tablets |
|  | d4rt. | OLANZAPINE 20mg tablets |
|  | d4ru. | OLANZAPINE 20mg dispersible tablets |
|  | d4rv. | OLANZAPINE 10mg injection (pdr for recon) |
|  | d4rw. | OLANZAPINE 15mg dispersible tablets |
|  | d4rx. | OLANZAPINE 15mg tablets |
|  | d4ry. | OLANZAPINE 5mg dispersible tablets |
|  | d4rz. | OLANZAPINE 10mg dispersible tablets |
|  | d4s.. | QUETIAPINE |
|  | d4s1. | QUETIAPINE 25mg tablets |
|  | d4s2. | QUETIAPINE 100mg tablets |
|  | d4s3. | QUETIAPINE 200mg tablets |
|  | d4s4. | QUETIAPINE 25mg+100mg tablets starter pack |
|  | d4s5. | SEROQUEL 25mg tablets |
|  | d4s6. | SEROQUEL 100mg tablets |
|  | d4s7. | SEROQUEL 200mg tablets |
|  | d4s8. | SEROQUEL 25mg+100mg tablets starter pack |
|  | d4s9. | SEROQUEL 150mg tablets |
|  | d4sA. | SEROQUEL 25mg+100mg+150mg tablets starter pack |
|  | d4sB. | SEROQUEL 300mg tablets |
|  | d4sC. | SEROQUEL XL 50mg m/r tablets |
|  | d4sD. | SEROQUEL XL 200mg m/r tablets |
|  | d4sE. | SEROQUEL XL 300mg m/r tablets |
|  | d4sF. | SEROQUEL XL 400mg m/r tablets |
|  | d4sG. | SEROQUEL XL 150mg m/r tablets |
|  | d4ss. | QUETIAPINE 150mg m/r tablets |
|  | d4st. | QUETIAPINE 400mg m/r tablets |
|  | d4su. | QUETIAPINE 300mg m/r tablets |
|  | d4sv. | QUETIAPINE 200mg m/r tablets |
|  | d4sw. | QUETIAPINE 50mg m/r tablets |
|  | d4sx. | QUETIAPINE 300mg tablets |
|  | d4sy. | QUETIAPINE 25mg+100mg+150mg tablets starter pack |
|  | d4sz. | QUETIAPINE 150mg tablets |
|  | d4t.. | AMISULPRIDE |
|  | d4t1. | AMISULPRIDE 50mg tablets |
|  | d4t2. | AMISULPRIDE 200mg tablets |
|  | d4t3. | SOLIAN 50mg tablets |
|  | d4t4. | SOLIAN 200mg tablets |
|  | d4t5. | SOLIAN 400mg tablets |
|  | d4t6. | SOLIAN 100mg/mL sugar free oral solution |
|  | d4t7. | SOLIAN 100mg tablets |
|  | d4tx. | AMISULPRIDE 100mg tablets |
|  | d4ty. | AMISULPRIDE 100mg/mL sugar free oral solution |
|  | d4tz. | AMISULPRIDE 400mg tablets |
|  | d4u.. | ZOTEPINE |
|  | d4u1. | *ZOTEPINE 25mg tablets |
|  | d4u2. | *ZOTEPINE 50mg tablets |
|  | d4u3. | *ZOTEPINE 100mg tablets |
|  | d4u4. | *ZOLEPTIL 25mg tablets |
|  | d4u5. | *ZOLEPTIL 50mg tablets |
|  | d4u6. | *ZOLEPTIL 100mg tablets |
|  | d4v.. | ARIPIPRAZOLE |
|  | d4v1. | ABILIFY 10mg tablets |
|  | d4v2. | ABILIFY 15mg tablets |
|  | d4v3. | ABILIFY 30mg tablets |
|  | d4v4. | ABILIFY 5mg tablets |
|  | d4v5. | ABILIFY 10mg oro-dispersible tablets |
|  | d4v6. | ABILIFY 15mg oro-dispersible tablets |
|  | d4v7. | ABILIFY 1mg/mL oral solution |
|  | d4v8. | ABILIFY 9.75mg/1.3mL solution for injection |
|  | d4vs. | ARIPIPRAZOLE 9.75mg/1.3mL solution for injection |
|  | d4vt. | ARIPIPRAZOLE 1mg/mL oral solution |
|  | d4vu. | ARIPIPRAZOLE 10mg oro-dispersible tablets |
|  | d4vv. | ARIPIPRAZOLE 15mg oro-dispersible tablets |
|  | d4vw. | ARIPIPRAZOLE 5mg tablets |
|  | d4vx. | ARIPIPRAZOLE 30mg tablets |
|  | d4vy. | ARIPIPRAZOLE 15mg tablets |
|  | d4vz. | ARIPIPRAZOLE 10mg tablets |
|  | d4w.. | PALIPERIDONE |
|  | d4w1. | INVEGA 3mg m/r tablets |
|  | d4w2. | INVEGA 6mg m/r tablets |
|  | d4w3. | INVEGA 9mg m/r tablets |
|  | d4w4. | *INVEGA 12mg m/r tablets |
|  | d4w5. | XEPLION 50mg suspension for injection prefilled syringe |
|  | d4w6. | XEPLION 75mg suspension for injection prefilled syringe |
|  | d4w7. | XEPLION 100mg suspension for injection prefilled syringe |
|  | d4w8. | XEPLION 150mg suspension for injection prefilled syringe |
|  | d4ws. | PALIPERIDONE 150mg suspension for injection pfs |
|  | d4wt. | PALIPERIDONE 100mg suspension for injection pfs |
|  | d4wu. | PALIPERIDONE 75mg suspension for injection prefilled syringe |
|  | d4wv. | PALIPERIDONE 50mg suspension for injection prefilled syringe |
|  | d4ww. | *PALIPERIDONE 12mg m/r tablets |
|  | d4wx. | PALIPERIDONE 9mg m/r tablets |
|  | d4wy. | PALIPERIDONE 6mg m/r tablets |
|  | d4wz. | PALIPERIDONE 3mg m/r tablets |
|  | d4x.. | ASENAPINE |
|  | d4x1. | SYCREST 5mg sublingual tablets |
|  | d4x2. | ASENAPINE 5mg sublingual tablets |
|  | d4x3. | SYCREST 10mg sublingual tablets |
|  | d4x4. | ASENAPINE 10mg sublingual tablets |
|  | d58.. | OLANZAPINE PAMOATE |
|  | d581. | ZYPADHERA 210mg powder+solvent for suspension for injection |
|  | d582. | ZYPADHERA 300mg powder+solvent for suspension for injection |
|  | d583. | ZYPADHERA 405mg powder+solvent for suspension for injection |
|  | d58x. | OLANZAPINE 405mg powder+solvent for suspension for injection |
|  | d58y. | OLANZAPINE 300mg powder+solvent for suspension for injection |
|  | d58z. | OLANZAPINE 210mg powder+solvent for suspension for injection |
|  | d4lE. | CLOZAPINE 50mg tablets |
|  | d4lF. | DENZAPINE 200mg tablets |
|  | d4lG. | CLOZAPINE 200mg tablets |
|  | d4m.. | REMOXIPRIDE |
|  | d4m1. | REMOXIPRIDE 150mg m/r capsules |
|  | d4m2. | REMOXIPRIDE 300mg m/r capsules |
|  | d4m3. | *ROXIAM 150mg m/r capsules |
|  | d4m4. | *ROXIAM 300mg m/r capsules |
|  | d4p.. | RISPERIDONE |
|  | d4p1. | RISPERIDONE 1mg tablets |
|  | d4p2. | RISPERIDONE 2mg tablets |
|  | d4p3. | RISPERIDONE 3mg tablets |
|  | d4p4. | RISPERIDONE 4mg tablets |
|  | d4p5. | RISPERDAL 1mg tablets |
|  | d4p6. | RISPERDAL 2mg tablets |
|  | d4p7. | RISPERDAL 3mg tablets |
|  | d4p8. | RISPERDAL 4mg tablets |
|  | d4p9. | RISPERIDONE 1mg/mL liquid |
|  | d4pA. | RISPERDAL 1mg/mL liquid |
|  | d4pB. | RISPERIDONE 6mg tablets |
|  | d4pC. | RISPERDAL 6mg tablets |
|  | d4pD. | RISPERDAL 0.5mg tablets |
|  | d4pE. | RISPERDAL CONSTA 25mg powder+solvent for suspension for injection |
|  | d4pF. | RISPERDAL CONSTA 37.5mg powder+solvent for suspension for injection |
|  | d4pG. | RISPERDAL CONSTA 50mg powder+solvent for suspension for injection |
|  | d4pH. | RISPERIDONE 1mg oro-dispersible tablets |
|  | d4pJ. | RISPERIDONE 2mg oro-dispersible tablets |
|  | d4pK. | RISPERDAL QUICKLET 1mg oro-dispersible tablets |
|  | d4pL. | RISPERDAL QUICKLET 2mg oro-dispersible tablets |
|  | d4pM. | RISPERIDONE 0.5mg oro-dispersible tablets |
|  | d4pN. | RISPERDAL QUICKLET 0.5mg oro-dispersible tablets |
|  | d4pO. | RISPERDAL QUICKLET 3mg oro-dispersible tablets |
|  | d4pP. | RISPERDAL QUICKLET 4mg oro-dispersible tablets |
|  | d4pQ. | RISPERIDONE 3mg oro-dispersible tablets |
|  | d4pR. | RISPERIDONE 4mg oro-dispersible tablets |
|  | d4pw. | RISPERIDONE 50mg powder+solvent for suspension for injection |
|  | d4px. | RISPERIDONE 37.5mg powder+solvent for suspension for injection |
|  | d4py. | RISPERIDONE 25mg powder+solvent for suspension for injection |
|  | d4pz. | RISPERIDONE 0.5mg tablets |
|  | d4q.. | SERTINDOLE |
|  | d4q1. | SERTINDOLE 4mg tablets |
|  | d4q2. | SERTINDOLE 12mg tablets |
|  | d4q3. | SERTINDOLE 16mg tablets |
|  | d4q4. | SERTINDOLE 20mg tablets |
|  | d4q5. | SERDOLECT 4mg tablets |
|  | d4q6. | SERDOLECT 12mg tablets |
|  | d4q7. | SERDOLECT 16mg tablets |
|  | d4q8. | SERDOLECT 20mg tablets |
|  | d4r.. | OLANZAPINE |
|  | d4r1. | OLANZAPINE 5mg tablets |
|  | d4r2. | OLANZAPINE 7.5mg tablets |
|  | d4r3. | OLANZAPINE 10mg tablets |
|  | d4r4. | ZYPREXA 5mg tablets |
|  | d4r5. | ZYPREXA 7.5mg tablets |
|  | d4r6. | ZYPREXA 10mg tablets |
|  | d4r7. | OLANZAPINE 2.5mg tablets |
|  | d4r8. | ZYPREXA 2.5mg tablets |
|  | d4r9. | ZYPREXA VELOTAB 5mg dispersible tablets |
|  | d4rA. | ZYPREXA VELOTAB 10mg dispersible tablets |
|  | d4rB. | ZYPREXA 15mg tablets |
|  | d4rC. | ZYPREXA VELOTAB 15mg dispersible tablets |
|  | d4rD. | ZYPREXA 10mg injection (pdr for recon) |
|  | d4rE. | ZYPREXA VELOTAB 20mg dispersible tablets |
|  | d4rF. | ZYPREXA 20mg tablets |
|  | d4rG. | ZALASTA 2.5mg tablets |
|  | d4rH. | ZALASTA 5mg tablets |
|  | d4rI. | ZALASTA 7.5mg tablets |
|  | d4rJ. | ZALASTA 15mg tablets |
|  | d4rK. | ZALASTA 20mg tablets |
|  | d4rL. | ZALASTA 5mg dispersible tablets |
|  | d4rM. | ZALASTA 10mg dispersible tablets |
|  | d4rN. | ZALASTA 15mg dispersible tablets |
|  | d4rO. | ZALASTA 20mg dispersible tablets |
|  | d4rP. | ZALASTA 10mg tablets |
|  | d4rt. | OLANZAPINE 20mg tablets |
|  | d4ru. | OLANZAPINE 20mg dispersible tablets |
|  | d4rv. | OLANZAPINE 10mg injection (pdr for recon) |
|  | d4rw. | OLANZAPINE 15mg dispersible tablets |
|  | d4rx. | OLANZAPINE 15mg tablets |
|  | d4ry. | OLANZAPINE 5mg dispersible tablets |
|  | d4rz. | OLANZAPINE 10mg dispersible tablets |
|  | d4s.. | QUETIAPINE |
|  | d4s1. | QUETIAPINE 25mg tablets |
|  | d4s2. | QUETIAPINE 100mg tablets |
|  | d4s3. | QUETIAPINE 200mg tablets |
|  | d4s4. | QUETIAPINE 25mg+100mg tablets starter pack |
|  | d4s5. | SEROQUEL 25mg tablets |
|  | d4s6. | SEROQUEL 100mg tablets |
|  | d4s7. | SEROQUEL 200mg tablets |
|  | d4s8. | SEROQUEL 25mg+100mg tablets starter pack |
|  | d4s9. | SEROQUEL 150mg tablets |
|  | d4sA. | SEROQUEL 25mg+100mg+150mg tablets starter pack |
|  | d4sB. | SEROQUEL 300mg tablets |
|  | d4sC. | SEROQUEL XL 50mg m/r tablets |
|  | d4sD. | SEROQUEL XL 200mg m/r tablets |
|  | d4sE. | SEROQUEL XL 300mg m/r tablets |
|  | d4sF. | SEROQUEL XL 400mg m/r tablets |
|  | d4sG. | SEROQUEL XL 150mg m/r tablets |
|  | d4ss. | QUETIAPINE 150mg m/r tablets |
|  | d4st. | QUETIAPINE 400mg m/r tablets |
|  | d4su. | QUETIAPINE 300mg m/r tablets |
|  | d4sv. | QUETIAPINE 200mg m/r tablets |
|  | d4sw. | QUETIAPINE 50mg m/r tablets |
|  | d4sx. | QUETIAPINE 300mg tablets |
|  | d4sy. | QUETIAPINE 25mg+100mg+150mg tablets starter pack |
|  | d4sz. | QUETIAPINE 150mg tablets |
|  | d4t.. | AMISULPRIDE |
|  | d4t1. | AMISULPRIDE 50mg tablets |
|  | d4t2. | AMISULPRIDE 200mg tablets |
|  | d4t3. | SOLIAN 50mg tablets |
|  | d4t4. | SOLIAN 200mg tablets |
|  | d4t5. | SOLIAN 400mg tablets |
|  | d4t6. | SOLIAN 100mg/mL sugar free oral solution |
|  | d4t7. | SOLIAN 100mg tablets |
|  | d4tx. | AMISULPRIDE 100mg tablets |
|  | d4ty. | AMISULPRIDE 100mg/mL sugar free oral solution |
|  | d4tz. | AMISULPRIDE 400mg tablets |
|  | d4u.. | ZOTEPINE |
|  | d4u1. | *ZOTEPINE 25mg tablets |
|  | d4u2. | *ZOTEPINE 50mg tablets |
|  | d4u3. | *ZOTEPINE 100mg tablets |
|  | d4u4. | *ZOLEPTIL 25mg tablets |
|  | d4u5. | *ZOLEPTIL 50mg tablets |
|  | d4u6. | *ZOLEPTIL 100mg tablets |
|  | d4v.. | ARIPIPRAZOLE |
|  | d4v1. | ABILIFY 10mg tablets |
|  | d4v2. | ABILIFY 15mg tablets |
|  | d4v3. | ABILIFY 30mg tablets |
|  | d4v4. | ABILIFY 5mg tablets |
|  | d4v5. | ABILIFY 10mg oro-dispersible tablets |
|  | d4v6. | ABILIFY 15mg oro-dispersible tablets |
|  | d4v7. | ABILIFY 1mg/mL oral solution |
|  | d4v8. | ABILIFY 9.75mg/1.3mL solution for injection |
|  | d4vs. | ARIPIPRAZOLE 9.75mg/1.3mL solution for injection |
|  | d4vt. | ARIPIPRAZOLE 1mg/mL oral solution |
|  | d4vu. | ARIPIPRAZOLE 10mg oro-dispersible tablets |
|  | d4vv. | ARIPIPRAZOLE 15mg oro-dispersible tablets |
|  | d4vw. | ARIPIPRAZOLE 5mg tablets |
|  | d4vx. | ARIPIPRAZOLE 30mg tablets |
|  | d4vy. | ARIPIPRAZOLE 15mg tablets |
|  | d4vz. | ARIPIPRAZOLE 10mg tablets |
|  | d4w.. | PALIPERIDONE |
|  | d4w1. | INVEGA 3mg m/r tablets |
|  | d4w2. | INVEGA 6mg m/r tablets |
|  | d4w3. | INVEGA 9mg m/r tablets |
|  | d4w4. | *INVEGA 12mg m/r tablets |
|  | d4w5. | XEPLION 50mg suspension for injection prefilled syringe |
|  | d4w6. | XEPLION 75mg suspension for injection prefilled syringe |
|  | d4w7. | XEPLION 100mg suspension for injection prefilled syringe |
|  | d4w8. | XEPLION 150mg suspension for injection prefilled syringe |
|  | d4ws. | PALIPERIDONE 150mg suspension for injection pfs |
|  | d4wt. | PALIPERIDONE 100mg suspension for injection pfs |
|  | d4wu. | PALIPERIDONE 75mg suspension for injection prefilled syringe |
|  | d4wv. | PALIPERIDONE 50mg suspension for injection prefilled syringe |
|  | d4ww. | *PALIPERIDONE 12mg m/r tablets |
|  | d4wx. | PALIPERIDONE 9mg m/r tablets |
|  | d4wy. | PALIPERIDONE 6mg m/r tablets |
|  | d4wz. | PALIPERIDONE 3mg m/r tablets |
|  | d4x.. | ASENAPINE |
|  | d4x1. | SYCREST 5mg sublingual tablets |
|  | d4x2. | ASENAPINE 5mg sublingual tablets |
|  | d4x3. | SYCREST 10mg sublingual tablets |
|  | d4x4. | ASENAPINE 10mg sublingual tablets |
|  | d58.. | OLANZAPINE PAMOATE |
|  | d581. | ZYPADHERA 210mg powder+solvent for suspension for injection |
|  | d582. | ZYPADHERA 300mg powder+solvent for suspension for injection |
|  | d583. | ZYPADHERA 405mg powder+solvent for suspension for injection |
|  | d58x. | OLANZAPINE 405mg powder+solvent for suspension for injection |
|  | d58y. | OLANZAPINE 300mg powder+solvent for suspension for injection |
|  | d58z. | OLANZAPINE 210mg powder+solvent for suspension for injection |
