# Supplementary material for: Longitudinal analysis of the relationship between motor and psychiatric symptoms in idiopathic dystonia
Source: Eur J Neurol. 2022 Sep 11;29(12):3513–27. doi: 10.1111/ene.15530 (PMC9826317; doi:10.1111/ene.15530)
Supplement: Supplementary file 7 — TABLE S5 [file ENE-29-3513-s009.docx]

**Supplementary Table 5: Benzodiazepine codes used to remove individuals with no recorded psychiatric diagnoses**

| Hypnotics |  |  |
| --- | --- | --- |
|  | d14.. | Flumtrazepam - discontinued |
|  | d15.. | Flurazepam |
|  | d16.. | Loprazolam |
|  | d17.. | Lormetazepam |
|  | d18.. | Nitrazepam |
|  | d1a.. | Temazepam (hynotic) |
|  | d1b.. | Triazolam - discontinued |
| Anxiolytics |  |  |
|  | d21.. | Diazepam |
|  | d22.. | Alprazolam |
|  | d23.. | Bromazepam |
|  | d26.. | Clobazam |
|  | d29.. | Ketazolam - discontinued |
|  | d2a.. | Lorazepam (anxiolytic) |
|  | d2b.. | Medazepam - discontinued |
|  | d2d.. | Oxazepam |
