# Supplementary material for: Longitudinal analysis of the relationship between motor and psychiatric symptoms in idiopathic dystonia
Source: Eur J Neurol. 2022 Sep 11;29(12):3513–27. doi: 10.1111/ene.15530 (PMC9826317; doi:10.1111/ene.15530)
Supplement: Supplementary file 8 — TABLE S6 [file ENE-29-3513-s004.docx]

**Supplementary Table 6. Number of different psychiatric diagnoses/prescribed medications**

|  | **Overall (%)** | **Cervical dystonia (%)** | **Blepharospasm (%)** | **Tremor (%)** | **Other (%)** | **Controls (%)** |
| --- | --- | --- | --- | --- | --- | --- |
| ***Over 20s*** |  |  |  |  |  |  |
| ***Overall*** |  |  |  |  |  |  |
| Depression | **13,844 (26.3)** | **8,828 (24.3)** | <> | **4,488 (31.4)** | **174 (30.1)** | 39,683 (18.3) |
| Anxiety | **11,526 (21.9)** | **7,029 (19.3)** | **305 (24.2)** | **4,048 (28.3)** | **129 (22.3)** | 30,871 (14.3) |
| Substance use disorder | **3,109 (5.9)** | **1,742 (4.8)** | <> | **1,241 (8.7)** | <> | 9,520 (4.4) |
| Eating disorder | **693 (1.3)** | **381 (1)** | <> | <> | <> | 1,659 (0.8) |
| Severe mental illness | **415 (0.8)** | **169 (0.5)** | <> | **214 (1.5)** | <> | 2,301 (1.1) |
| Conduct disorder | 65 (0.1) | 40 (0.1) | <> | <> | <> | 245 (0.1) |
| ADHD | 42 (0.08) | 26 (0.07) | <> | <> | <> | 147 (0.07) |
| ASD | 40 (0.08) | 14 (0.04) | <> | <> | <> | 160 (0.07) |
| ***Pre-dystonia*** |  |  |  |  |  |  |
| Depression | **8,539 (16.2)** | **5,113 (14.1)** | <> | **3,092 (21.6)** | <> | 22,987 (10.6) |
| Anxiety | **6,570 (12.5)** | **3,690 (10.2)** | <> | **2,605 (18.2)** | <> | 16,445 (7.6) |
| Substance use disorder | **1,875 (3.6)** | 995 (2.7) | <> | **796 (5.6)** | <> | 5,671 (2.6) |
| Eating disorder | **387 (0.7)** | **219 (0.6)** | <> | <> | <> | 948 (0.4) |
| Severe mental illness | **157 (0.3)** | **49 (0.1)** | <> | <> | <> | 1,254 (0.6) |
| Conduct disorder | 44 (0.08) | 31 (0.09) | <> | <> | <> | 178 (0.08) |
| ADHD | 22 (0.04) | 16 (0.04) | <> | <> | <> | 76 (0.04) |
| ASD | 14 (0.03) | 6 (0.02) | <> | <> | <> | 87 (0.04) |
| ***Post-dystonia*** |  |  |  |  |  |  |
| Depression | **5,305 (10.1)** | **3,715 (10.2)** | <> | **1,396 (9.8)** | <> | 16,696 (7.7) |
| Anxiety | **4,956 (9.4)** | **3,339 (9.2)** | **<>** | **1,443 (10.1)** | <> | 14,426 (6.7) |
| Substance use disorder | **1,234 (2.3)** | **747 (2.1)** | <> | **445 (3.1)** | <> | 3,849 (1.8) |
| Eating disorder | **306 (0.6)** | **162 (0.4)** | <> | <> | <> | 711 (0.3) |
| Severe mental illness | 258 (0.5) | **<>** | <> | <> | <> | 1,047 (0.5) |
| ASD | 26 (0.05) | 8 (0.02) | <> | <> | <> | 73 (0.03) |
| Conduct disorder | 21 (0.04) | 9 (0.02) | - | **12 (0.08)** | - | 67 (0.03) |
| ADHD | 20 (0.04) | 10 (0.03) | <> | <> | <> | 71 (0.03) |
| ***Under 20s*** |  |  |  |  |  |  |
| ***Overall*** |  |  |  |  |  |  |
| Depression | **2,186 (4.2)** | **1,654 (4,6)** | <> | 497 (3.5) | 20 (3.5) | 6,884 (3.2) |
| Anxiety | **1,942 (3.7)** | **1,396 (3.8)** | **14 (1.1)** | **506 (3.5)** | 21 (3.6) | 5,595 (2.6) |
| Substance use disorder | **655 (1.2)** | **505 (1.4)** | <> | 133 (0.9) | <> | 2,256 (1) |
| Eating disorder | **240 (0.5)** | **175 (0.5)** | <> | <> | <> | 686 (0.3) |
| ADHD | **237 (0.5)** | **170 (0.5)** | <> | <> | <> | 677 (0.3) |
| Conduct disorder | **230 (0.4)** | **173 (0.5)** | <> | <> | <> | 585 (0.3) |
| ASD | **201 (0.4)** | **139 (0.4)** | <> | <> | <> | 603 (0.3) |
| Severe mental illness | 68 (0.1) | 41 (0.1) | <> | 20 (0.1) | <> | 264 (0.1) |
| ***Pre-dystonia*** |  |  |  |  |  |  |
| Anxiety | **291 (0.6)** | **155 (0.4)** | <> | **123 (0.9)** | <> | 635 (0.3) |
| Depression | **263 (0.5)** | 142 (0.4) | <> | **109 (0.8)** | <> | 708 (0.3) |
| Conduct disorder | **124 (0.2)** | **89 (0.2)** | <> | <> | <> | 340 (0.2) |
| Substance use disorder | **124 (0.2)** | 75 (0.2) | <> | **43 (0.3)** | <> | 371 (0.2) |
| ADHD | **115 (0.2)** | **83 (0.2)** | <> | 26 (0.2) | <> | 309 (0.1) |
| Eating disorder | **100 (0.2)** | **68 (0.2)** | <> | <> | <> | 237 (0.1) |
| ASD | 80 (0.2) | 57 (0.2) | <> | <> | <> | 245 (0.1) |
| Severe mental illness | 6 (0.01) | <> | - | <> | <> | 23 (0.01) |
| ***Post-dystonia*** |  |  |  |  |  |  |
| Depression | **1,923 (3.7)** | **1,512 (4.2)** | <> | 388 (2.7) | <> | 6,176 (2.9) |
| Anxiety | **1,651 (3.1)** | **1,241 (3.4)** | <> | 383 (2.7) | <> | 4,960 (2.3) |
| Substance use disorder | **531 (1)** | **430 (1.2)** | <> | **90 (0.6)** | <> | 1,885 (0.9) |
| Eating disorder | 140 (0.3) | **107 (0.3)** | <> | 33 (0.2) | <> | 449 (0.2) |
| ADHD | 122 (0.2) | **87 (0.2)** | - | 35 (0.2) | - | 368 (0.2) |
| ASD | **121 (0.2)** | 82 (0.2) | <> | <> | <> | 358 (0.2) |
| Conduct disorder | **106 (0.2)** | **84 (0.2)** | <> | <> | <> | 245 (0.1) |
| Severe mental illness | 62 (0.1) | <> | <> | <> | <> | 241 (0.1) |
| **Prescriptions** |  |  |  |  |  |  |
| ***Over 20s*** |  |  |  |  |  |  |
| Antidepressant | **17,695 (33.6)** | **11,126 (30.6)** | **452 (33.7)** | **5,847 (40.9)** | **244 (42.2)** | 57,390 (26.5) |
| Anxiolytic | **10,477 (19.9)** | **6,777 (18.6)** | <> | **3,286 (23)** | **<>** | 28,291 (13.1) |
| Hypnotic | **7,571 (14.4)** | **4,577 (12.6)** | <> | **2,682 (18.7)** | <> | 24,371 (11.3) |
| Antipsychotic | **1,020 (1.9)** | **481 (1.3)** | <> | **478 (3.3)** | <> | 5,537 (2.6) |
| ***Pre-dystonia*** |  |  |  |  |  |  |
| Antidepressants | **10,989 (20.9)** | **6,382 (17.6)** | <> | **4,134 (28.9)** | <> | 31,601 (14.6) |
| Anxiolytics | **6,721 (12.9)** | **4,328 (11.9)** | <> | **<>** | <> | 13,437 (6.2) |
| Hypnotics | **4,127 (7.8)** | 2,218 (6.1) | <> | <> | <> | 12,679 (5.9) |
| Antipsychotics | **<>** | <> | <> | **<>** | <> | 2,459 (1.1) |
| ***Post-dystonia*** |  |  |  |  |  |  |
| Antidepressant | **6,706 (12.8)** | **4,744 (13.1)** | <> | 1,713 (11.2) | <> | 25,789 (11.9) |
| Anxiolytic | 3,756 (7.1) | 2,449 (6.7) | <> | **<>** | <> | 14,854 (6.9) |
| Hypnotic | **3,444 (6.5)** | **2,359 (6.5)** | <> | **<>** | <> | 11,692 (5.4) |
| Antipsychotic | **<>** | <> | <> | **<>** | <> | 3,078 (1.4) |
| ***Under 20s*** |  |  |  |  |  |  |
| Antidepressant | **2,354 (4.5)** | **1,775 (4.9)** | **14 (1.1)** | 543 (3.8) | 17 (2.9) | 7,440 (3.4) |
| Anxiolytic | **820 (1.6)** | **624 (1.7)** | <> | 167 (1.2) | **<>** | 2,228 (1) |
| Hypnotic | **662 (1.3)** | **498 (1.4)** | <> | 148 (1) | <> | 1,983 (0.9) |
| Antipsychotic | 144 (0.3) | 94 (0.3) | <> | 42 (0.3) | <> | 549 (0.3) |
| ***Pre-dystonia*** |  |  |  |  |  |  |
| Antidepressants | **232 (0.4)** | **127 (0.3)** | <> | **93 (0.6)** | <> | 534 (0.2) |
| Anxiolytics | **223 (0.4)** | **185 (0.5)** | <> | **<>** | <> | 178 (0.08) |
| Hypnotics | 61 (0.1) | 41 (0.1) | <> | <> | <> | 196 (0.09) |
| Antipsychotics | <> | <> | <> | <> | <> | 62 (0.0.03) |
| ***Post-dystonia*** |  |  |  |  |  |  |
| Antidepressant | **2,122 (4)** | **1,648 (4.5)** | <> | 450 (3.1) | <> | 6,906 (3.2) |
| Hypnotic | **601 (1.1)** | **457 (1.3)** | <> | <> | <> | 1,787 (0.8) |
| Anxiolytic | **597 (1.1)** | **439 (1.2)** | <> | <> | <> | 2,050 (0.9) |
| Antipsychotic | <> | <> | <> | <> | <> | 487 (0.2) |
| ***Number of different types of prescribed anxiolytics*** |  |  |  |  |  |  |
| 1 | **9,963 (88.2)** | 6,730 (90.9) | **205 (84.4)** | **2,852 (82.6)** | 155 (87.1) | 27,773 (91) |
| 2 | **1,198 (10.6)** | 599 (8.1) | <> | <> | <> | 2,456 (8) |
| ≥3 | 136 (1.2) | 72 (1) | <> | <> | <> | 290 (1) |
| ***Number of different types of prescribed antidepressants*** | |  |  |  |  |  |
| 1 | **8,636 (43.1)** | **5,727 (44.4)** | 220 (47.2) | **2,571 (40.2)** | **104 (39.8)** | 34,060 (52.5) |
| 2 | **4,859 (24.2)** | 3,112 (24.1) | 120 (27.8) | 1,548 (24.2) | 71 (27.2) | 15,083 (23.3) |
| ≥3 | **6,546 (32.7)** | **4,058 (31.5)** | 126 (27) | **2,267 (35.5)** | **86 (33)** | 15,662 (24.2) |

**Abbreviations:** ADHD: Attention defective disorder, ASD: Autism spectrum disorder, OCD: Obsessive compulsive disorder, SD: Standard Deviation, SMI: Severe mental illness, SUD: Substance use disorder

**Note:** percentages can be >100% where participants had more than one diagnoses/prescriptions

P-values are all vs controls. Bold p-values represent significant values post Bonferroni correction for multiple comparisons
