# Supplementary material for: Longitudinal analysis of the relationship between motor and psychiatric symptoms in idiopathic dystonia
Source: Eur J Neurol. 2022 Sep 11;29(12):3513–27. doi: 10.1111/ene.15530 (PMC9826317; doi:10.1111/ene.15530)
Supplement: Supplementary file 9 — TABLE S7 [file ENE-29-3513-s010.docx]

**Supplementary Table 7. Number of pre and post mental diagnoses and median time (years) from/to first mental disorder diagnosis/medication from/to index date**

|  | **Overall** | | **Cervical dystonia** | | **Blepharospasm** | | **Tremor** | **Other** |
| --- | --- | --- | --- | --- | --- | --- | --- | --- |
| **Diagnosis** |  | |  | |  | |  |  |
| ***Overall*** |  | |  | |  | |  |  |
| *Pre dystonia* |  | |  | |  | |  |  |
| Median time between mental disorder and dystonia (IQR) | **4.45 (5.8)** | | **4.58 (5.62)** | | 5.05 (6.15) | | **4.13 (6.12)** | 4.62 (5.8) |
| *Post dystonia* |  | |  | |  | |  |  |
| Median time between diagnoses (IQR) | **3.86 (5.44)** | | **4.33 (5.49)** | | **3.72 (4.78)** | | **2.42 (4.72)** | 3.03 (5.59) |
| ***Over 20*** |  | |  | |  | |  |  |
| *Pre dystonia* |  | |  | |  | |  |  |
| Median time between mental disorder and dystonia (IQR) | **4.68 (5.81)** | | **4.8 (5.62)** | | 5.22 (6.09) | | **4.4 (6.15)** | 4.96 (5.31) |
| *Post dystonia* |  | |  | |  | |  |  |
| Median time between diagnoses (IQR) | **3.19 (4.93)** | | **3.6 (4.99)** | | 3.43 (4.56) | | **1.97 (4.45)** | 2.78 (5.03) |
| ***Under 20*** |  | |  | |  | |  |  |
| *Pre dystonia* |  | |  | |  | |  |  |
| Median time between mental disorder and dystonia (IQR) | **2.08 (3.79)** | | **2.38 (3.69)** | | 0.99 (0.23) | | 1.62 (3.7) | **1.37 (5.9)** |
| *Post dystonia* |  | |  | |  | |  |  |
| Median time between diagnoses (SD) (overall) | **5.4 (5.64)** | | 5.79 (5.35) | | 5.23 (4.91) | | **3.6 (5.27)** | 4.92 (4.9) |
| **Medication** |  |  | |  | |  |  |  |
| ***Overall*** |  | |  | |  | |  |  |
| *Pre dystonia* |  | |  | |  | |  |  |
| Median time between mental disorder and dystonia (IQR) | **3.96 (6.02)** | | **3.69 (5.83)** | | 5.37 (5.82) | | **4.4 (6.21)** | **3.35 (5.45)** |
| *Post dystonia* |  | |  | |  | |  |  |
| Median time between diagnoses (IQR) | **3.83 (5.69)** | | 4.31 (5.69) | | **3.3 (5.56)** | | **2.62 (4.83)** | **2.63 (5.67)** |
| ***Over 20*** |  | |  | |  | |  |  |
| *Pre dystonia* |  | |  | |  | |  |  |
| Median time between mental disorder and dystonia (IQR) | **4.12 (5.98)** | | **3.85 (5.82)** | | 5.38 (5.81) | | **4.55 (6.16)** | **3.54 (5.72)** |
| *Post dystonia* |  | |  | |  | |  |  |
| Median time between diagnoses (IQR) | **3.15 (5.07)** | | **3.49 (5.19)** | | **3.01 (5.22)** | | **2.2 (4.32)** | 2.63 (5.12) |
| ***Under 20*** |  | |  | |  | |  |  |
| *Pre dystonia* |  | |  | |  | |  |  |
| Median time between mental disorder and dystonia (IQR) | **0.54 (1.84)** | | **0.29 (1.71)** | | <> | | **0.97 (1.84)** | 0.76 (1.27) |
| *Post dystonia* |  | |  | |  | |  |  |
| Median time between diagnoses (IQR) | **5.96 (5.62)** | | 6.38 (5.32) | | 6.98 (6.5) | | **3.87 (5.36)** | **2.57 (6.7)** |

P-values are all vs controls using Mann-Whitney U test. Bold p-values represent significant values post Bonferroni correction for multiple comparisons
