# Supplementary material for: Longitudinal analysis of the relationship between motor and psychiatric symptoms in idiopathic dystonia
Source: Eur J Neurol. 2022 Sep 11;29(12):3513–27. doi: 10.1111/ene.15530 (PMC9826317; doi:10.1111/ene.15530)
Supplement: Supplementary file 10 — TABLE S8 [file ENE-29-3513-s007.docx]

**Supplementary Table 8A. Association of psychiatric diagnosis in dystonia in relation to controls**

|  | **Diagnosis overall** | | **ADHD** | | **Anxiety disorder** | | **ASD** | | **Conduct disorder** | | **Depression** | | **Eating disorder** | | **SMI** | | **SUD** | |
| --- | --- | --- | --- | --- | --- | --- | --- | --- | --- | --- | --- | --- | --- | --- | --- | --- | --- | --- |
|  | OR:95 % CI | OR:95 % CI^α^ | OR:95 % CI | OR:95 % CI ^α^ | OR:95 % CI | OR:95 % CI^α^ | OR:95 % CI | OR:95 % CI^α^ | OR:95 % CI | OR:95 % CI^α^ | OR:95 % CI | OR:95 % CI^α^ | OR:95 % CI | OR:95 % CI^α^ | OR:95 % CI | OR:95 % CI^α^ | OR:95 % CI | OR:95 % CI^α^ |
| Dystonia | **1.68:1.64-1.71** | **1.62:1.59-1.66** | **1.4:1.22-1.6** | **1.41:1.22-1.61** | **1.7:1.66-1.74** | **1.64:1.61-1.68** | **1.3:1.12-1.5** | **1.33:.1.15-1.54** | **1.47:1.28-1.67** | **1.42:1.25-1.63** | **1.6:1.57-1.64** | **1.55:1.51-1.58** | **1.65:1.53-1.78** | **1.62:1.5-1.75** | **0.77:0.7-0.85** | **0.76:0.69-0.84** | **1.34:1.29-1.39** | **1.3:1.25-1.35** |
| *Sex* |  |  |  |  |  |  |  |  |  |  |  |  |  |  |  |  |  |  |
| Female | **1.75:1.7-1.79** | **1.69:1.64-1.73** | **1.48:1.12-1.95** | **1.49:1.12-1.96** | **1.73:1.68-1.79** | **1.67:1.63-1.72** | 1.25:0.93-1.66 | 1.28:0.95-1.7 | **1.47:1.92-1.8** | **1.42:1.15-1.74** | **1.63:1.59-1.68** | **1.58:1.54-1.62** | **1.66:1.52-1.81** | **1.63:1.49-1.77** | **0.77:0.68-0.87** | **0.75:0.66-0.85** | **1.32:1.25-1.39** | **1.28:1.21-1.35** |
| Male | **1.61:1.56-1.66** | **1.56:1.51-1.61** | **1.37:1.17-1.6** | **1.37:1.17-1.6** | **1.69:1.62-1.77** | **1.64:1.58-1.71** | **1.32:1.11-1.56** | **1.34:1.13-1.58** | **1.46:1.23-1.74** | **1.43:1.2-1.7** | **1.59:1.53-1.65** | **1.54:1.48-1.6** | **1.64:1.4-1.92** | **1.62:1.38-1.89** | **0.78:0.67-0.92** | **0.77:0.66-0.9** | **1.36:1.29-1.44** | **1.32:1.25-1.39** |
| *Age at index date* | | | | | | |  |  |  |  |  |  |  |  |  |  |  |  |
| < 20 years | **1.43:1.37-1.49** | **1.4:1.34-1.47** | **1.41:1.21-1.64** | **1.4:1.2-1.62** | **1.46:1.38-1.54** | **1.42:1.34-1.51** | **1.34:1.14-1.57** | **1.35:1.15-1.59** | **1.59:1.36-1.85** | **1.55:1.33-1.81** | **1.33:1.26-1.4** | **1.29:1.22-1.36** | **1.41:1.21-1.63** | **1.38:1.19-1.6** | 1.03:0.79-1.34 | 1-0.76:1.3 | **1.17:1.07-1.28** | **1.14:1.04-1.24** |
| ≥ 20 years | **1.78:1.74-1.82** | **1.72:1.68-1.76** | **1.19:0.83-1.66** | **1.21:0.85-1.69** | **1.78:1.73-1.82** | **1.72:1.68-1.77** | 1.04:0.72-1.45 | 1.01:0.98-1.04 | 1.1:0.83-1.44 | 1.06:0.8-1.38 | **1.69:1.65-1.73** | **1.64:1.6-1.68** | **1.75:1.6-1.91** | **1.72:1.57-1.88** | **0.75:0.67-0.83** | **0.74:0.66-0.82** | **1.39:1.33-1.45** | **1.35:1.29-1.41** |
| *WIMD at index date* | | | | | | |  |  |  |  |  |  |  |  |  |  |  |  |
| 1 | **1.7:1.63-1.78** | **1.64:1.57-1.71** | **1.53:1.19-1.94** | **1.53:1.19-1.95** | **1.67:1.59-1.75** | **1.61:1.53-1.69** | 1.42:1.03-1.92 | 1.43:1.04-1.94 | **1.54:1.21-1.94** | **1.49:1.17-1.88** | **1.54:1.47-1.62** | **1.48:1.41-1.55** | **1.49:1.26-1.75** | **1.45:1.23-1.71** | **0.69:0.56-0.84** | **0.67:0.55-0.81** | **1.28:1.19-1.37** | **1.23:1.14-1.32** |
| 2 | **1.66:1.59-1.74** | **1.62:1.55-1.69** | 1.04:0.76-1.4 | 1.05-0.76-1.41 | **1.74:1.66-1.83** | **1.69:1.61-1.78** | 1.07:0.78-1.45 | 1.12:0.81-1.51 | 1.33:0.99-1.76 | 1.31:0.97-1.73 | **1.62:1.54-1.69** | **1.57:1.5-1.64** | **1.81:1.53-2.13** | **1.77:1.5-2.09** | 0.78:0.63-0.95 | 0.77:0.63-0.94 | **1.27:1.17-1.38** | **1.24:1.14-1.34** |
| 3 | **1.69:1.62-1.76** | **1.63:1.56-1.7** | **1.71:1.26-2.29** | **1.74:1.28-2.33** | **1.67:1.59-1.75** | **1.6:1.53-1.69** | 1.35:0.97-1.85 | 1.38:0.99-1.89 | 1.48:1.08-1.99 | 1.44:1.05-1.94 | **1.61:1.53-1.68** | **1.54:1.47-1.62** | **1.66:1.4-1.95** | **1.62:1.37-1.91** | 0.75:0.6-0.94 | 0.75:0.59-0.93 | **1.43:1.32-1.56** | **1.39:1.27-1.51** |
| 4 | **1.65:1.58-1.73** | **1.6:1.53-1.68** | 1.38:0.95-1.95 | 1.38:0.95-1.96 | **1.7:1.61-1.79** | **1.64:1.56-1.74** | 1.54:1.09-2.13 | 1.55:1.1-1.02 | 1.42:0.99-1.99 | 1.37:0.96-1.91 | **1.63:1.55-1.71** | **1.57:1.5-1.66** | **1.79:1.5-2.13** | **1.76:1.47-2.09** | 0.76:0.59-0.97 | 0.75:0.58-0.96 | **1.4:1.27-1.54** | **1.35:1.23-1.49** |
| 5 | **1.72:1.65-1.8** | 1**1.68:1.61-1.76** | 1.39:0.94-1.99 | 1.41:0.96-2.03 | **1.76:1.67-1.86** | **1.72:1.63-1.81** | 1.22:0.84-1.72 | 1.25:0.86-1.78 | 1.61:1.09-2.33 | 1.58:1.06-2.29 | **1.67:1.58-1.75** | **1.63:1.55-1.71** | **1.53:1.27-1.84** | **1.51:1.25-1.82** | 0.97:0.76-1.22 | 0.96:0.75-1.21 | **1.46:1.31-1.62** | **1.43:1.28-1.59** |

Bold values show statistically significant OR with Bonferroni correction (p = 0.005)

^α^ORs and CIs estimated by logistic regression and adjusted for time in study

**Abbreviations:** ADHD: Attention Deficit Hyperactive Disorder, ASD: Autism Spectrum Disorder, CI: 95% Confidence Intervals, OR: odds ratios, SMI: Severe Mental Illness, SUD: Substance Use Disorder

**Supplementary Table 8B. Association of psychiatric diagnosis in cervical dystonia in relation to controls**

|  | **Diagnosis overall** | | **ADHD** | | **Anxiety disorder** | | **ASD** | | **Conduct disorder** | | **Depression** | | **Eating disorder** | | **SMI** | | **SUD** | |
| --- | --- | --- | --- | --- | --- | --- | --- | --- | --- | --- | --- | --- | --- | --- | --- | --- | --- | --- |
|  | OR:95 % CI | OR:95 % CI^α^ | OR:95 % CI | OR:95 % CI ^α^ | OR:95 % CI | OR:95 % CI^α^ | OR:95 % CI | OR:95 % CI^α^ | OR:95 % CI | OR:95 % CI^α^ | OR:95 % CI | OR:95 % CI^α^ | OR:95 % CI | OR:95 % CI^α^ | OR:95 % CI | OR:95 % CI^α^ | OR:95 % CI | OR:95 % CI^α^ |
| Cervical dystonia | **1.49:1.45-1.52** | **1.41:1.38-1.44** | **1.42:1.21-1.66** | **1.43:1.22-1.67** | **1.49:1.45-1.53** | **1.41:1.37-1.45** | **1.2:1.0:1.42** | 1.23:1.03-1.46 | **1.53:1.31-1.78** | **1.47:1.26-1.7** | **1.48:1.44-1.52** | **1.4:1.37-1.44** | **1.42:1.29-1.56** | **1.38:1.25-1.51** | **0.48:0.42-0.56** | **0.47:0.41-0.54** | **1.15:1.09-1.2** | **1.09:1.04-1.14** |
| *Sex* |  |  |  |  |  |  |  |  |  |  |  |  |  |  |  |  |  |  |
| Female | **1.6:1.55-1.65** | **1.51:1.47-1.55** | 1.19:0.83-1.67 | 1.19:0.83-1.67 | **1.55:1.5-1.6** | **1.47:1.42-1.52** | 0.93:0.63-1.34 | 0.95:0.64-1.37 | **1.56:1.23-1.96** | **1.47:1.16-1.85** | **1.57:1.52-1.62** | **1.48:1.44-1.53** | **1.44:1.29-1.6** | **1.4:1.26-1.55** | **0.46:0.38-0.55** | **0.45:0.37-0.54** | **1.19:1.11-1.27** | **1.13:1.06-1.21** |
| Male | **1.33:1.28-1.38** | **1.26:1.21-1.31** | **1.5:1.26-1.78** | **1.5:1.26-1.79** | **1.37:1.3-1.44** | **1.3:1.24-1.37** | 1.3:1.06-1.57 | 1.32:1.08-1.61 | **1.52:1.24-1.85** | **1.46:1.2-1.78** | **1.32:1.26-1.38** | **1.26:1.2-1.32** | **1.34:1.09-1.62** | 1.31:1.07-1.59 | **0.52:0.41-0.65** | **0.51:0.4-0.63** | **1.11:1.04-1.18** | 1.05:0.98-1.13 |
| *Age at index date* | | | | | | |  |  |  |  |  |  |  |  |  |  |  |  |
| < 20 years | **1.31:1.25-1.38** | **1.25:1.19-1.31** | 1.27:1.49 | 1.25:1.05-1.48 | **1.29:1.21-1.37** | **1.23:1.15-1.31** | 1.16:0.96-1.39 | 1.17:0.97-1.4 | **1.49:1.26-1.77** | **1.44:1.21-1.7** | **1.25:1.17-1.32** | **1.18:1.11-1.25** | **1.29:1.08-1.52** | 1.24:1.05-1.46 | 0.78:0.55-1.07 | 0.74:0.52-1.02 | 1.13:1.02-1.35 | 1.07:0.97-1.18 |
| ≥ 20 years | **1.62:1.58-1.66** | **1.53:1.49-1.58** | 1.12:0.72-1.67 | 1.15:0.74-1.72 | **1.61:1.56-1.66** | **1.53:1.48-1.57** | 0.55:0.31-0.92 | 0.55:0.3-0.91 | 1.04:0.73-1.43 | 0.97-0.68 | **1.62:1.58-1.67** | **1.54:1.5-1.58** | **1.46:1.31-1.63** | **1.43:1.27-1.59** | **0.46:0.39-0.54** | **0.45:0.39-0.53** | **1.17:1.11-1.24** | **1.12:1.06-1.18** |
| *WIMD at index date* | | | | | | |  |  |  |  |  |  |  |  |  |  |  |  |
| 1 | **1.5:1.43-1.58** | **1.4:1.33-1.47** | **1.61:1.21-2.11** | **1.61:1.21-2.12** | **1.44:1.36-1.53** | **1.34:1.27-1.43** | 1.52:1.06-2.14 | 1.54:1.07-2.17 | **1.67:1.28-2.17** | **1.58:1.2-2.05** | **1.44:1.37-1.52** | **1.34:1.27-1.42** | 1.19:0.96-1.47 | 1.15:0.93-1.41 | **0.45:0.33-0.59** | **0.43:0.32-0.56** | 1.01:1.01-1.2 | 1.04:0.95-1.13 |
| 2 | **1.48:1.41-1.56** | **1.4:1.33-1.48** | 0.97:0.66-1.38 | 0.97:0.66-1.38 | **1.53:1.44-1.62** | **1.45:1.37-1.54** | 0.78:0.5-1.16 | 0.82:0.52-1.21 | 1.35:0.96-1.86 | 1.31:0.93-1.8 | **1.51:1.43-1.59** | **1.43:1.35-1.51** | **1.73:1.42-2.09** | **1.67:1.38-2.02** | **0.5:0.37-0.66** | **0.49:0.36-0.65** | 1.12:1.01-1.23 | 1.07:0.96-1.17 |
| 3 | **1.49:1.42-1.57** | **1.4:1.33-1.47** | **1.79:1.27-2.48** | **1.84:1.3-2.55** | **1.45:1.37-1.54** | **1.36:1.29-1.45** | 1.23:0.83-1.78 | 1.26:0.85-1.83 | 1.54:1.08-2.15 | 1.47:1.03-2.05 | **1.47:1.4-1.56** | **1.38:1.31-1.46** | **1.36:1.01-1.66** | 1.31:1.06-1.6 | **0.41:0.29-0.57** | **0.41:0.28-0.56** | **1.21:1.09-1.34** | 1.14:1.03-1.27 |
| 4 | **1.48:1.4-1.56** | **1.4:1.33-1.48** | 1.38:0.9-2.06 | 1.38:0.9-2.06 | **1.49:1.4-1.59** | **1.42:1.33-1.51** | 1.38:0.91-2.02 | 1.39:0.91-2.04 | 1.48:0.99-2.15 | 1.4:0.94-2.04 | **1.52:1.43-1.61** | **1.44:1.36-1.53** | **1.45:1.17-1.79** | **1.41:1.13-1.74** | **0.51:0.36-0.72** | **0.5:0.35-0.71** | **1.20:1.07-1.35** | 1.15:1.02-1.29 |
| 5 | **1.55:1.47-1.63** | **1.49:1.42-1.57** | 1.46:0.94-2.19 | 0.99:0.96-1.02 | **1.59:1.5-1.69** | **1.53:1.44-1.63** | 1.21:0.78-1.8 | 1.26:0.81-1.87 | 1.69:1.08-2.56 | 1.65:1.05-2.49 | **1.53:1.44-1.62** | **1.47:1.38-1.56** | **1.41:1.12-1.75** | **1.38:1.1-1.72** | **0.62:0.44-0.86** | **0.61:0.43-0.84** | **1.23:1.07-1.39** | 1.18:1.04-1.35 |

**Supplementary Table 8C. Association of psychiatric diagnosis in blepharospasm in relation to controls**

|  | **Diagnosis overall** | | **ADHD** | | **Anxiety disorder** | | **ASD** | | **Conduct disorder** | | **Depression** | | **Eating disorder** | | **SMI** | | **SUD** | |
| --- | --- | --- | --- | --- | --- | --- | --- | --- | --- | --- | --- | --- | --- | --- | --- | --- | --- | --- |
|  | OR:95 % CI | OR:95 % CI^α^ | OR:95 % CI | OR:95 % CI ^α^ | OR:95 % CI | OR:95 % CI^α^ | OR:95 % CI | OR:95 % CI^α^ | OR:95 % CI | OR:95 % CI^α^ | OR:95 % CI | OR:95 % CI^α^ | OR:95 % CI | OR:95 % CI^α^ | OR:95 % CI | OR:95 % CI^α^ | OR:95 % CI | OR:95 % CI^α^ |
| Blepharospasm | **1.47:1.32-1.65** | **1.44:1.29-1.62** | 0.42:0.07-1.29 | 0.42:0.07-1.29 | **1.67:1.47-1.9** | **1.64:1.44-1.86** | 0.67:0.17-1.76 | 0.68:0.17-1.78 | 0.62:0.15-1.61 | 0.61:0.15-1.58 | **1.37:1.21-1.55** | **1.34:1.18-1.52** | 1.1:0.63-1.76 | 1.08:0.62-1.74 | 0.87:0.48-1.44 | 0.86:0.47-1.42 | 1.15:0.9-1.43 | 1.12:0.88-1.4 |
| *Sex* |  |  |  |  |  |  |  |  |  |  |  |  |  |  |  |  |  |  |
| Female | **1.41:1.22-1.62** | **1.4:1.21-1.61** | 0.86:0.05-2.84 | 0.86:0.05-3.84 | **1.58:1.35-1.84** | **1.57:1.35-1.84** | 1.66:0.27-5.19 | 1.67:0.28-5.23 | 0.95:0.16-2.97 | 0.95:0.16-2.96 | **1.27:1.09-1.47** | **1.25:1.08-1.46** | 0.81:0.39-1.48 | 0.81:0.39-1.47 | 0.6:0.24-1.23 | 0.61:0.24-1.24 | 1.15:0.82-1.56 | 1.14:0.82-1.55 |
| Male | **1.56:1.29-1.89** | **1.5:1.23-1.82** | 0.29:0.02-1.29 | 0.29:0.02-1.29 | **1.84:1.44-2.31** | **1.76:1.38-2.22** | 0.33:0.02-1.45 | 0.33:0.02-1.47 | 0.38:0.02-1.68 | 0.37:0.02-1.63 | **1.56:1.24-1.95** | **1.5:1.191.87** | 2.06:0.81:4.23 | 2.01:0.79-4.13 | 1.36:0.58-2.65 | 1.33:0.57-2.59 | 1.18:0.83-1.62 | 1.13:0.8-1.55 |
| *Age at index date* | | | | | | |  |  |  |  |  |  |  |  |  |  |  |  |
| < 20 years | 0.89:0.56-1.36 | 0.9:0.56-1.39 | - | - | 1.09:0.59-1.83 | 1.11:0.61-1.9 | 1.433:0.24-4.52 | 1.44:0.24-4.53 | 2.24:0.55-5.94 | 2.25:0.55-5.98 | 0.66:0.33-1.16 | 0.66:0.33-1.19 | 0.62:0.04-2.79 | 0.63:0.04-2.81 | 3.3:0.54-10.43 | 3.38:0.56-10.74 | 0.75:0.23-1.79 | 0.77-0.24-1.84 |
| ≥ 20 years | **1.41:1.25-1.59** | **1.39:1.23-1.56** | 1.96:0.32-6.14 | 1.98:0.33-6.2 | **1.57:1.38-1.79** | **1.55:1.36-1.77** | 0.9:0.05-4 | 0.89:0.05-3.98 | - | - | **1.29:1.13-1.47** | **1.27:1.12-1.44** | 1.22:0.68-1.98 | 1.2:0.68-1.96 | 0.68:0.35-1.18 | 0.68:0.35-1.17 | 1.13:0.88-1.41 | 1.11:0.89-1.4 |
| *WIMD at index date* | | | | | | |  |  |  |  |  |  |  |  |  |  |  |  |
| 1 | 1.54:1.11-2.12 | 1.58:1.14-2.19 | **-** | **-** | **1.72:1.2-2.41** | **1.78:1.24-2.51** | - | **-** | 2.19:0.36-6.91 | 2.24:0.37-7.08 | 1.29:0.91-1.81 | 1.33:0.93-1.87 | 1.05:0.17-3.29 | 1.06:0.17-3.32 | 1.19:0.29-3.15 | 1.21:0.3-3.19 | 1.54:0.92-2.43 | 1.58:0.94-2.5 |
| 2 | **1.7:1.31-2.21** | **1.64:1.26-2.14** | - | **-** | **1.98:1.48-2.62** | **1.92:1.43-2.54** | - | **-** | - | - | **1.67:1.26-2.19** | **1.61:1.21-2.12** | 1.21:0.3-3.2 | 1.19:0.29-3.13 | 0.97:0.24-2.56 | 0.96:0.24-2.53 | 1.12:0.65-1.8 | 1.08:0.62-1.74 |
| 3 | **1.53:1.2-1.94** | **1.51:1.19-1.92** | **-** | **-** | 1.43:1.07-1.88 | 1.41:1.05-1.87 | - | **-** | 1.05:0.06-4.68 | 1.03:0.06-4.63 | 1.37:1.05-1.78 | 1.36:1.03-1.76 | 1.67:0.59-3.65 | 1.65:0.59-3.61 | 0.97:0.24-2.54 | 0.96:0.24-2.53 | 1.26:0.74-1.99 | 1.24:0.73-1.97 |
| 4 | **1.46:1.13-1.86** | **1.42:1.11-1.82** | - | **-** | **2.05:1.56-2.66** | **2.01:1.53-2.62** | - | **-** | **-** | **-** | 1.41:1.07-1.85 | 1.38:1.04-1.81 | 1.73:0.62-3.79 | 1.7:0.6-3.72 | 0.76:0.13-2.37 | 0.75:0.12-2.34 | 0.82:0.41-1.46 | 0.8:0.4-1.43 |
| 5 | **1.55:1.23-1.94** | **1.5:1.18-1.88** | - | **-** | **1.6:1.22-2.08** | **1.55:1.18-2.02** | - | **-** | **-** | **-** | **1.44:1.11-1.86** | 1.39:1.07-1.8 | **-** | **-** | 0.73:0.12-2.29 | 0.72:0.12-2.25 | 1.61:0.93-2.58 | 1.57:0.91-2.51 |

**Supplementary Table 8D. Association of psychiatric diagnosis in tremor in relation to controls**

|  | **Diagnosis overall** | | **ADHD** | | **Anxiety disorder** | | **ASD** | | **Conduct disorder** | | **Depression** | | **Eating disorder** | | **SMI** | | **SUD** | |
| --- | --- | --- | --- | --- | --- | --- | --- | --- | --- | --- | --- | --- | --- | --- | --- | --- | --- | --- |
|  | OR:95 % CI | OR:95 % CI^α^ | OR:95 % CI | OR:95 % CI ^α^ | OR:95 % CI | OR:95 % CI^α^ | OR:95 % CI | OR:95 % CI^α^ | OR:95 % CI | OR:95 % CI^α^ | OR:95 % CI | OR:95 % CI^α^ | OR:95 % CI | OR:95 % CI^α^ | OR:95 % CI | OR:95 % CI^α^ | OR:95 % CI | OR:95 % CI^α^ |
| Tremor | **2.27:2.2-2.35** | **2.32-2.24-2.4** | 1.34:1.05-1.7 | 1.34:1.05-1.7 | **2.31:2.22-2.39** | **2.35:2.26-2.44** | **1.57:1.24-1.97** | **1.58:1.24-1.98** | 1.31:1.02-1.66 | 1.32:1.02-1.66 | **1.95:1.88-2.02** | **1.98:1.91-2.06** | **2.34:2.09-2.61** | **2.34:2.08-2.61** | **1.39:1.21-1.58** | **1.39:1.21-1.58** | **1.85:1.74-1.96** | **1.86:1.75-1.97** |
| *Sex* |  |  |  |  |  |  |  |  |  |  |  |  |  |  |  |  |  |  |
| Female | **2.25:2.15-2.35** | **2.29:2.19-2.39** | **2.05:1.32-3.05** | **2.05:1.32-3.05** | **2.28:2.17-2.38** | **2.31:2.2-2.42** | **1.89:1.2-2.82** | **1.9:1.21-2.84** | 1.27:0.84-1.83 | 1.27:0.84-1.83 | **1.85:1.77-1.94** | **1.88:1.79-1.96** | **2.34:2.05-2.67** | **2.34:2.05-2.66** | **1.51:1.28-1.78** | **1.51:1.28-1.78** | **1.7:1.56-1.85** | **1.7:1.56-1.85** |
| Male | **2.43:2.3-2.57** | **2.48:2.35-2.62** | 1.11:0.82-1.47 | .11:0.82-1.47 | **2.56:2.4-2.73** | **2.61:2.45-2.78** | 1.43:1.07-1.87 | 1.43:1.07-1.87 | 1.33:0.96-1.78 | 1.33:0.96-1.79 | **2.31:2.17-2.45** | **2.35:2.21-2.5** | **2.42:1.92-3.01** | **2.42:1.92-3.01** | 1.19:0.94-1.49 | 1.19:0.94-1.49 | **1.98:1.82-2.14** | **2:1.84-2.16** |
| *Age at index date* | | | | | | |  |  |  |  |  |  |  |  |  |  |  |  |
| < 20 years | **1.99:1.82-2.16** | **2.23:2.03-2.43** | **1.98:1.51-2.56** | **2.0:1.52-1.58** | **2.22:2-2.46** | **2.44:2.2-2.71** | **2.08:1.56-2.71** | **2.06:1.55-2.69** | **1.84:1.35-2.44** | **1.89:1.39-2.51** | **1.72:1.55-1.9** | **1.88:1.69-2.09** | **1.99:1.51-2.57** | **2.06:1.56-2.65** | 1.65:1.01-2.54 | 1.72:1.06-2.65 | **1.3:1.08-1.55** | **1.36:1.13-1.63** |
| ≥ 20 years | **2.24:2.16-2.33** | **2.26:2.18-2.35** | 1.13:0.59-1.94 | 1.13:0.59-1.95 | **2.22:2.14-2.32** | **2.24:2.15-2.33** | **1.9:1.18-2.9** | **1.89:1.18-2.89** | 1.29:0.82-1.94 | 1.3:0.82-1.94 | **1.9:1.83-1.97** | **1.91:1.84-1.99** | **2.49:2.19-2.82** | **2.48:2.19-2.81** | **1.29:1.11-1.48** | **1.28:1.11-1.48** | **1.89:1.77-2.01** | **1.89:1.78-2.01** |
| *WIMD at index date* | | | | | | |  |  |  |  |  |  |  |  |  |  |  |  |
| 1 | **2.27:2.1-2.44** | **2.32:2.15-2.5** | 1.32:0.84-1.98 | 1.32:0.84-1.99 | **2.27:2.1-2.45** | **2.31:2.14-2.5** | 1.23:0.68-2.05 | 1.23:0.68-2.05 | 1.18:0.74-1.78 | 1.18:0.74-1.78 | **1.81:1.68-1.95** | **1.84:1.7-1.99** | **2.23:1.75-2.79** | **2.22:1.75-2.78** | 1.11:0.84-1.45 | 1.11:0.84-1.44 | **1.63:1.46-1.82** | **1.63:1.46-1.82** |
| 2 | **2.2:2.04-2.37** | **2.26:2.1-2.44** | 1.22:0.71-1.94 | 1.22:0.71:1.94 | **2.32:2.14-2.51** | **2.38:2.2-2.58** | 1.76:1.11-2.66 | 1.77:1.12-2.67 | 1.38:0.82-2.17 | 1.38:0.82-2.18 | **1.9:1.76-2.05** | **1.96:1.8-2.11** | **2.1:1.61-2.7** | **2.11:1.61-2.71** | 1.35:1.01-1.77 | 1.35:1.01-1.78 | **1.67:1.47-1.89** | **1.69:1.49:1.92** |
| 3 | **2.37:2.19-2.55** | **2.42:2.24-2.61** | 1.51:0.83-2.52 | 1.51:0.83-2.53 | **2.36:2.17-2.56** | **2.4:2.21-2.61** | 1.88:1.11-2.99 | 1.88:1.12-2.99 | 1.22:0.64-2.1 | 1.22:0.64-2.1 | **2.01:1.86-2.18** | **2.04:1.88-2.22** | **2.52:1.96-3.19** | **2.52:1.96-3.19** | 1.45:1.05-1.94 | 1.45:1.05-1.94 | **2.08:1.82-2.36** | **2.09:1.83-2.37** |
| 4 | **2.26:2.09-2.45** | **2.28:2.1-2.47** | 1.45:0.732.56 | 1.45:0.74-2.57 | **2.3:2.11-2.52** | **2.32:2.12-2.54** | 1.83:1.03-3.03 | 1.84:1.03-3.04 | 1.35:0.69-2.39 | 1.35:0.69-2.39 | **1.99:1.82-2.16** | **1.99:1.83-2.17** | **2.76:2.14-3.52** | **2.75:2.13-3.51** | 1.38:0.95-1.93 | 1.38:0.95-1.93 | **2.04:1.75-2.35** | **2.03:1.75-2.35** |
| 5 | **2.25:2.08-2.43** | **2.28:2.11-2.47** | 1.15:0.51-2.21 | 1.15:0.52-2.22 | **2.24:2.05-2.44** | **2.27:2.07-2.48** | 1.08:0.51-2.0 | 1.09:0.51-2.01 | 1.5:0.7-2.81 | 1.5:0.7-2.81 | **2.07:1.9-2.25** | **1.07:1.06-1.07** | **2.08:1.55-2.75** | **2.08:1.55-2.75** | **1.85:1.33-2.52** | **1.85:1.33-2.51** | **2.03:1.71-2.38** | **2.03:1.71-2.38** |

**Supplementary Table 8E. Association of psychiatric diagnosis in other dystonia in relation to controls**

|  | **Diagnosis overall** | | **ADHD** | | **Anxiety disorder** | | **ASD** | | **Conduct disorder** | | **Depression** | | **Eating disorder** | | **SMI** | | **SUD** | |
| --- | --- | --- | --- | --- | --- | --- | --- | --- | --- | --- | --- | --- | --- | --- | --- | --- | --- | --- |
|  | OR:95 % CI | OR:95 % CI^α^ | OR:95 % CI | OR:95 % CI ^α^ | OR:95 % CI | OR:95 % CI^α^ | OR:95 % CI | OR:95 % CI^α^ | OR:95 % CI | OR:95 % CI^α^ | OR:95 % CI | OR:95 % CI^α^ | OR:95 % CI | OR:95 % CI^α^ | OR:95 % CI | OR:95 % CI^α^ | OR:95 % CI | OR:95 % CI^α^ |
| Other | **1.99:1.69-2.35** | **2.1:1.78-2.48** | **3.67:1.67-6.91** | **3.66:1.66-6.89** | **1.73:1.43-2.08** | **1.82:1.5-2.19** | 2.97:1.17-6.07 | 2.94:1.16-6.01 | **3.19:1.36-6.22** | **3.28:1.4-6.41** | **1.84:1.55-2.19** | **1.95:1.63-2.32** | 0.64:0.2-1.49 | 0.65:0.2-1.52 | **3.61:2.33-5.32** | **3.67:2.37-5.4** | **1.98:1.49-2.57** | **2.05:1.55-2.66** |
| *Sex* |  |  |  |  |  |  |  |  |  |  |  |  |  |  |  |  |  |  |
| Female | **1.75:1.42-2.16** | **1.84:1.49-2.29** | **8.04:2.47-19.09** | **8.03:2.46-19.07** | **1.71:1.36-2.15** | **1.8:1.43-2.27** | **5.77:1.42-15.24** | **5.72:1.41-15.11** | 2.21:0.37-6.89 | 2.31:0.38-7.23 | **1.57:1.25-1.95** | **1.65:1.32-2.06** | 0.84:0.26-1.95 | 0.85:0.26-2 | 2.14:1.02-3.91 | 2.18:1.04-3.98 | 0.99:0.56-1.6 | 1.02:0.58-1.66 |
| Male | **2.52:1.94-3.26** | **2.64:2.03-3.44** | 2.37:0.73-5.59 | 2.37:0.73-5.59 | **1.84:1.31-2.54** | **1.92:1.35-2.65** | 1.99:0.49-5.23 | 1.98:0.49-5.2 | **3.88:1.38-8.49** | **3.96-1.4-8.67** | **2.59:1.95-3.42** | **2.72:2.04-3.6** | - | - | **6.13:3.47-10.03** | **6.22:3.51-10.16** | **3.1:2.2-4.26** | **3.23:2.29-4.46** |
| *Age at index date* | | | | | | |  |  |  |  |  |  |  |  |  |  |  |  |
| < 20 years | **3.18:2.07-4.89** | **4.34:2.75-6.84** | **5.93:2.3-12.55** | **6.11:2.37-12.95** | **2.82:1.68-4.54** | **3.64:2.13-6.02** | 3.21:0.79-8.61 | 3.14:0.77-8.42 | **5.66:1.98-12.67** | **6.32:.221-14.22** | **2.09:1.32-3.39** | **2.73:1.57-4.55** | 0.92:0.05-4.13 | 1.03:0.06-4.64 | **12.62:4.41-28.4** | **15.16:525-34.57** | **4.11:2.17-7.18** | **5.08:2.5-9.02** |
| ≥ 20 years | **1.74:1.46-2.08** | **1.8:1.5-2.15** | 4.52:0.75-14.21 | 4.49:0.74-14.12 | **1.52:1.24-1.85** | **1.57:1.27-1.91** | **6.24:1.54-16.51** | **6.28:1.55-16.6** | 2.71:0.45-8.48 | 2.81:0.46-8.79 | **1.7:1.41-2.04** | **1.75:1.45-2.11** | 0.6:0.15-1.56 | 0.6:0.15-1.57 | **2.81:171-4.32** | **2.82:1.72-4.34** | **1.66:1.21-2.23** | **1.7:1.24-2.28** |
| *WIMD at index date* | | | | | | |  |  |  |  |  |  |  |  |  |  |  |  |
| 1 | **1.96:1.4-2.77** | **2.18:1.54-3.1** | 4:0.98-10.66 | 4.01:0.98-1.68 | 1.49:1.01-2.16 | 1.63:1.09-2.37 | 2.01:0.11-9.06 | 0.99:0.96-1.02 | 2.47:0.41-7.81 | 2.63:0.43-8.34 | 1.5:1.05-2.13 | 1.65:1.14-2.36 | 0.59:0.03-2.62 | 0.61:0.03-2.73 | **3.24:1.37-6.45** | **3.39:1.43-6.77** | **2.45:1.55-3.73** | **2.65:1.67-2.04** |
| 2 | **2.11:1.48-3.01** | **2.16:1.51-3.1** | 3.46:0.57-10.96 | 3.45:0.57-10.96 | 1.57:1.04-2.33 | 1.6:1.05-2.38 | 3.69:0.61-11.71 | 3.69:0.61-11.72 | 1.83:0.1-8.23 | 1.84:0.1-8.27 | **1.85:1.27-2.65** | **1.89:1.3-2.74** | 0.74:0.04-3.3 | 0.74:0.04-3.31 | **3.7:1.44-7.73** | **3.7:1.45-7.74** | 1.73:0.93-2.96 | 1.75:0.94-3.0 |
| 3 | **2.18:1.49-3.2** | **2.39:1.62-3.53** | 2.99:0.17-13.54 | 2.95:0.17-13.35 | **2.06:1.33-3.11** | **2.25:1.45-3.42** | - | - | 5.69:0.93-18.16 | 6.03:0.99-19.28 | **2.18:1.45-3.22** | **2.39:1.58-3.55** | 0.89:0.05-3.98 | 0.93:0.05-4.16 | **6.41:2.69-12.88** | **6.52:2.74-13.12** | 1.84:0.86-3.44 | 1.95:0.91-3.66 |
| 4 | 1.49:0.97-2.24 | 1.53:0.99-2.31 | 3.6:0.2-16.34 | 3.58:0.2-16.28 | 1.48:0.88-2.38 | 1.53:0.9-2.46 | 6.76:1.11-21.66 | 6.73:1.1-21.59 | 3.37:0.19-15.29 | 3.45:0.2-15.7 | 1.45:0.9-2.27 | 1.49:0.92-2.34 | 1:0.06-4.48 | 1.01:0.06-4.55 | 2.23:0.37-7.06 | 2.24:0.37-7.72 | 0.96:0.29-2.29 | 0.97:0.3-2.34 |
| 5 | **2.11:1.46-3.03** | **2.17:1.5-3.13** | 3.37:0.19-15.29 | 3.37:0.19-15.27 | **2.09:1.37-3.11** | **1.07:1.07-1.08** | 2.81:0.16-12.69 | 2.8:0.16-12.64 | 3.91:0.22-17.78 | 3.94:0.22-17.92 | **2.3:1.55-3.35** | **2.37:1.59-3.47** | **-** | **-** | 2.014:0.33-6.36 | 2.02:0.33-6.4 | 2.24:1-4.31 | 2.27:1.01-4.37 |
