# Supplementary material for: Longitudinal analysis of the relationship between motor and psychiatric symptoms in idiopathic dystonia
Source: Eur J Neurol. 2022 Sep 11;29(12):3513–27. doi: 10.1111/ene.15530 (PMC9826317; doi:10.1111/ene.15530)
Supplement: Supplementary file 11 — TABLE S9 [file ENE-29-3513-s003.docx]

**Supplementary Table 9A. Association of psychiatric prescription in dystonia in relation to controls**

|  | **Medication**  **overall** | | **Antidepressant** | | **Anxiolytic** | | **Antipsychotic** | | **Hypnotic** | |
| --- | --- | --- | --- | --- | --- | --- | --- | --- | --- | --- |
|  | OR:95 % CI | OR:95 % CI^α^ | OR:95 % CI | OR:95 % CI^α^ | OR:95 % CI | OR:95 % CI^α^ | OR:95 % CI | OR:95 % CI^α^ | OR:95 % CI | OR:95 % CI^α^ |
| Dystonia | **1.35:1.33-1.38** | **1.3:1.27-1.32** | **1.44:1.41-1.47** | **1.39:1.36-1.42** | **1.67:1.63-1.71** | **1.61:1.57-1.65** | **0.78:0.73-0.83** | **0.77:0.72-0.82** | **1.34:1.3-1.38** | **1.3:1.26-1.33** |
| *Sex* |  |  |  |  |  |  |  |  |  |  |
| Female | **1.4:1.36-1.43** | **1.33:1.3-1.37** | **1.45:1.42-1.49** | **1.39:1.35-1.43** | **1.74:1.69-1.79** | **1.68:1.63-1.73** | **0.79:0.73-0.86** | **0.78:0.72-0.85** | **1.34:1.3-1.39** | **1.3:1.26-1.34** |
| Male | **1.32:1.28-1.36** | **1.27:1.23-1.32** | **1.47:1.42-1.52** | **1.43:1.38-1.48** | **1.55:1.49-1.62** | **1.5:1.44-1.57** | **0.76:0.69-0.85** | **0.75:0.67-0.83** | **1.34:1.27-1.4** | **1.3:1.24-1.36** |
| *Age at index date* |  |  |  |  |  |  |  |  |  |  |
| <20 years | **1.33:1.26-1.39** | **1.29:1.23-1.36** | **1.33:1.26-1.4** | **1.29:1.23-1.36** | **1.51:1.39-1.64** | **1.46:1.35-1.59** | 1.05:0.87-1.26 | 1.02:0.84-1.22 | **1.36:1.24-1.48** | **1.32:1.2-1.44** |
| ≥ 20 years | **1.41:1.38-1.44** | **1.35:1.32-1.38** | **1.51:1.48-1.54** | **1.45:1.42-1.48** | **1.73:1.69-1.78** | **1.67:1.63-1.72** | **0.76:0.71-0.81** | **0.75:0.7-0.81** | **1.36:1.32-1.4** | **1.32:1.28-1.36** |
| *WIMD at index date* |  |  |  |  |  |  |  |  |  |  |
| 1 | **1.42:1.36-1.48** | **1.35:1.29-1.41** | **1.42:1.36-1.48** | **1.35:1.29-1.41** | **1.77:1.68-1.87** | **1.71:1.62-1.8** | **0.78:0.69-0.88** | **0.76:0.67-0.86** | **1.4:1.32-1.48** | **1.35:1.27-1.43** |
| 2 | **1.37:1.32-1.43** | **1.32:1.27-1.38** | **1.46:1.4-1.53** | **1.41:1.35-1.48** | **1.77:1.68-1.87** | **1.73:1.64-1.82** | **0.78:0.68-0.89** | **0.77:0.67-0.88** | **1.34:1.27-1.42** | **1.3:1.23-1.38** |
| 3 | **1.33:1.27-1.38** | **1.26:1.21-1.32** | **1.45:1.38-1.51** | **1.38:1.32-1.44** | **1.63:1.55-1.72** | **1.57:1.49-1.65** | **0.79:0.69-0.91** | **0.78:0.68-0.9** | **1.32:1.24-1.4** | **1.27:1.2-1.35** |
| 4 | **1.36:1.3-1.42** | **1.31:1.25-1.37** | **1.48:1.41-1.55** | **1.42:1.36-1.49** | **1.59:1.5-1.68** | **1.53:1.44-1.62** | **0.78:0.67-0.91** | **0.78:0.66-0.91** | **1.39:1.3-1.48** | **1.34:1.26-1.43** |
| 5 | **1.31:1.26-1.37** | **1.27:1.22-1.33** | **1.44:1.37-1.5** | **1.4:1.33-1.46** | **1.57:1.48-1.66** | **1.53:1.44-1.62** | 0.78:0.67-0.92 | 0.78:0.66-0.92 | **1.25:1.17-1.34** | **1.22:1.15-1.31** |

Bold values show statistically significant OR with Bonferroni correction (p = 0.005)

^α^ORs and CIs estimated by logistic regression and adjusted for time in study

**Abbreviations:** ADHD: Attention Deficit Hyperactive Disorder, ASD: Autism Spectrum Disorder, CI: 95% Confidence Intervals, OR: odds ratios, SMI: Severe Mental Illness, SUD: Substance Use Disorder

**Supplementary Table 9A. Association of psychiatric prescription in cervical dystonia in relation to controls**

|  | **Medication**  **overall** | | **Antidepressant** | | **Anxiolytic** | | **Antipsychotic** | | **Hypnotic** | |
| --- | --- | --- | --- | --- | --- | --- | --- | --- | --- | --- |
|  | OR:95 % CI | OR:95 % CI^α^ | OR:95 % CI | OR:95 % CI^α^ | OR:95 % CI | OR:95 % CI^α^ | OR:95 % CI | OR:95 % CI^α^ | OR:95 % CI | OR:95 % CI^α^ |
| Cervical dystonia | **1.19:1.16-1.22** | **1.11:1.09-1.14** | **1.29:1.26-1.32** | **1.21:1.18-1.24** | **1.56:1.52-1.6** | **1.48:1.43-1.52** | **0.57:0.52-0.61** | **0.54:0.5-0.6** | **1..17:1.13-1.21** | **1.11:1.08-1.15** |
| *Sex* |  |  |  |  |  |  |  |  |  |  |
| Female | **1.28:1.24-1.31** | **1.19:1.15-1.22** | **1.35:1.32-1.39** | **1.27:1.23-1.3** | **1.66:1.61-1.72** | **1.58:1.52-1.63** | **0.57:0.51-0.64** | **0.56:0.5-0.62** | **1.21:1.16-1.26** | **1.15:1.1-1.19** |
| Male | 1.06:1.02-1.1 | 1:0.96-1.04 | **1.18:1.13-1.23** | **1.11:1.07-1.16** | **1.35:1.28-1.42** | **1.28:1.21-1.35** | **0.53:0.45-0.61** | **0.51:0.44-0.59** | 1.09:1.03-1.16 | 1.04:0.98-1.11 |
| *Age at index date* |  |  |  |  |  |  |  |  |  |  |
| <20 years | **1.24:1.17-1.31** | **1.17:1.11-1.23** | **1.24:1.17-1.31** | **1.17:1.1-1.24** | **1.43:1.31-1.57** | **1.36:1.24-1.49** | 0.86:0.69-1.06 | 0.82:0.65-1.01 | **1.27:1.15-1.41** | **1.21:1.09-1.34** |
| ≥ 20 years | **1.29:1.26-1.33** | **1.21:1.18-1.24** | **1.4:1.37-1.44** | **1.32:1.28-1.35** | **1.7:1.65-1.76** | **1.61:1.56-1.66** | **0.54:0.49-0.6** | **0.54:0.49-0.59** | **1.12:1.19-1.28** | **1.17:1.13-1.21** |
| *WIMD at index date* |  |  |  |  |  |  |  |  |  |  |
| 1 | **1.25:1.19-1.32** | **1.15:1.09-1.21** | **1.27:1.21-1.34** | **1.17:1.11-1.23** | **1.64:1.54-1.74** | **1.54:1.45-1.64** | **0.57:0.48-0.67** | **0.54:0.46-0.64** | **1.26:1.18-1.35** | **1.19:1.11-1.27** |
| 2 | **1.22:1.16-1.28** | **1.14:1.08-1.2** | **1.32:1.26-1.39** | **1.24:1.18-1.31** | **1.66:1.56-1.76** | **1.57:1.48-1.68** | **0.65:0.55-0.77** | **0.63:0.53-0.75** | **1.18:1.09-1.26** | **1.12:1.04-1.2** |
| 3 | **1.16:1.11-1.22** | **1.08:1.03-1.13** | **1.29:1.23-1.36** | **1.2:1.14-1.26** | **1.52:1.43-1.61** | **1.42:1.134-1.51** | **0.51:0.42-0.62** | **0.51:0.41-0.62** | **1.13:1.06-1.21** | 1.07:0.99-1.15 |
| 4 | **1.18:1.12-1.25** | **1.11:1.06-1.18** | **1.31:1.24-1.38** | **1.24:1.17-1.31** | **1.5:1.4-1.6** | **1.42:1.32-1.51** | **0.51:0.41-0.64** | **0.51:0.4-0.63** | **1.19:1.1-1.28** | **1.14:1.05-1.23** |
| 5 | **1.16:1.1-1.22** | **1.11:1.05-1.17** | **1.28:1.21-1.35** | **1.22:1.16-1.29** | **1.5:1.4-1.6** | **1.43:1.34-1.53** | **0.51:0.41-0.64** | **0.51:0.4-0.64** | 1.1:1.02-1.19 | 1.06:0.98-1.15 |

**Supplementary Table 9C. Association of psychiatric prescription in blepharospasm in relation to controls**

|  | **Medication**  **overall** | | **Antidepressant** | | **Anxiolytic** | | **Antipsychotic** | | **Hypnotic** | |
| --- | --- | --- | --- | --- | --- | --- | --- | --- | --- | --- |
|  | OR:95 % CI | OR:95 % CI^α^ | OR:95 % CI | OR:95 % CI^α^ | OR:95 % CI | OR:95 % CI^α^ | OR:95 % CI | OR:95 % CI^α^ | OR:95 % CI | OR:95 % CI^α^ |
| Blepharospasm | **1.38:1.24-1.55** | **1.35:1.2-1.51** | **1.37:1.22-1.54** | **1.34:1.19-1.5** | **1.45:1.26-1.67** | **1.42:1.23-1.64** | 0.96:0.67-1.32 | 0.95:0.66-1.31 | **1.36:1.16-11.58** | **1.33:1.14-1.55** |
| *Sex* |  |  |  |  |  |  |  |  |  |  |
| Female | **1.29:1.12-1.49** | **1.28:1.11-1.47** | **1.27:1.1-1.46** | **1.26:1.09-1.45** | **1.46:1.24-1.72** | **1.46:1.23-1.72** | 0.93:0.59-1.39 | 0.93:0.59-1.39 | 1.29:1.07-1.55 | 1.28:1.07-1.54 |
| Male | **1.52:1.25-1.83** | **1.45:1.19-1.75** | **1.53:1.25-1.87** | **1.47:1.19-1.79** | 1.36:1.02-1.77 | 1.3:0.98-1.69 | 0.99:0.53-1.68 | 0.97:0.51-1.64 | 1.46:1.1-1.9 | 1.41:1.06-1.84 |
| *Age at index date* |  |  |  |  |  |  |  |  |  |  |
| <20 years | 0.91:0.54-1.45 | 0.93:0.55-1.51 | 0.78:0.43-1.32 | 0.8:0.44-1.38 | 0.96:0.34-2.12 | 1:0.35-2.23 | 1.58:0.26-4.97 | 1.61:0.26-5.09 | 0.64:0.16-1.7 | 0.65:0.16-1.74 |
| ≥ 20 years | 1.19:1.06-1.34 | 1.16:1.03-1.31 | **1.22:1.08-1.37** | 1.19:1.06-1.35 | **1.26:1.09-1.46** | 1.24:1.07-1.43 | 0.83:0.57-1.15 | 0.82:0.57-1.15 | 1.2:1.02-1.39 | 1.18:1.01-1.37 |
| *WIMD at index date* |  |  |  |  |  |  |  |  |  |  |
| 1 | 1.05:0.76-1.45 | 1.07:0.77-1.48 | 1.08:0.78-1.5 | 1.11:0.79-1.55 | 1.63:1.1-2.35 | 1.69:1.13-2.44 | 0.89:0.32-1.96 | 0.9:0.32:1.98 | 1.25:0.8-1.88 | 1.28:0.82-1.93 |
| 2 | **1.76:1.35-2.29** | **1.69:1.3-2.21** | **1.77:1.36-2.3** | **1.7:1.3-2.23** | **1.73:1.25-2.34** | **1.67:1.21-2.27** | 0.83:0.33-1.71 | 0.81:0.32-1.68 | 1.6:1.13-2.21 | 1.55:1.09-2.14 |
| 3 | 1.36:1.07-1.72 | 1.34:1.05-1.7 | 1.24:0.96-1.58 | 1.22:0.94-1.56 | 1.24:0.9-1.67 | 1.22:0.88-1.66 | 0.66:0.24-1.44 | 0.66:0.23-1.43 | 1.37:0.98-1.86 | 1.35:0.97-1.84 |
| 4 | **1.55:1.22-1.96** | **1.51:1.19-1.92** | **1.63:1.27-2.07** | **1.59:1.24-2.03** | 1.52:1.12-2.04 | 1.49:1.09-2 | 1.37:0.65-2.52 | 1.37:0.65-2.5 | 1.4:0.99-1.92 | 1.37:0.97-1.89 |
| 5 | 1.41:1.13-1.75 | 1.35:1.08-1.7 | 1.42:1.12-1.79 | 1.37:1.08-1.73 | 1.43:1.06-1.9 | 1.38:1.02-1.84 | 1.31:0.62-2.4 | 1.31:0.62-2.39 | 1.36:0.98-1.85 | 1.32:0.95-1.8 |

**Supplementary Table 9D. Association of psychiatric prescription in tremor in relation to controls**

|  | **Medication**  **overall** | | **Antidepressant** | | **Anxiolytic** | | **Antipsychotic** | | **Hypnotic** | |
| --- | --- | --- | --- | --- | --- | --- | --- | --- | --- | --- |
|  | OR:95 % CI | OR:95 % CI^α^ | OR:95 % CI | OR:95 % CI^α^ | OR:95 % CI | OR:95 % CI^α^ | OR:95 % CI | OR:95 % CI^α^ | OR:95 % CI | OR:95 % CI^α^ |
| Tremor | **1.83:1.77-1.89** | **1.86:1.8-1.93** | **1.89:1.83-1.95** | **1.93:1.86-1.99** | **1.94:1.86-2.02** | **1.97:1.89-2.05** | **1.3:1.19-1.43** | **1.3:1.19-1.43** | **1.78:1.7-1.86** | **1.79:1.71-1.87** |
| *Sex* |  |  |  |  |  |  |  |  |  |  |
| Female | **1.77:1.7-1.85** | **1.79:1.71-1.88** | **1.75:1.68-1.83** | **1.78:1.7-1.86** | **1.93:1.84-2.03** | **1.96:1.86-2.06** | **1.33:1.18-1.49** | **1.32:1.18-1.48** | **1.73:164-1.83** | **1.74:1.65-1.83** |
| Male | **2.05:1.95-2.17** | **2.1:1.99-2.22** | **2.3:2.17-2.43** | **2.36:2.23-2.49** | **2.05:1.91-2.19** | **2.08:1.94-2.23** | **1.27:1.09-1.47** | **1.27:1.09-1.47** | **1.93:1.8-2.08** | **1.94:1.81-2.09** |
| *Age at index date* |  |  |  |  |  |  |  |  |  |  |
| <20 years | **1.7:1.55-1.87** | **1.91:1.73-2.11** | **1.76:1.59-1.94** | **1.98:1.78-2.19** | **1.68:1.42-1.97** | **1.79:1.51-2.11** | **1.67:1.2-2.27** | **1.75:1.25-2.37** | **1.66:1.39-1.97** | **1.75:1.47-2.08** |
| ≥ 20 years | **1.73:1.67-1.8** | **1.74:1.67-1.81** | **1.8:1.73-1.86** | **1.81:1.74-1.88** | **1.83:1.76-1.91** | **1.84:1.77-1.92** | **1.2:1.09-1.32** | **1.2:1.09-1.31** | **1.67:1.59-1.74** | **1.67:1.6-1.75** |
| *WIMD at index date* |  |  |  |  |  |  |  |  |  |  |
| 1 | **1.87:1.73-2.01** | **1.9:1.76-2.05** | **1.82:1.69-1.96** | **1.86:1.72-2** | **2.04:1.87-2.22** | **2.06:1.9-2.25** | 1.22:1.02-1.45 | 1.21:1.01-1.45 | **1.7:1.55-1.86** | **1.7:1.55-1.86** |
| 2 | **1.79:1.66-1.93** | **1.85:1.71-1.99** | **1.83:1.7-1.98** | **1.9:1.76-2.05** | **2.04:1.87-2.23** | **2.09:1.92-2.28** | 1.1:0.89-1.34 | 1.1:0.9-1.35 | **1.77:1.61-1.94** | **1.8:1.64-1.98** |
| 3 | **1.86:1.73-2.01** | **1.89:1.75-2.04** | **1.95:1.8-2.1** | **1.99:1.84-2.15** | **1.97:1.81-2.16** | **2:1.83-2.19** | **1.45:1.19-1.76** | **1.45:1.19-1.76** | **1.83:1.66-2.01** | **1.84:1.67-2.02** |
| 4 | **1.89:1.75-2.05** | **1.91:1.76-2.07** | **1.96:1.81-2.13** | **1.98:1.82-2.15** | **1.83:1.66-2.01** | **1.84:1.67-2.03** | **1.45:1.15-1.8** | **1.45:1.15-1.79** | **1.96:1.77-2.16** | **1.96:1.77-2.16** |
| 5 | **1.72:1.59-1.86** | **1.74:1.61-1.88** | **1.87:1.72-2.02** | **1.89:1.75-2.05** | **1.74:1.58-1.92** | **1.76:1.59-1.94** | 1.36:1.07-1.71 | 1.36:1.07-1.7 | **1.64:1.47-1.82** | **1.64:1.47-1.82** |

**Supplementary Table 9E. Association of psychiatric prescription in other dystonia in relation to controls**

|  | **Medication**  **overall** | | **Antidepressant** | | **Anxiolytic** | | **Antipsychotic** | | **Hypnotic** | |
| --- | --- | --- | --- | --- | --- | --- | --- | --- | --- | --- |
|  | OR:95 % CI | OR:95 % CI^α^ | OR:95 % CI | OR:95 % CI^α^ | OR:95 % CI | OR:95 % CI^α^ | OR:95 % CI | OR:95 % CI^α^ | OR:95 % CI | OR:95 % CI^α^ |
| Other | **2.09:1.77-2.46** | **2.23:1.89-2.64** | **1.93:1.63-2.27** | **2.06:1.74-2.43** | **2.71:2.27-3.23** | **2.89:2.41-3.45** | **1.96:1.34-2.77** | **1.98:1.35-2.8** | **1.83:1.49-2.24** | **1.9:1.54-2.32** |
| *Sex* |  |  |  |  |  |  |  |  |  |  |
| Female | **1.86:1.5-2.31** | **1.98:1.6-2.48** | **1.64:1.32-2.02** | **1.74:1.41-2.16** | **2.61:2.09-3.25** | **2.79:2.22-3.49** | **1.7:1-2.69** | 1.72:1.01-2.71 | **1.56:1.19-2.01** | **1.62:1.24-2.09** |
| Male | **2.62:2.03-3.39** | **2.78:2.14-3.62** | **2.65:2.04-3.44** | **2.82:2.16-3.68** | **3.05:2.25-4.08** | **3.21:2.36-4.31** | **2.4:1.33-3.97** | **2.43:1.35-4.02** | **2.48:1.78-3.4** | **2.56:1.83-3.51** |
| *Age at index date* |  |  |  |  |  |  |  |  |  |  |
| <20 years | **3.22:2.06-4.97** | **4.74:2.93-7.61** | 1.55:0.88-2.58 | 2.05:1.13-3.52 | **7.58:4.51-12.23** | **10.68-6.18:17.82** | **6.03:2.11-13.51** | **7.17:2.5-16.25** | **4.7:2.48-8.21** | **5.83:3.04-10.37** |
| ≥ 20 years | **1.77:1.48-2.12** | **1.84:1.53-2.2** | **1.81:1.51-2.16** | **1.88:1.57-2.25** | **2.23:1.84-2.69** | **2.32:1.91-2.81** | 1.59:1.04-2.31 | 1.59:1.05-2.32 | **1.52:1.22-1.89** | **1.56:1.25-1.93** |
| *WIMD at index date* |  |  |  |  |  |  |  |  |  |  |
| 1 | **2.02:1.43-2.87** | **2.26:1.59-3.25** | 1.68:1.2-2.36 | **1.89:1.33-2.67** | **3.11:2.17-4.41** | **3.46:2.4-4.94** | 1.87:0.88-3.48 | 1.95:0.92-3.63 | **2.13:1.43-3.1** | **2.3:1.53-3.36** |
| 2 | **1.79:1.25-2.54** | **1.83:1.28-2.64** | 1.64:1.14-2.34 | 1.69:1.17-2.42 | **2.77:1.88-4.02** | **2.86:1.93-4.16** | 1.02:0.31-2.42 | 1.02:0.31-2.43 | 1.16:0.68-1.86 | 1.17:0.69-1.89 |
| 3 | **1.88:1.29-2.77** | **2.08:1.41-3.07** | **1.89:1.28-2.77** | **2.1:1.41-3.1** | **2.4:1.55-3.63** | **2.66:1.71-4.04** | **4.26:2.15-7.63** | **4.31:2.17-7.71** | 2.01:1.24-3.14 | **2.15:1.32-3.37** |
| 4 | **2.09:1.4-3.12** | **2.17:1.45-3.28** | 1.86:1.23-2.79 | **1.94:1.27-2.92** | **2.35:1.47-3.64** | **2.45:1.52-3.81** | 0.87:0.14-2.76 | 0.88:0.14-2.77 | 1.57:0.89-2.62 | 1.6:0.9-2.68 |
| 5 | **2.72:1.89-3.93** | **2.85:1.97-4.15** | **2.65:1.85-3.8** | **2.78:1.92-4.01** | **2.75:1.81-4.06** | **2.85:1.87-4.24** | 2.01:0.71-4.45 | 2.02:0.71-4.46 | **2.31:1.45-3.53** | **2.35:1.47-3.6** |
